# Combined Metal–Metal and Metal–Ligand Cooperativity in Dicopper-Catalyzed Azide–Alkyne Cycloaddition Reactions

**DOI:** 10.1021/acs.organomet.5c00387

**Published:** 2025-11-20

**Authors:** Cody B. van Beek, Hyoju Choi, Marije L. A. Hilberts, Marijn M. Lammertink, Bohyun Park, Martin Lutz, Mu-Hyun Baik, Daniël L. J. Broere

**Affiliations:** † Organic Chemistry and Catalysis, Institute for Sustainable Chemistry and Catalysis, Faculty of Science, 84889 Utrecht University, Universiteitsweg 99, Utrecht 3584 CG, The Netherlands; ‡ Department of Chemistry, 425259 Korea Advanced Institute of Science and Technology (KAIST), Daejeon 34141, Korea; § Center for Catalytic Hydrocarbon Functionalizations, 34968 Institute for Basic Science (IBS), Daejeon 34141, Korea; ∥ Structural Biochemistry, Bijvoet Centre for Biomolecular Research, Faculty of Science, 8125 Utrecht University, Universiteitsweg 99, Utrecht 3584 CG, The Netherlands

## Abstract

The mechanism of the copper-catalyzed azide–alkyne cycloaddition (CuAAC) reaction has been under investigation for over two decades. While catalytically relevant dicopper intermediates have been proposed and a few suspected intermediates have been isolated, the mechanism remains poorly understood. In this work, we describe the synthesis and characterization of neutral dicopper complexes bearing the proton-responsive dinucleating ^***iPr***^ **PNNP** “expanded pincer” ligand, which are demonstrated to be relevant intermediates in the CuAAC reaction. The acetylide complex [Cu_2_(^***iPr***^ PNNP*)­(μ-C≡C-*p*-F-C_6_H_4_)] (**2**) reacts with 1-azido-4-fluorobenzene at ambient temperature to form the dicopper complex [Cu_2_(^***iPr***^ PNNP*)­(μ-(1,4-bis­(*p*-fluorophenyl)-1,2,3-triazolide)] (**3**), featuring a symmetrically bridging 1,4-substituted 1,2,3-triazolide ligand. Mechanistic studies were performed using both isotopic labeling experiments and density functional theory (DFT) calculations for the subsequent protodemetalation step. These studies show that the release of the triazole product proceeds via a stepwise metal–ligand cooperative (MLC) pathway, which is favored over the direct alkyne-to-triazolide proton transfer as it requires less structural reorganization of the dicopper platform. This demonstrates how cooperativity between the copper centers and metal–ligand cooperativity can offer an alternative mechanistic pathway, bypassing the conventional rate-limiting alkyne-to-triazolide proton transfer in the CuAAC reaction.

## Graphical abstract

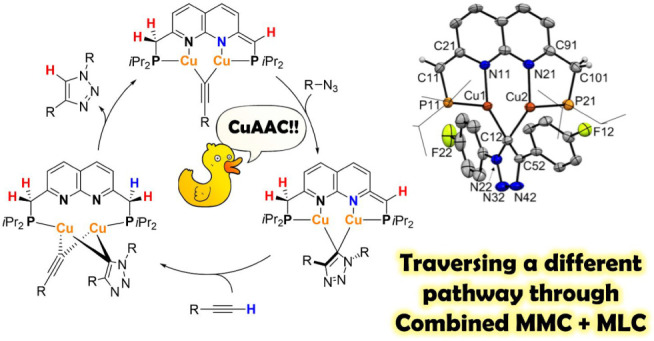

## Introduction

The copper­(I)-catalyzed azide–alkyne cycloaddition (CuAAC) reaction, introduced in the early 2000s, provides a robust, facile, and selective method for synthesizing 1,4-substituted 1,2,3-triazoles.^1^ Since its development, this reaction has garnered widespread attention and has been extensively applied in pharmacology, polymer science, and biochemistry.^2^ Numerous Cu­(I) complexes have been investigated under diverse conditions, with the most common system employing simple Cu­(II) salts in combination with sodium ascorbate to generate highly active Cu­(I) species.^3^ Despite extensive applications, the precise mechanism of the CuAAC reaction remains debated and has yet to be fully understood. A major challenge lies in the ambiguity regarding the nuclearity of the catalytically active copper species in solution. Aggregation phenomena involving copper acetylides,^4,5,6^ as well as the variability of alkyne and ligand coordination modes in copper complexes,^7^ have complicated mechanistic elucidation. Advances in kinetic studies,^8^ the isolation and characterization of proposed copper intermediates,^9^ labeling experiments,^10^ and computational investigations^11^ have led to the hypothesis that dicopper species are crucial intermediates in the catalytic cycle.

The currently accepted dinuclear mechanism of the CuAAC reaction is depicted in Scheme 1A. The reaction begins with the coordination of an organic azide to a dicopper acetylide species (i), which features either a symmetrically bridging or a σ,π-coordinated acetylide ligand.^11f,12^ This coordination promotes formation of the first C–N bond, yielding a six-membered metallacycle (ii). A subsequent ring contraction leads to the formation of a second C–N bond, generating a bridging triazolide complex. At this stage, one copper center may dissociate, potentially forming a mononuclear species (iii).^12^ In the final step, a new alkyne molecule coordinates to the copper center(s), accompanied by proton transfer from the alkyne to the triazolide ligand. This regenerates the dicopper acetylide complex and releases the 1,4-disubstituted 1,2,3-triazole product (iv). Computational studies have shown that, under aprotic conditions, this proton transfer occurs in a concerted fashion with alkyne deprotonation and presents a higher activation energy than the preceding cycloaddition step.^9a,13^ This concerted proton transfer has thus been suspected to be the rate-limiting step of the catalytic cycle. Although various dicopper intermediates have been proposed, their isolation has proven challenging, and only a few examples have been structurally characterized. A notable case is a dicopper triazole complex reported by Bertrand and coworkers, in which a 1,2,3-triazolide ligand binds in an asymmetric η^1^-fashion at the 3- and 5-positions to two nonequivalent copper­(I) centers supported by cyclic (alkyl)­amino carbene ligands (Scheme 1B).^9b^ This binding motif contrasts with the symmetrically bridging coordination modes commonly proposed in theoretical studies. In support of these computational models, Tilley and coworkers succeeded in isolating a symmetrically bridging dicopper triazolide complex using a rigid dinucleating ligand (2,7-bis­(fluoro-di­(2-pyridyl)-methyl)-1,8-naphthyridine (Scheme 1B).^9f^ More recently, a computational investigation by Héron and Balcells on a related dicopper system bearing a nonsymmetric phosphino-di­(pyridyl)­naphthyridine ligand also identified similar symmetrically bridging triazolide intermediates. Their study further reinforced the notion that the concerted proton transfer from the alkyne to the triazolide is the turnover-limiting step in the CuAAC mechanism (iv, Scheme 1A).^14^

**Scheme 1:**
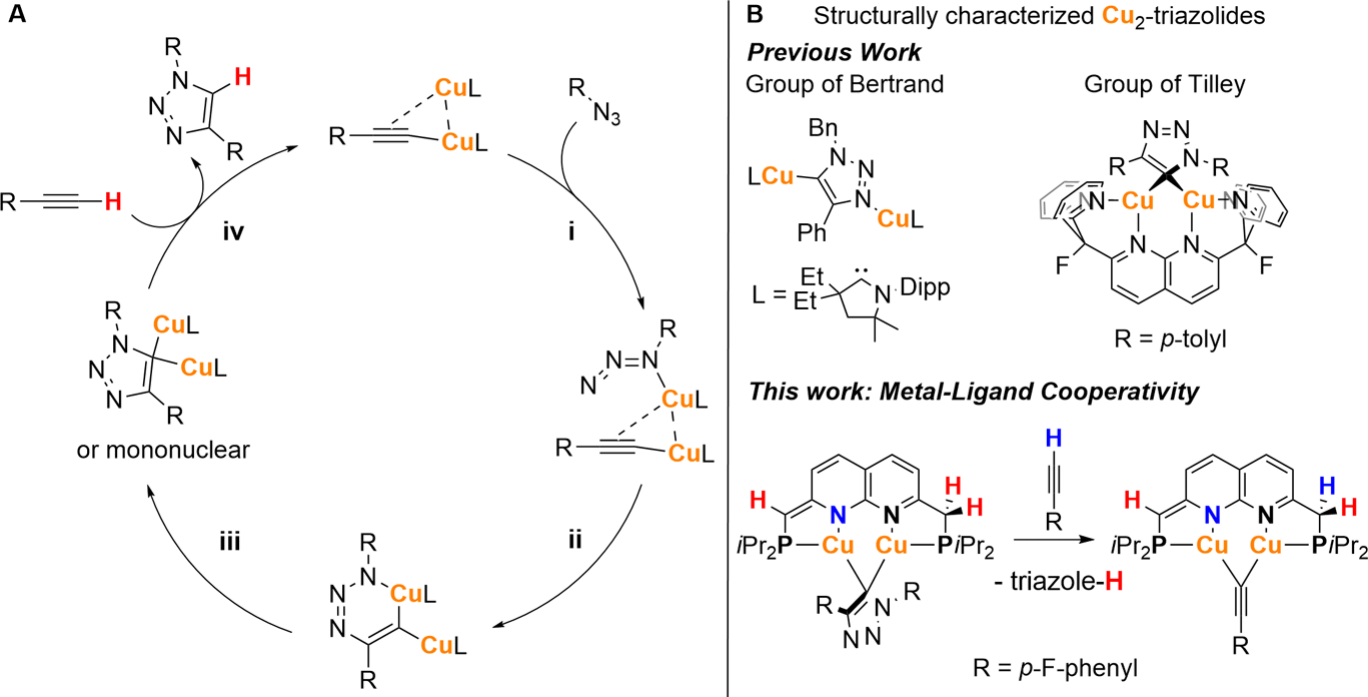
(A) Proposed CuAAC Mechanism for Dicopper Species; (B) Examples of Structurally Characterized Dicopper Triazolide Complexes

Our group has developed a proton-responsive, dinucleating PNNP ligand, which draws inspiration from the widely utilized PNP pincer ligand system and expands its chelating capabilities. This new ligand enables the synthesis of well-defined dinuclear metal complexes with closely positioned metal centers.^15^ The ligand’s methylene linkers exhibit mild acidity, allowing for reversible dearomatization of the naphthyridine core and thereby enabling bond activation via metal–ligand cooperativity (MLC).^16^ Motivated by Tilley’s successful isolation of CuAAC-relevant intermediates using a rigid bis­(dipyridyl)­naphthyridine dicopper platform, we sought to investigate whether analogous intermediates could be stabilized with a more flexible, lower-coordinate dicopper PNNP environment. We further hypothesized that the cooperative architecture of the expanded pincer scaffold might provide an alternative MLC-enabled pathway for the protodemetalation step,^17^ which is commonly identified as rate-determining in the CuAAC reaction. Herein, we report that the PNNP-supported dicopper platform allows for the isolation of well-defined bimetallic intermediates relevant to the CuAAC reaction, along with a unique tricopper cluster. Reactivity studies, spectroscopic characterizations, and DFT calculations confirm the relevance of these complexes and reveal that metal–ligand cooperativity facilitates an alternative mechanistic route, bypassing the conventional proton transfer step.

## Results and Discussion

We previously demonstrated that anionic dicopper acetylide complexes can be readily synthesized by reacting terminal alkynes with dicopper hydride species, accompanied by the evolution of dihydrogen.^16b^ Building on this approach, we hypothesized that analogous reactivity could be accessed using neutral dicopper mesityl complexes supported by the PNNP ligand, which are more conveniently prepared.^16a^ To this end, we targeted the synthesis of a neutral dicopper mesityl complex as a precursor to dicopper acetylides, employing the ^***i*** **Pr**^ **PNNP** ^18^ ligand owing to its enhanced solubility properties in the resulting complexes.

Treatment of a THF suspension of mesityl copper (2.1 equiv) with a THF solution of ^***i*** **Pr**^ **PNNP** ^18^ at ambient temperature resulted in the immediate formation of a dark red solution. This reaction afforded the dicopper mesityl complex [Cu_2_(^***i*** **Pr**^ PNNP*)­(μ-Mes)] (Mes = mesityl) (**1**), along with mesitylene as a byproduct (Scheme 2). Complex **1** was obtained as an air-sensitive, red solid by crystallization from pentane in 70% yield. All resonances of the ^1^H, ^13^C and ^31^P NMR spectra of **1** in C_6_D_6_ at 298 K were assigned using 2D NMR experiments (Figures S2–S8). The ^31^P­{^1^H} NMR spectrum displays two resonances at δ = 6.6 and −7.0 ppm, indicating two magnetically inequivalent phosphorus atoms in complex **1**. The ^1^H NMR spectrum featured two characteristic doublets at δ = 4.14 ppm (^2^ *J*_H,P_ = 2.7 Hz) and 2.39 ppm (^2^ *J*_H,P_ = 7.6 Hz) with an integral ratio of 1:2, corresponding to the methine and methylene linkers, respectively, suggesting the presence of a partially dearomatized ^***i*** **Pr**^ PNNP* ligand. This is supported by the four separate naphthyridine resonances observed between δ = 6.76 and 5.98 ppm, which are upfield-shifted compared to those in ^***i*** **Pr**^ **PNNP**.^15,16a,19^ Additionally, the isopropyl substituents showed two sets of resonances at δ = 1.73 and 1.31 ppm (both dhept −C*H*(CH_3_)_2_) and four methyl resonances between δ = 1.15–0.60 (overlapping dd, −CH­(C*H*_3_)_2_), indicating a nonsymmetric ^***i*** **Pr**^ PNNP* ligand. Three singlets at δ = 6.96, 2.88, and 2.30 ppm, integrating for 2, 6, and 3 protons, respectively, confirmed the presence of a single symmetrically bridging mesityl ligand on the NMR time-scale. The NMR characteristics of **1** suggest that it is an on average *C*_s_-symmetric compound, similar to the previously reported dicopper mesityl complex [Cu_2_(^***t*** **Bu**^ PNNP*)­(μ-Mes)],^16a^ indicating no significant structural changes between the -*t*Bu and -*i*Pr analogues.

**Scheme 2:**
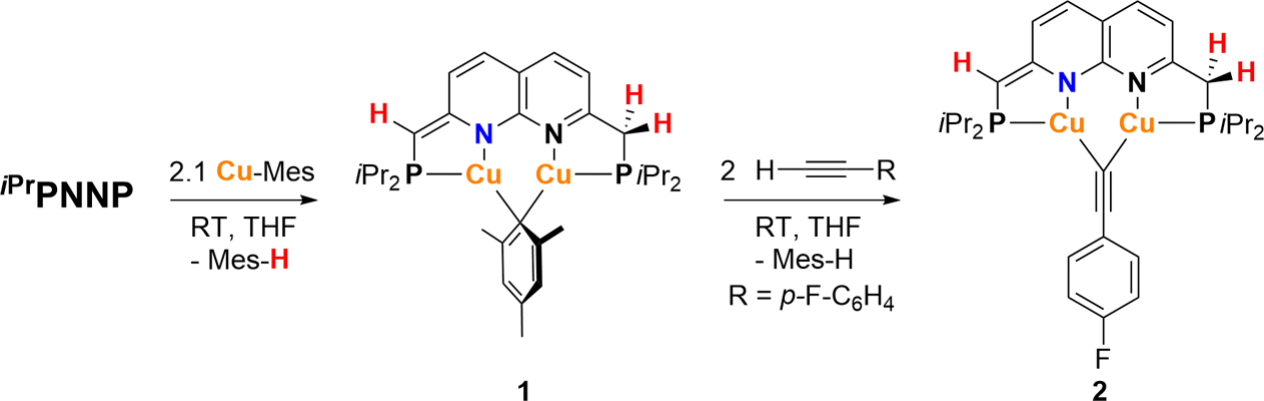
Synthesis of Dicopper Mesityl and Acetylide Complexes **1** and **2**

As anticipated, treatment of complex **1** with a terminal alkyne resulted in the formation of a dicopper acetylide complex, accompanied by elimination of mesitylene. Specifically, addition of 2 equiv^20^ of 1-ethynyl-4-fluorobenzene to a THF solution of complex **1** at ambient temperature led to the formation of a red solution. ^1^H NMR analysis revealed complete consumption of complex **1**, along with the formation of mesitylene and a new complex, identified as [Cu_2_(^***i*** **Pr**^ PNNP*)­(μ-C≡C-*p*–F-C_6_H_4_)] (**2**) (Scheme 2). Complex **2** was isolated as an air-sensitive red oil in 89% yield following extraction with pentane. Minor amounts of mesitylene were detected as a byproduct. Both complexes **1** and **2** exhibit high solubility in a range of polar and nonpolar solvents. The ^1^H, ^13^C and ^31^P NMR spectra of complex **2** in C_6_D_6_ at 298 K did not feature resonances corresponding to a mesityl ligand. The resonances in the ^1^H NMR spectrum showed that **2** contains an ^***i*** **Pr**^ PNNP* ligand, and is an *C*_s_-symmetric complex on the NMR time scale. In addition, two aromatic resonances were observed at δ = 7.54 ppm (coupling with H and F, multiplet) and 6.71 ppm (multiplet overlapping with a naphthyridine doublet). These two aromatic resonances both integrated in a 2:1 ratio compared to each of the naphthyridine resonances. The ^19^F NMR spectrum of **2** displays a single resonance at δ = −114.9 ppm, consistent with the presence of a single symmetrically bridging acetylide ligand on the NMR time scale. Despite repeated efforts, single crystals of complex **2** suitable for X-ray diffraction could not be obtained. However, storing a concentrated solution of complex **2** in pentane at 233 K for several days gave orange-red crystals. Interestingly, the solid-state structure revealed a neutral tricopper acetylide complex [Cu_3_(^***i*** **Pr**^ PNNP*)_2_(μ_3_-C≡C-*p*–F-C_6_H_4_)] (**Cu**_**3**_ **-acetylide**), instead of the expected dicopper acetylide complex (Scheme 3 and Figure 1).

**Scheme 3:**
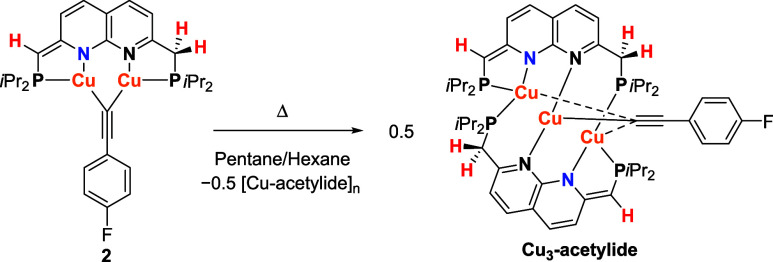
Decomposition of Complex **2** to **Cu**_**3**_ **-Acetylide**

**Figure 1:**
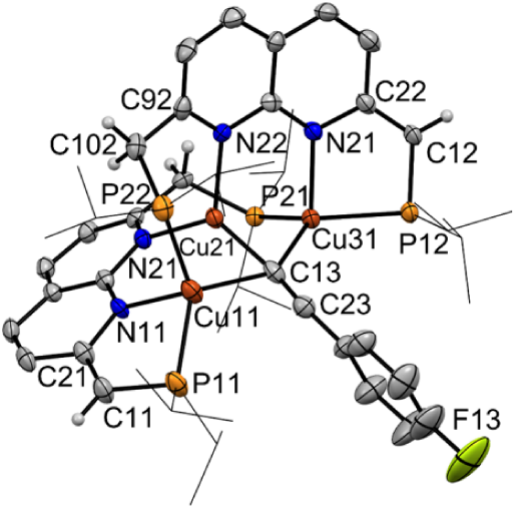
Displacement ellipsoid plots (50% probability) of **Cu**_**3**_ **-acetylide** with the -*i*Pr groups on P depicted as wireframe for clarity. Only one of the two independent, but structurally similar, molecules is depicted. Most hydrogen atoms are omitted for clarity. Selected interatomic distances (Å) and angles (°): Cu11···Cu21 2.5514(6), Cu21···Cu31 2.4918(6), Cu21–C13 1.921(4), Cu11–C13 2.342(4), Cu31–C13 2.246(3), Cu11–P11 2.2745(10), Cu11–N11 2.132(3), Cu11–P22 2.2903(12), Cu21–N21 2.003(2), Cu21–N22 2.026(3), Cu31–P21 2.2690(9), Cu31–P12 2.2671(9), Cu31–N12 2.118(3), C11–C21 1.360(4), C91–C101 1.493(4), C12–C22 1.354(5), C92–C102 1.491­(4), C13–C23 1.197(6), Cu11–Cu21–Cu31 120.75(2), C13–C23–C33 172.8(4).

This solid-state structure provides insight into the byproducts formed during both the synthesis and thermal decomposition of complex **2** (see the Supporting Information). While complex **2** is thermally stable in polar solvents, it decomposes in nonpolar solvents such as hexane/pentane upon heating to 343 K for 1 h, or even when stored at ambient temperature or 233 K over extended periods. One of the resulting species isolated under these conditions is **Cu**_**3**_ **-acetylide** (Scheme 3). We propose that this complex forms via the reaction of two equivalents of **2** with loss of an unidentified copper-acetylide fragment.^21^ Although two independent molecules of **Cu**_**3**_ **-acetylide** are found in the asymmetric unit, only the structurally nondisordered one is discussed herein. The structure features a tricopper­(I) core with a μ_3_-bridging acetylide ligand and two partially dearomatized ^***i*** **Pr**^ PNNP* ligands.

The Cu–Cu–Cu angle across the triangular core is 120.75(2)°, with the central copper atom (Cu21) lying in a plane with the proximal acetylide carbon (C13) (Figure 1). Each ^***i*** **Pr**^ PNNP* ligand exhibits one dearomatized and one aromatic naphthyridine ring, as evident from the pattern of localized single and double bonds.^19,16a^ The central copper atom (Cu21) is coordinated by two naphthyridine nitrogen donors from the neutral half of both ligands, while Cu11 and Cu31 are bound within anionic dearomatized PN pockets of one ligand and a P atom of the other ligand. In the structure, the Cu···Cu distances are comparable to those in metallic copper (2.556 Å)^22^ with Cu11···Cu21 2.5514(6) Å and Cu21···Cu31 2.4918(6) Å—all noticeably larger than that found for the 1,8-naphthyridine supported dicopper μ_2_-acetylide (Cu···Cu 2.3885(4) Å) reported by Tilley and coworkers.^9f^ C13 of the μ_3_-acetylide ligand has a short bond length with the central Cu­(I) center (Cu21–C13 1.921(4) Å) and larger bond lengths to the outer Cu­(I) centers (Cu11–C13 2.342(4) Å and Cu31–C13 2.246(3) Å). Other tri- and polynuclear copper acetylide complexes have been structurally characterized, which often contain a triangular copper motif^23^ or cubic tetramers^24^ or more complicated coordination modes.^25^ In these structures, which display similar Cu···Cu distances as **Cu**_**3**_ **-acetylide**, the acetylide ligands have been shown to bridge two, three or four^7^ copper centers and display both σ-^26^ and π-bonding.^27^ The tricopper­(I) μ_3_-acetylide core as observed in **Cu**_**3**_ **-acetylide** however, is unusual as the μ_3_-acetylide ligand is σ-coordinated to Cu21 but neither positioned well for σ- nor π-bonding to Cu11 or Cu31. σ-bonding between the lone pair on sp-hybridized C13 with Cu11 and Cu31 is expected to be limited by the large angles between the copper centers and the acetylide ligand (Cu11–C13–C23 93.5(3)°; Cu31–C13–C23 120.8(3)°). Similarly, π-bonding is expected to be weak due to the large distance between Cu11 and Cu31 and the distal carbon of the acetylide (Cu11–C23 2.696(4) Å; Cu31–C23 3.038(4) Å), which are ∼ 0.4 and 0.8 Å longer than the respective distances to the proximal acetylide carbon C13. Furthermore, the small deviation of linearity in the acetylide ligand (C13–C23–C33 172.8(4)°) and short C≡C distance (1.197(6) Å) indicate that π-bonding is only limited.^24a^ Therefore, we expect that the μ_3_-acetylide ligand is σ-bonded to Cu21 and only weakly coordinated to Cu11 and Cu31. We reason that this is likely caused by the rigid polydentate ^***i*** **Pr**^ PNNP* ligands.

### Synthesis of a Dicopper Bridging Triazolide Complex

To probe the feasibility of the bridging acetylide in **2** to undergo cycloadditions with azides, 2 equiv^28^ 1-azido-4-fluorobenzene were added to a dark red solution of complex **2** in THF at ambient temperature. After 2.5 h, full conversion of **2** was observed by NMR spectroscopy concomitant with the formation of [Cu_2_(^***i*** **Pr**^ PNNP*)­(μ-(1,4-bis­(*p*-fluorophenyl)-1,2,3-triazolide)] (**3**) (Scheme 4), as well as some 1,4-triazole products and unidentified byproducts. From this mixture complex **3** could be isolated by crystallization from Et_2_O/pentane at 233 K as an air-sensitive, orange-red crystalline solid in 24% yield.^29^

**Scheme 4:**
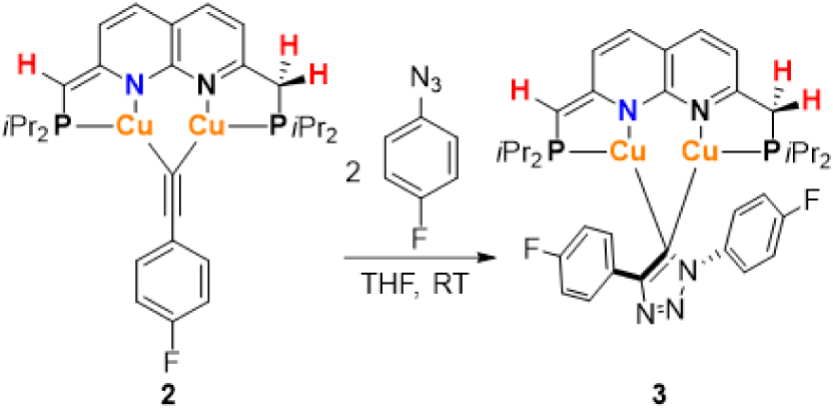
Synthesis of Dicopper Bridging Triazolide Complex **3**

The ^19^F NMR spectrum of **3** displays two resonances at δ = −116.8 and −117.0 ppm, as expected for a triazolide ligand. The ^1^H, ^13^C, and ^31^P NMR spectra of complex **3** in C_6_D_6_ at 298 K display resonances corresponding to a complex containing an ^***i*** **Pr**^ PNNP* ligand. In addition, the ^1^H NMR spectrum of complex **3** features four naphthyridine resonances and four additional aromatic resonances that integrate to two protons. An interesting ABX pattern is observed at δ = 2.34 and 2.28 ppm for the methylene protons due to coupling between protons (^2^ *J*_H,H_ = 17.2 Hz) and additional coupling to phosphorus (^2^ *J*_H,P_ = 7.9 Hz). The ^1^H­{^31^P} NMR spectrum confirms that an AB pattern is present for these resonances due to geminal coupling, showing that these protons are diastereotopic. Furthermore, **3** displays four sets of resonances for the isopropyl substituents, in contrast to complexes **1** and **2**, which only show two. These observations show that the symmetry in the naphthyridine plane, which is present in complexes **1** and **2**, is not present in **3**. This can be rationalized by the triazolide binding in a perpendicular fashion to the dicopper core, thereby creating inequivalent environments above and below the naphthyridine plane, which is consistent with the solid-state structure (see below).

Single crystals of **3** suitable for X-ray diffraction were grown from a saturated Et_2_O/pentane solution that was stored at 233 K. The solid-state structure of **3** confirmed the presence of a bridging 1,4-substituted 1,2,3-triazolide ligand bound with the carbon atom at the 5-position (C12) (Figure 2), consistent with a cycloaddition of the bridging acetylide in complex **2** and 1-azido-4-fluorobenzene. The triazolide ligand is bound orthogonally to the naphthyridine plane and is slightly closer to the copper center in the anionic binding pocket containing the methine linker (Cu1–C12 1.9940(18) Å) than to the copper center in the neutral binding pocket containing the methylene linker (Cu2–C12 2.0216(18) Å).^30^ Additionally, the bridging triazolide ligand is bend out of the naphthyridine plane, illustrated by the angle of 17.19 (8)° between the naphthyridine plane and the plane defined by Cu1, Cu2 and C12 (Figure 2, right). The short distance between the three-coordinate copper centers (Cu1–Cu2 2.3810(3) Å) is common for bridging aryl copper­(I) complexes that display electron-deficient three-center-two-electron bonding.^31^ Similar Cu–Cu distances have been observed for related reported naphthyridine-based dicopper­(I) aryl complexes^32^ and [Cu_2_(^***t*** **Bu**^ PNNP*)­(μ-Mes)].^19^ A short C11–C21 bond length (1.375(3) Å) and a longer C91–C101 bond length (1.511(3) Å) together with localized bonding of the C–C bonds in the dearomatized ring are indicative of a ^***i*** **Pr**^ PNNP* ligand, consistent with the NMR data of **3**.

**Figure 2:**
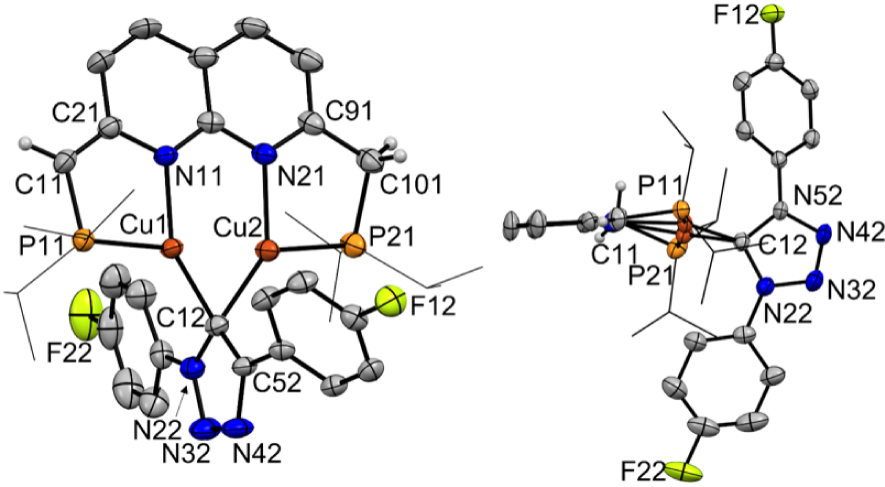
Displacement ellipsoid plots (50% probability) of the molecular structure of **3** in the crystal with the -*i*Pr groups on P depicted as wireframe for clarity. Only the major component of the orientationally disordered triazolide ring is depicted. Most hydrogen atoms and the solvent molecules are omitted for clarity. The image on the right shows the small tilt of the triazolide ligand out of the naphthyridine plane. Selected interatomic distances (Å): Cu1–Cu2 2.3810(3), Cu1–P11 2.2437(5), Cu1–N11 1.9975(15), Cu1–C12 1.9940(18), Cu2–P21 2.2265(5), Cu2–N21 2.0498(15), Cu2–C12 2.0216(18), C11–C21 1.375(3), C91–C101 1.511­(3), P11–C11 1.7873(19), P21–C101 1.845(2).

The cationic dicopper­(I) triazolide complex from the group of Tilley (Scheme 1B) shows a symmetrically bound 1,4-substituted 1,2,3-triazolide ligand,^9f^ similar to complex **3**. A notable difference is that in the cationic dicopper triazolide complex the triazolide ligand is significantly tilted out of the naphthyridine plane and shows longer bond distances in the dicopper core (Cu···Cu distance: 2.4139(5) Å); Cu–C: 2.046(2) and 2.002(2) Å; Cu–N­(naphthyridine) 2.147(2) and 2.135(2) Å) as observed for **3**. This is likely due to the more sterically demanding nature of the bis­(dipyridyl) naphthyridine ligand in comparison to the PNNP* ligand in **3**.

Both the stoichiometric cycloaddition and protodemetalation reaction were shown to be viable using the dicopper PNNP system (see above). Therefore, we examined the catalytic activity of complex **2** in the cycloaddition of 1-ethynyl-4-fluorobenzene and 1-azido-4-fluorobenzene using 5 mol % complex **2** in THF at ambient temperature for 20 h. This reaction was monitored by NMR spectroscopy and full conversion of the starting materials was observed. The sole product observed in the reaction is the expected 1,4-substituted 1,2,3-triazole, which is poorly soluble and precipitates out of solution (see experimental section for details). NMR analysis of the precipitate in DMSO-*d*_6_ confirmed that the precipitate is the 1,4-triazole, which was independently synthesized.

### Mechanistic Insights into Protodemetalation

To assess if complex **3** is a viable catalytic intermediate for the CuAAC reaction, its reactivity toward an exogenous terminal alkyne was investigated. The reaction of **3** with 1 equiv of 1-ethynyl-4-fluorobenzene in THF at ambient temperature yielded both acetylide **2** and 1,4-bis­(4-fluorophenyl)-1*H*-1,2,3-triazole according to ^1^H, ^19^F and ^31^P NMR spectroscopy (Scheme 5). Already 2 h after mixing **3** and the alkyne, 70% conversion of complex **3** was observed with the formation of **2** and the triazole and an intermediate (**4**), identified in the ^1^H, ^19^F and ^31^P NMR spectra.^33^ The ^19^F NMR spectrum in THF at 298 K displays three new resonances assigned to **4** at δ = −114.9, −119.8 and −121.0 ppm.^34^ While two resonances in the ^31^P­{^1^H} NMR spectrum at δ = 17.0 and −3.7 ppm, are suggestive of a partially dearomatized ligand backbone, the ^1^H NMR spectrum—in our experience more informative of ligand protonation state^35^—equally integrating naphthyridine doublets are found at δ = 8.41, 7.59, and 7.55 ppm (a fourth doublet is hypothesized to overlap with another resonance). The downfield shift of the naphthyridine resonances in the ^1^H NMR spectrum is indicative of the presence of an aromatized ^***i*** **Pr**^ **PNNP** ligand in **4**.^19^ In addition, two resonances at δ = 8.31 and 8.14 ppm integrating for two protons relative to the naphthyridine resonances are indicative of triazolide protons. However, other triazolide or acetylide resonances of **4** could not be clearly identified in the crowded aromatic region of the reaction mixture. Resonances corresponding to protons from a methine or methylene linker were not observed. Only traces of **4** were observed in the ^19^F NMR spectrum after 21 h when the terminal alkyne was fully consumed. These results suggest that the alkyne is involved in the formation of **4** (Scheme 5). Based on these observations, we hypothesize that **4** comprises [Cu_2_(^***i*** **Pr**^ **PNNP**)­(μ-C≡C-*p*–F-C_6_H_4_)­(μ-(1,4-bis­(*p*-fluorophenyl)-1,2,3-triazolide)] and contains both a bridging triazolide and a bridging acetylide ligand (Scheme 5).^36^ We hypothesize that **4** is formed after metal–ligand cooperative activation of the C–H bond of the alkyne with protonation of the ^***i*** **Pr**^ PNNP* ligand. Subsequently, we hypothesize that **4** cooperatively loses a 1,4-triazole by an intramolecular deprotonation of a methylenic proton to give complex **2**. A related cooperative pathway was proposed by Van der Vlugt in a mononuclear ^*t*Bu^PNP-Cu acetylide complex, which formed a 3-coordinate ^*t*Bu^PNP*Cu complex and triazole upon reaction with an organic azide.^17a^

**Scheme 5:**
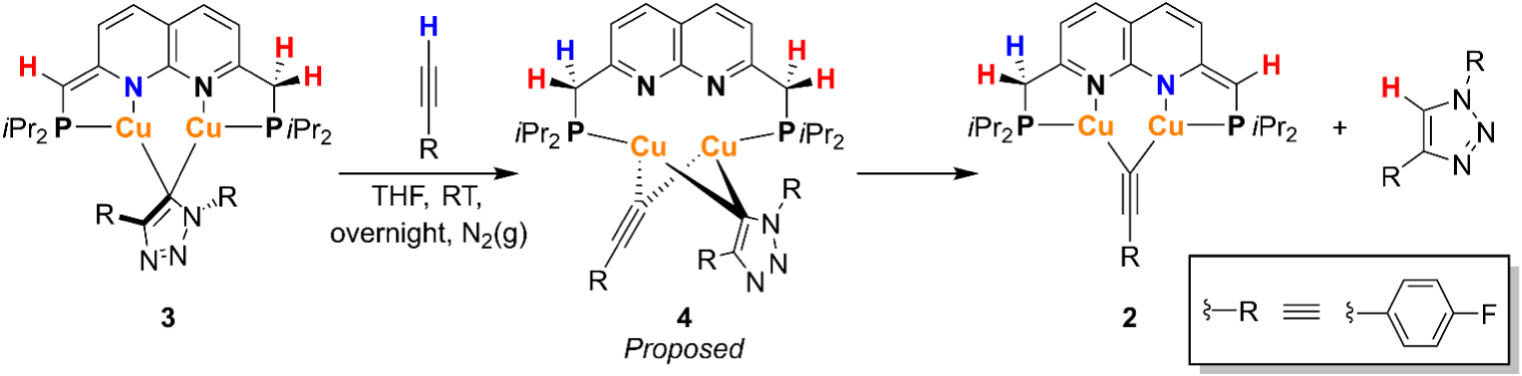
Stoichiometric Protodemetalation Reaction of **3** Using 1 Equiv of 1-Ethynyl-4-Fluorobenzene (R = *p*-Fluorophenyl)

According to experimental^9a^ and computational studies involving simple dicopper­(I) aggregates^13^ or well-defined cationic dicopper­(I) complexes,^14^ the protodemetalation step is the rate limiting step in CuAAC reactions in aprotic solvents. This step proceeds through a proton transfer from the alkyne substrate to the triazolide ligand, leading to dicopper acetylide complexes and triazole product release. The PNNP ligand in the dicopper complexes could allow for an alternative metal–ligand cooperative pathway for the protodemetalation step as proposed in Scheme 5. To gain more insight into the role of MLC in the protodemetalation step, deuterium labeling experiments were performed to assess whether the proton of the 1,4-triazole product originates from the alkyne substrate or the proton-responsive ligand. The reaction between complex **3** and 1 equiv of 1-ethynyl-4-fluorobenzene-*d*_1_ in THF at ambient temperature for 21 h yielded a triazole product that was only 6% deuterated (Scheme 5). In accordance with this, only traces of the deuterated triazole were observed in the ^2^H NMR spectrum. Moreover, partial deuteration of both the methylene/methine linkers was observed, which is indicative for the H/D exchange between these sites. This shows that the proton on the triazole originates from metal–ligand cooperative loss from the ^***i*** **Pr**^ **PNNP** ligand rather than the substrate (Scheme 5). The minor deuteration of the triazole originates from H/D exchange between the deuterated alkyne and the methine/methylene protons of the complexes, which happens at a slower, but non negligible rate (see the Supporting Information for more information).^37^

We have shown that the dicopper ^*i* **Pr**^ **PNNP** complexes display the same stoichiometric steps of the CuAAC reaction as Tilley’s related cationic dicopper complexes. In Tilley’s system, elevated temperatures and excess of reagents are required for the substitution of the arene (373 K), the cycloaddition (333 K) and protodemetalation (284 K, 20 equiv of alkyne) steps.^9f^ For the systems described herein we see that these reactions readily occur at ambient temperature or without requiring a large excess of substrate. We ascribe this difference to the more rigid ligand framework and higher-coordination number of the copper centers in Tilley’s system. In addition, the design of the PNNP ligand platform enables a metal–ligand cooperative protodemetalation which circumvents a direct alkyne-to-triazolide proton transfer, a step that is commonly rate determining in CuAAC reactions.

### Computational Analysis of Protodemetalation Pathways

The proposed metal–ligand cooperative (MLC) proton transfer pathway represents a distinct departure from the conventional concerted alkyne-to-triazole proton transfer pathway commonly considered rate-limiting in dicopper CuAAC systems.^14^ To assess the feasibility of both mechanisms in our system, we performed density functional theory (DFT) calculations at the PBE0-D3­(BJ)/6-311+G­(d,p)|SSD//6-31G­(d,p)|LANL2DZ level of theory, using the SMD solvation model with THF as the solvent (see the Supporting Information for computational details).^38^ Our analysis began from the experimentally characterized dicopper acetylide complex **2** (Figure 3). Introduction of 1-azido-4-fluorobenzene, denoted “**azide**”, to complex **2** leads to a concerted [3 + 2] cycloaddition via **2-TS** with an activation barrier of 14.0 kcal/mol, yielding the dicopper triazolide intermediate **3**. This concerted transformation is consistent with the findings of Héron and Balcells,^14^ but contrasts with the stepwise mechanism typically reported in related systems (Scheme 1).

**Figure 3:**
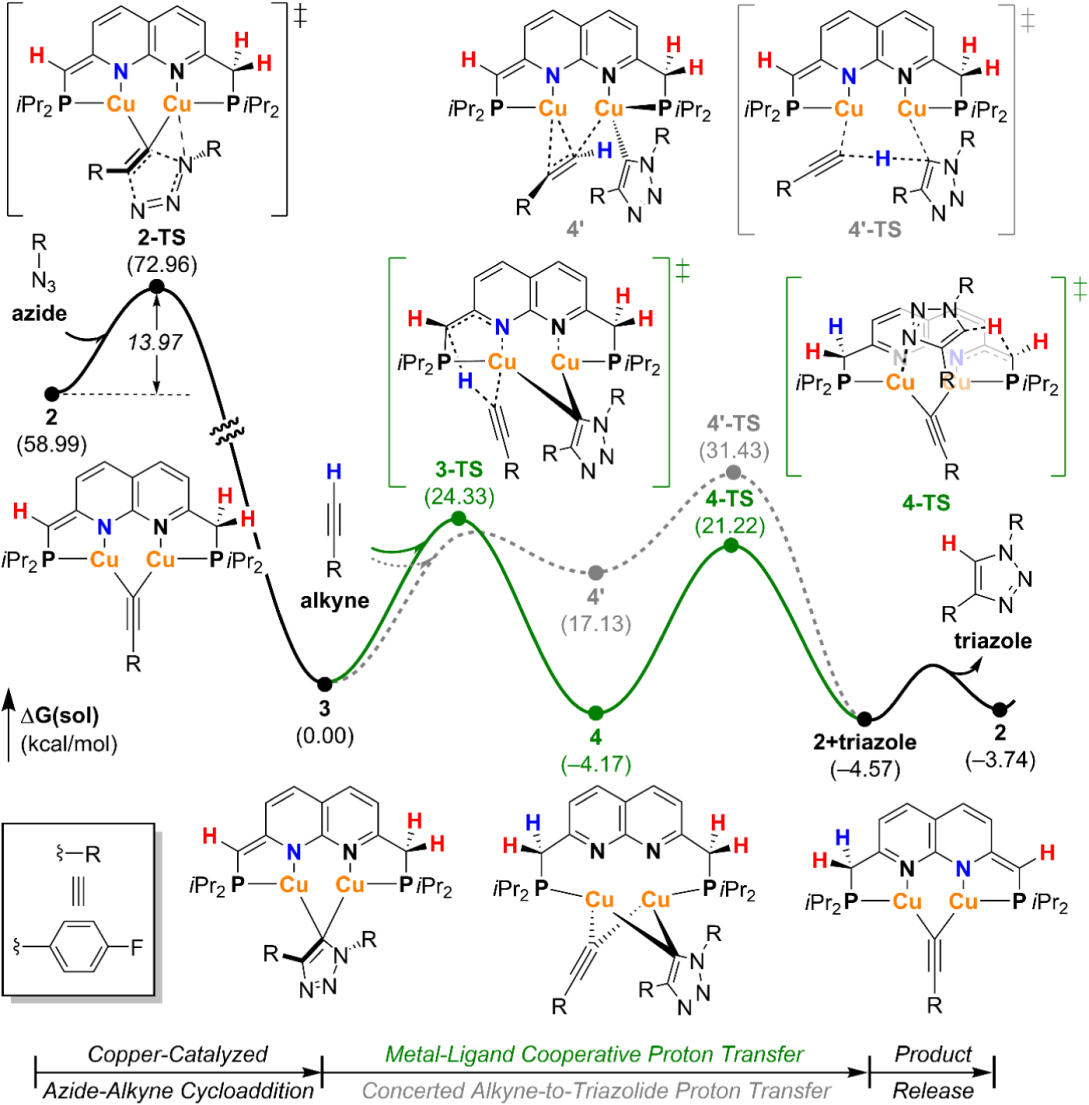
DFT-calculated energy profile of dicopper-catalyzed azide–alkyne cycloaddition reactions.

Upon addition of 1-ethynyl-4-fluorobenzene, denoted as **alkyne**, to complex **3**, two divergent pathways for protodemetalation were identified (Figure 3): (*i*) a concerted alkyne-to-triazolide proton transfer (gray, dotted line), and (*ii*) a stepwise MLC pathway (green, solid line). In the conventional route, proton transfer is accomplished by cleavage of one Cu–C bond in the triazolide-bridged core. To offset this dissociation, the system accesses a transient intermediate **4′** featuring a π interaction between the **alkyne** and the dicopper framework. This intermediate is 17.1 kcal/mol higher in Gibbs energy than complex **3**. The subsequent proton transfer transition state **4′-TS** presents a high activation barrier of 31.4 kcal/mol, rendering this pathway unlikely under the given reaction conditions. In contrast, the stepwise MLC proton transfer proceeds via two energetically more accessible transitions. The first step involves proton abstraction from the **alkyne** by a Lewis basic methine linker of the ^***i*** **Pr**^ **PNNP** ligand, forming intermediate **4**. This process occurs via transition state **3-TS** and has a barrier of 24.3 kcal/mol, which is –7.1 kcal/mol lower than the concerted pathway via **4′-TS**. Notably, the computed geometry of intermediate **4** matches well with the NMR features observed *in situ*, lending experimental support to this step. In intermediate **4**, significant elongation of the Cu–N bonds is observed (see Figure S69), likely reflecting steric repulsion between the cobound acetylide and triazolide ligands. Completion of the cycle involves proton transfer from the ligands’ methylene unit to the triazolide moiety via transition state **4-TS**, producing the triazole product and regenerating complex **2**. The associated step barrier is calculated to be 25.4 kcal/mol. Importantly, structural analysis reveals that the triazolide ligand and the relevant methylene proton occupy opposite faces of the naphthyridine plane (marked in blue in Figure 3). Therefore, only methylene protons residing on the same side of the plane as the triazolide are geometrically accessible for intramolecular proton transfer–an observation that is consistent with experimental data from isotopic labeling studies.

Together, these computational results strongly support a mechanistically distinct MLC pathway for triazole release that bypasses the high-energy direct proton transfer. The cooperative behavior of the PNNP ligand, specifically its reversible dearomatization and proton-accepting capability, is critical in facilitating this lower-energy pathway.

To further understand why the MLC proton transfer via **3-TS** is energetically favored over the direct proton transfer pathway via **4′-TS**, we examined both transition states and the shared intermediate **3** in greater detail.

In a previous study involving a cationic dicopper system lacking a proton-responsive dinucleating ligand, the triazolide-bound carbon was identified as the most nucleophilic site, thereby serving as the primary proton acceptor during the concerted transfer step.^32b^ In contrast, our system features the ^***i*** **Pr**^ PNNP* ligand, which incorporates a methine carbon within a partially dearomatized naphthyridine backbone. This methine unit exhibits significant nucleophilicity and acts as a competing Lewis basic site. Frontier molecular orbital analysis of the intermediate **3** (Figure 4A) shows that the **HOMO** is primarily located on the triazolide moiety, while **HOMO–1** is centered on the methine linker. Importantly, the energy gap between these orbitals is minimal (Δ*E* = 0.07 eV), suggesting comparable intrinsic nucleophilicity and supporting the possibility of proton transfer to either site (see Figure S70 for extended discussion).

**Figure 4:**
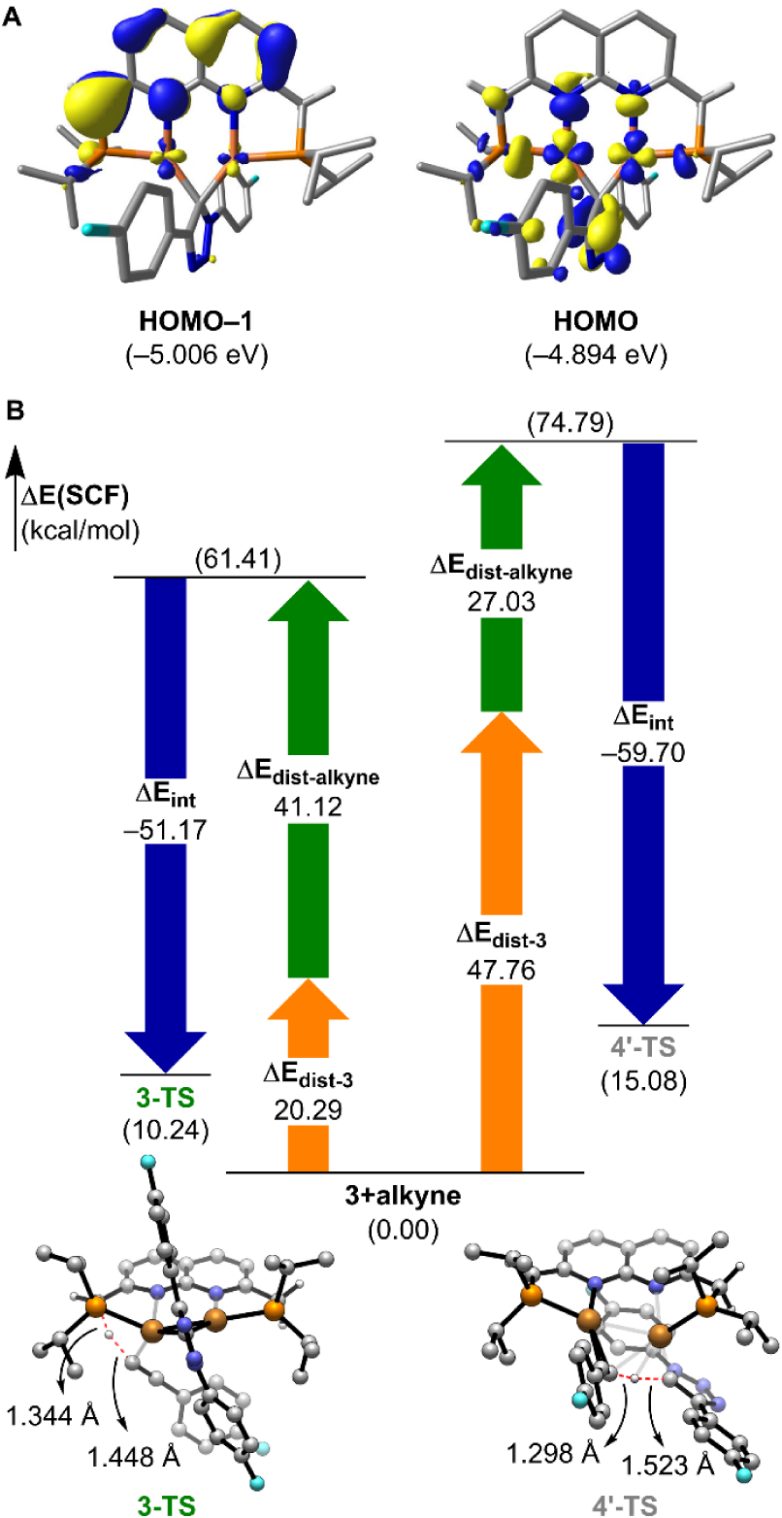
(A) Molecular orbital structures of **HOMO–1** and **HOMO** of **3** (isodensity = 0.05 au). (B) Distortion-interaction analysis and DFT-optimized structures of **3-TS** and **4′-TS**. Unnecessary hydrogen atoms are omitted for clarity.

The observed 7.1 kcal/mol Gibbs energy preference for the MLC pathway (**3-TS**) over the direct proton transfer (**4′-TS**) arises mainly from an electronic energy difference of 4.8 kcal/mol. To dissect the underlying factors, we conducted a distortion-interaction analysis based on the transition state geometries (Figure 4B).^39^ This approach partitions the total activation energy into (*i*) the distortion energy Δ*E*_dist_, reflecting the energetic cost of deforming the fragments into their TS geometries, and (*ii*) the interaction energy Δ*E*_int_ between the distorted fragments. The distortion energies differ substantially between the two pathways. The total Δ*E*_dist_ for **4′-TS** is 74.8 kcal/mol, compared to 61.4 kcal/mol for **3-TS**—a difference of 13.4 kcal/mol. This disparity is primarily attributable to a significantly higher distortion energy of intermediate **3** in **4′-TS** (Δ*E*_dist‑3_ = 47.8 kcal/mol), which is 27.5 kcal/mol greater than in **3-TS**. The elevated distortion requirement in **4′-TS** stems from the structural reorganization needed to cleave a Cu–C bond, enabling the triazolide to accept a proton directly. In contrast, protonation at the methine linker in **3-TS** occurs with minimal reorganization of the dicopper framework.

To further contextualize these findings, we examined the distortion energies associated with the alkyne fragment (Δ*E*_dist‑alkyne_). In **3-TS**, the alkyne C–H bond is elongated to 1.45 Å, corresponding to a Δ*E*_dist‑alkyne_ of 41.1 kcal/mol. By comparison, in **4′-TS**, the C–H bond length is shorter at 1.30 Å, and the associated distortion energy of 27.0 kcal/mol is lower by 14.1 kcal/mol, consistent with an earlier transition state character due to enhanced reactivity of the triazolide carbanion following Cu–C bond cleavage. The interaction energies (Δ*E*_int_) for the two transition states are broadly comparable: −51.2 kcal/mol for **3-TS** and −59.7 kcal/mol for **4′-TS**. Thus, the relatively small 8.5 kcal/mol difference in interaction energies does not compensate for the substantial difference in distortion costs. These results demonstrate that the key energetic penalty in the concerted pathway arises from the large structural reorganization required to reposition the triazolide moiety for direct proton transfer.

In summary, the MLC pathway is favored not because of intrinsically stronger orbital interactions, but because it avoids the high distortion energy associated with restructuring the dicopper-triazolide framework. This finding underscores the critical role of the PNNP ligand’s flexible and reactive methine linker in lowering the overall activation barrier through cooperative bond activation.

## Conclusions

In conclusion, we synthesized and characterized a series of well-defined dicopper complexes supported by the dinucleating, proton-responsive PNNP ligand, and demonstrated their relevance as intermediates in the copper-catalyzed azide–alkyne cycloaddition reaction. These complexes were shown to undergo stoichiometric transformations that correspond to the elementary steps of the catalytic cycle, thereby lending direct support to mechanistic proposals involving dicopper­(I) species.

A key finding of this work is the identification of an alternative pathway for the protodemetalation step, which is commonly regarded as rate-limiting in CuAAC catalysis. Rather than proceeding through a concerted alkyne-to-triazolide proton transfer, the reaction follows a two-step, metal–ligand cooperative pathway. This route is energetically favored due to the reduced structural reorganization required within the dicopper-PNNP framework. These results highlight the utility of ligand platforms that are engineered to promote both metal–metal and metal–ligand cooperativity. By enabling alternative mechanistic pathways, such cooperative ligand environments provide powerful tools for modulating the reactivity of multimetallic systems and expanding the scope of catalytic transformations.

## Supplementary Material





## Data Availability

Spectroscopic spectra, crystallographic details, and structure comparisons are found in the electronic Supporting Information. NMR data files can be obtained free of charge from YODA at: 10.24416/UU01-80P7IH.

## Notes

Cartesian coordinates of all calculated structures (XYZ)
Materials and methods, experimental procedures, X-ray crystallography details, quantum chemical calculations, and spectral data (PDF)

## Notes

Dedicated to Jarl Ivar van der Vlugt on the occasion of his 50th birthday.
