# Supplementary material for: Combined Metal–Metal and Metal–Ligand Cooperativity in Dicopper-Catalyzed Azide–Alkyne Cycloaddition Reactions
Source: Organometallics. 2025 Nov 20;44(23):2786–95. doi: 10.1021/acs.organomet.5c00387 (PMC12690585; doi:10.1021/acs.organomet.5c00387)
Supplement: Supplementary file 2 [file om5c00387_si_002.pdf]

Combined Metal-Metal and Metal-Ligand Cooperativity in Dicopper Catalyzed Azide-Alkyne Cycloaddition Reactions

SUPPORTING INFORMATION

Cody B. van Beek,[a] Hyoju Choi,[c,d] Marije L. A. Hilberts,[a] Marijn M. Lammertink,[a] Bohyun Park,[d] Martin Lutz,[b] Mu-Hyun Baik,[d,c,*] and Daniël L. J. Broere[a,*]

[a]Organic Chemistry and Catalysis, Institute for Sustainable Chemistry and Catalysis, Faculty of Science, Utrecht University, Universiteitsweg 99, 3584 CG, Utrecht, The Netherlands.

[b]Structural Biochemistry, Bijvoet Centre for Biomolecular Research, Faculty of Science, Utrecht University, Universiteitsweg 99, 3584 CG, Utrecht, The Netherlands.

[c]Department of Chemistry, Korea Advanced Institute of Science and Technology (KAIST), Daejeon 34141, Korea.

[d]Center for Catalytic Hydrocarbon Functionalizations, Institute for Basic Science (IBS), Daejeon 34141, Korea.

Email:

Daniël L. J. Broere: d.l.j.broere@uu.nl

Mu-Hyun Baik: mbaik2805@kaist.ac.kr

Table of Contents

Table of Contents S2

General considerations S3

Syntheses and characterization S4

[Cu_2(^{iPr}PNNP*)(μ-Mes)] (Mes = mesityl) (**1**) S4

[Cu_2(^{iPr}PNNP*)(μ-C≡C-*p*-F-C_6H_4)] (**2**) S10

[Cu_2(^{iPr}PNNP*)(μ-(1,4-bis(*p*-fluorophenyl)-1,2,3-triazolide)] (**3**) S16

1,4-bis(4-fluorophenyl)-1H-1,2,3-triazole and 1,5-bis(4-fluorophenyl)-1H-1,2,3-triazole S22

Formation of a tricopper(I) acetylide complex S32

H/D scrambling in dicopper acetylide complex **2** S36

Protodemetalation of complex **3** with (deuterated) alkyne S38

Crude NMR spectra of the synthesis of complex **3** S43

CuAAC reaction using 5 mol% complex **2** S44

Crystallographic details S45

Computational details S46

Supplementary calculations S47

Energy components for optimized structures S51

Vibrational frequencies of optimized structures S52

References S69

General considerations

All manipulations were performed under N_2 atmosphere using standard Schlenk techniques or inside of a N_2-filled M. Braun glovebox using dry solvents and reagents, unless stated otherwise. The ambient temperature inside the glovebox is typically between 298 and 303 K. Glassware was dried at 403 K in an oven or with a heat gun under a dynamic vacuum, unless noted otherwise. Hexane, Et_2O, and toluene were collected from an M. Braun MB-SPS-800 solvent purification system and degassed and stored over 4 Å molecular sieves. THF was dried over benzophenone/sodium, distilled and degassed, subsequently followed by storage over 4 Å molecular sieves. Benzene (Scharlab, >99%) and pentane (technical, VWR chemicals) were degassed, then dried and stored over 4 Å molecular sieves. All non-deuterated solvents were degassed by bubbling N_2(g) through the solvent for at least 30 min. The solvents (1.0 mL) were tested with a standard purple solution of sodium benzophenone ketyl in THF to confirm effective oxygen and water removal (max 1-2 drops for most solvents, max 4 drops for THF and Et_2O). All solvents were checked for water content by Karl-Fischer titration and should be well below 5 ppm for all solvents. Deuterated solvents were obtained from Cambridge Isotope Laboratories except for THF-d_8, which was obtained from ABCR, degassed by the standard freeze-pump-thaw procedure and stored over 4 Å molecular sieves. All commercial reagents were used as received and were obtained from Sigma Aldrich, Acros and Strem. ^{iPr}PNNP,[1] 1-azido-4-fluorobenzene,[2] H-BArF$_{24}$·2 Et_2O (Brookhart's acid) [3] and 1-ethynyl-4-fluorobenzene-d_1 [4] were prepared according to literature procedures. NMR data was recorded on an Agilent MRF 400 equipped with a OneNMR probe and Optima Tune system or a Varian VNMR-S-400 equipped with an AutoX DB probe and ProTune system or on a Jeol JNM-ECZL G 400 MHz (9.4 T), fitted with an autotunable ROYALPROBE HFX and a 90 G/cm gradient amplifier at 298 K. All chemical shifts are reported in the standard δ notation of parts per million, referenced to the residual solvent peak. All resonances in ^{1}H NMR and ^{13}C NMR spectra were referenced to residual solvent peaks[5] (^{1}H NMR: 7.26 for $CDCl_3$, 7.16 for C_6D_6, 3.58 for THF-d_8, ^{13}C NMR: 77.16 for $CDCl_3$, 128.06 for C_6D_6, 67.57 for THF-d_8). The resonances in the ^{19}F NMR, and ^{31}P NMR spectra are referenced using the absolute reference method from a correctly referenced ^{1}H NMR spectrum of the same sample using the MNova software. The assignment of peaks is based on relative integration, chemical shift, and 2D NMR analysis (COSY, HMQC and HMBC experiments). For ^{1}H NMR spectra in non-deuterated solvents, solvent suppression is used (Presat).

IR-data was recorded on a PerkinElmer SpectrumTwo Infrared Spectrophotometer equipped with an ATR-probe. IR-analysis of air-sensitive compounds was performed by dropcasting a THF solution onto the ATR crystal, which was covered by a continuous N_2(g) flow. Elemental analysis was performed by MEDAC Ltd. based in the United Kingdom.

Syntheses and characterization

$[Cu_2(^{iPr}PNNP^*)(\mu\text{-Mes})]$ (Mes = mesityl) (**1**)

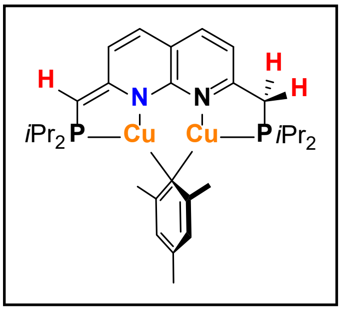

A solution of ^{iPr}**PNNP** (391.2 mg, 1.0 mmol) in THF (7 mL) was added dropwise over the course of 6 minutes to a suspension of CuMes (365.5 mg, 2.0 mmol) in THF (8 mL), instantly giving a red solution. After 3.5 h, the mixture was concentrated under a dynamic vacuum. The resulting oil was stripped with pentane (5 mL) to give a red solid that was dried under a dynamic vacuum. The solid obtained was extracted with pentane (16 mL) and filtered through a glass frit. The total volume was concentrated to ~10 mL and the solution was placed in the freezer at 243 K. After 4 days, crystals had formed, the supernatant was pipetted off and the solids were washed with cold pentane (2 mL). The red crystals were dried under a dynamic vacuum resulting in complex **1** (442 mg, 70%).

^{1}H NMR (400 MHz, C_6D_6, 298 K): δ = 6.96 (s, 2H, Mes-*H*), 6.76 (d, $^3J_{H,H}$ = 7.1 Hz, 1H, napy-*H*), 6.50 (d, $^3J_{H,H}$ = 9.1 Hz, 1H, napy-*H*), 6.43 (dd, $^3J_{H,H}$ = 9.1 Hz, $^3J_{H,P}$ = 1.8 Hz, 1H, napy-*H*), 5.98 (d, $^3J_{H,H}$ = 7.1 Hz, 1H, napy-*H*), 4.14 (d, $^2J_{H,P}$ = 2.7 Hz, 1H, methine-*H*), 2.88 (s, 6H, Mes *o*-C*H*$_3$), 2.39 (d, $^2J_{H,P}$ = 7.6 Hz, 2H, methylene-*H*), 2.30 (s, 3H, Mes *p*-C*H*$_3$), 1.73 (dhept, $^3J_{H,H}$ = 7.0 Hz, $^2J_{H,P}$ = 7.0 Hz, 2H, -C*H*(CH$_3$)$_2$), 1.31 (dhept, $^3J_{H,H}$ = 7.0 Hz, $^2J_{H,P}$ = 7.0 Hz, 2H, -C*H*(CH$_3$)$_2$), 1.15 - 1.08 (m, 6H, -CH(C*H*$_3$)$_2$), 1.08 - 1.02 (m, 6H, -CH(C*H*$_3$)$_2$), 0.71 (dd, $^2J_{H,P}$ = 16.9 Hz, $^3J_{H,H}$ = 7.0 Hz, 6H, -CH(C*H*$_3$)$_2$), 0.62 (dd, $^2J_{H,P}$ = 13.0 Hz, $^3J_{H,H}$ = 7.0 Hz, 6H, -CH(C*H*$_3$)$_2$) ppm.

^{13}C{^{1}H} NMR (101 MHz, C_6D_6, 298 K): δ = 166.4 (d, $^2J_{C,P}$ = 16.8 Hz), 156.3 (d, $^2J_{C,P}$ = 4.6 Hz), 154.9, 149.0 (t, $^3J_{C,P}$ = 2.9 Hz), 144.3 - 143.7 (m), 135.0 (t, $^5J_{C,P}$ = 2.5 Hz), 133.8 (d, $^3J_{C,P}$ = 2.3 Hz), 129.1 (d, $^3J_{C,P}$ = 2.3 Hz), 126.4 (d, $^4J_{C,P}$ = 8.8 Hz), 124.6 (t, $^4J_{C,P}$ = 1.7 Hz), 118.5 (t, $^5J_{C,P}$ = 2.3 Hz), 109.6 (d, $^4J_{C,P}$ = 3.1 Hz), 79.7 (d, $^1J_{C,P}$ = 37.4 Hz), 31.0 (d, $^1J_{C,P}$ = 14.9 Hz), 30.0, 23.4 (d, $^2J_{C,P}$ = 16.8 Hz), 22.5 (d, $^2J_{C,P}$ = 10.3 Hz), 21.7, 20.2 (d, $^2J_{C,P}$ = 12.6 Hz), 19.3 (d, $^2J_{C,P}$ = 11.4 Hz), 18.7 (d, $^2J_{C,P}$ = 2.7 Hz), 18.2 (d, $^2J_{C,P}$ = 3.1 Hz) ppm.

^{31}P{^{1}H} NMR (162 MHz, C_6D_6, 298 K): δ = 6.6 (s, 1P), –7.0 (s, 1P) ppm.

ATR-IR (film, N_2 flow): ν = 3007 (w), 2951 (m), 2923 (m), 2890 (m), 2864 (m), 1616 (m), 1537 (m), 1493 (m), 1456 (w), 1447 (w), 1417 (s), 1381 (w), 1363 (w), 1320 (m), 1131 (w), 824 (w) cm^{-1}.

Anal. Calcd. For $C_{31}H_{46}Cu_2N_2P_2$: C, 58.57; H, 7.29; N, 4.41. Found: C, 58.72; H, 7.22; N, 4.36.

Note: While the *t*Bu analogue of this compound, previously reported,[6] is already pure after the pentane extraction, **1** is obtained with mesitylene and 10-15% of an unidentified byproduct (possibly CuMes), see Figure S10. Therefore, the crystallization step from pentane is needed to obtain clean batches of **1**.

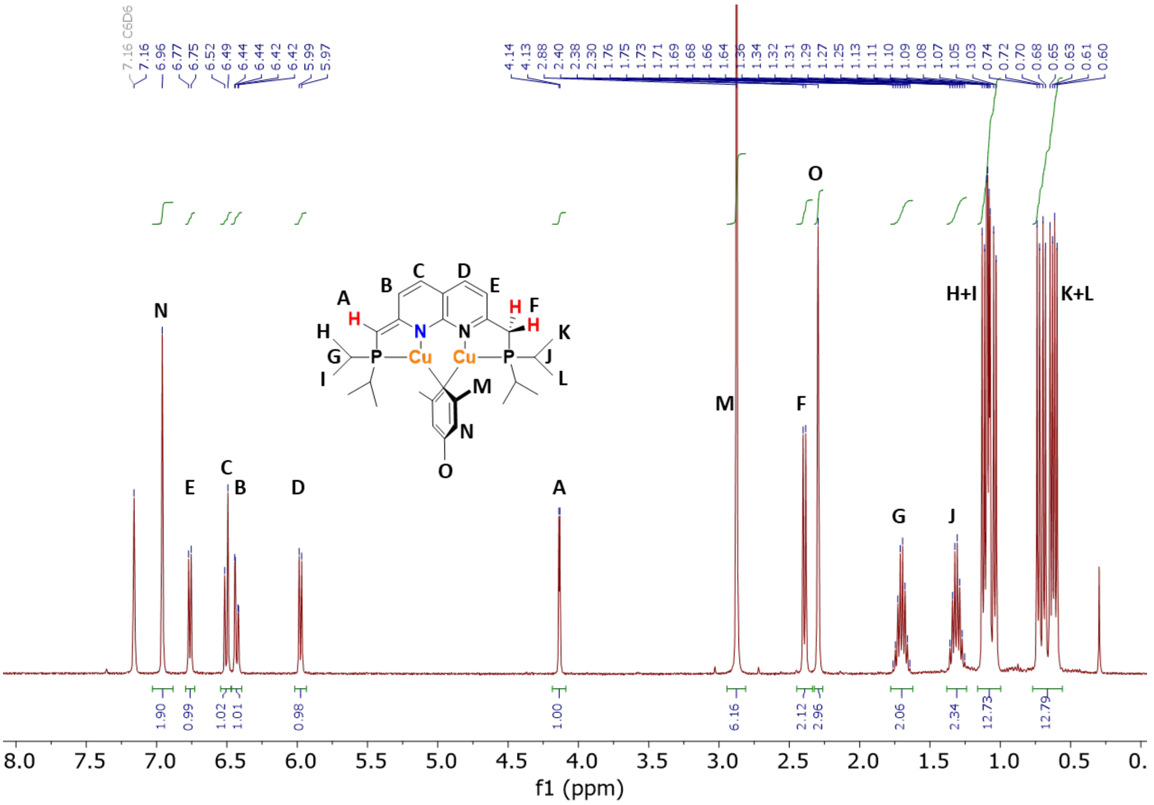

Figure S1: The ^{1}H NMR spectrum of complex **1** in C_6D_6 at 298 K.

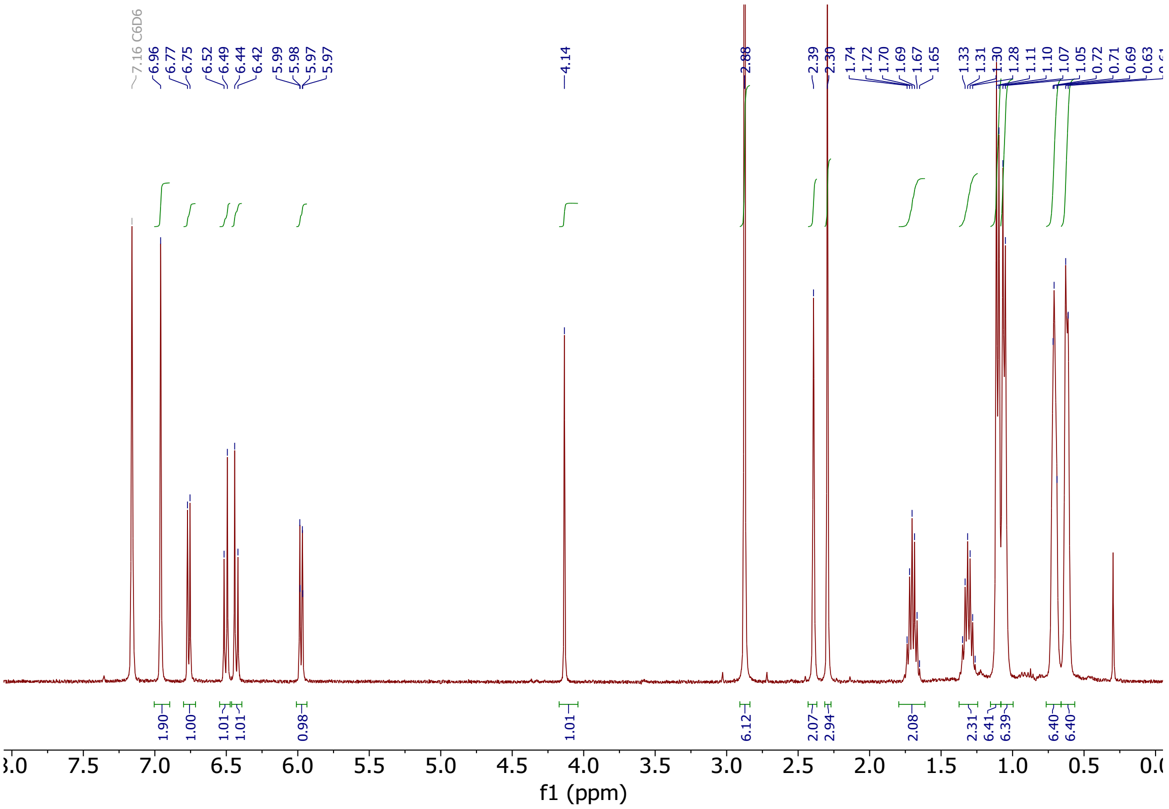

Figure S2: The $^{1}H\{^{31}P\}$ NMR spectrum of complex **1** in C_6D_6 at 298 K.

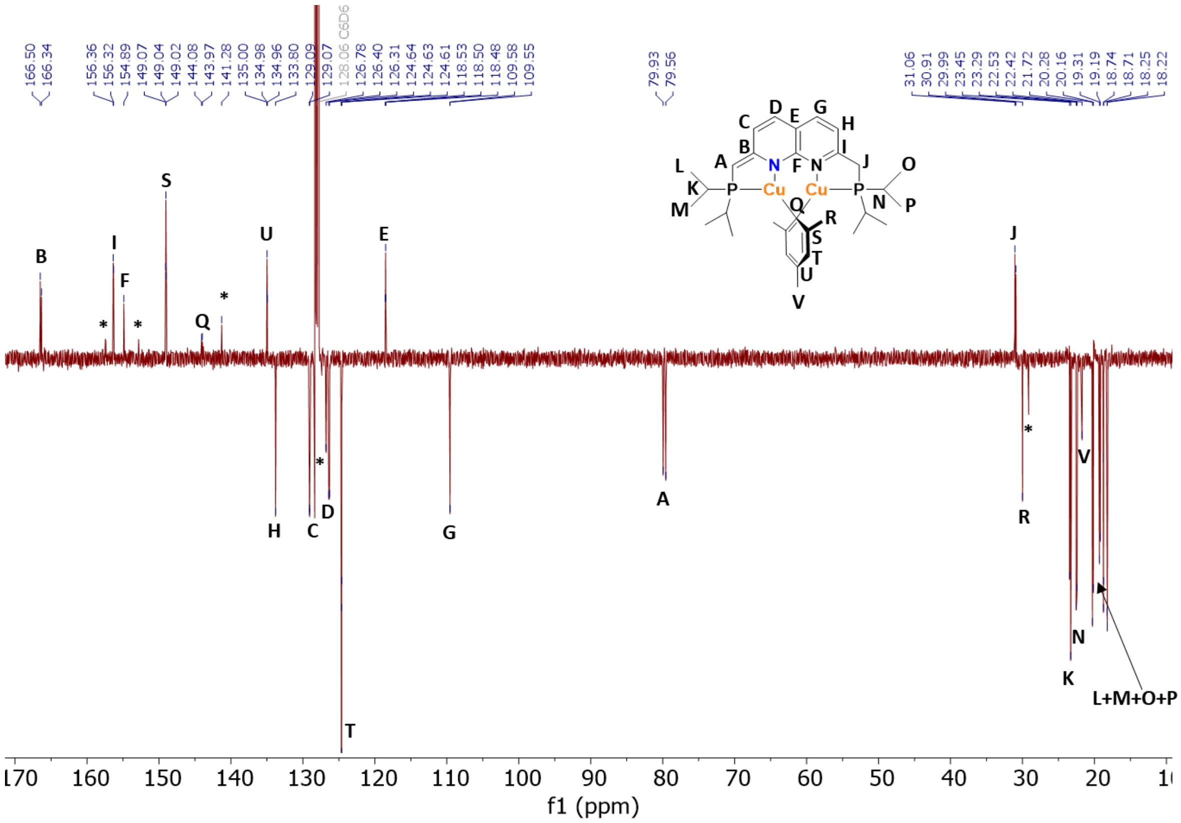

Figure S3: The $^{13}C\{^{1}H\}$ APT NMR spectrum of complex **1** in C_6D_6 at 298 K.

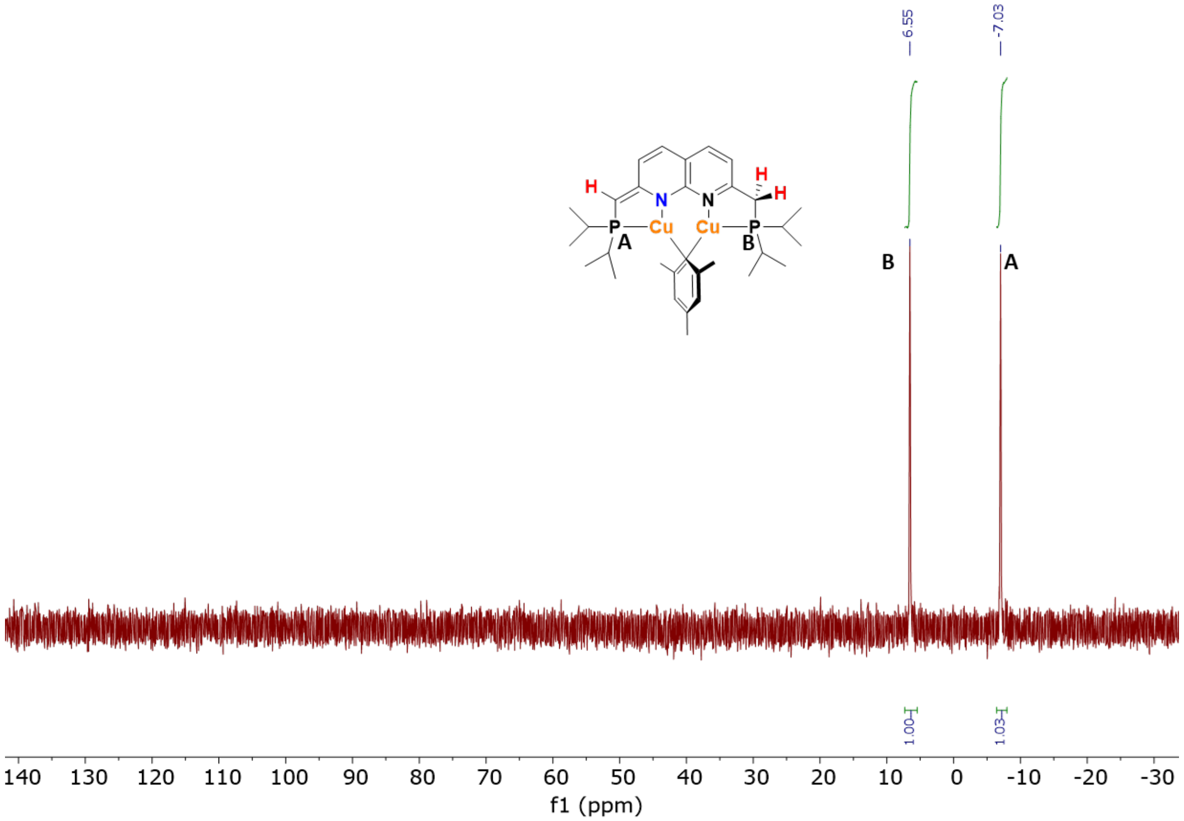

Figure S4: The $^{31}P\{^{1}H\}$ NMR spectrum of complex **1** in C_6D_6 at 298 K.

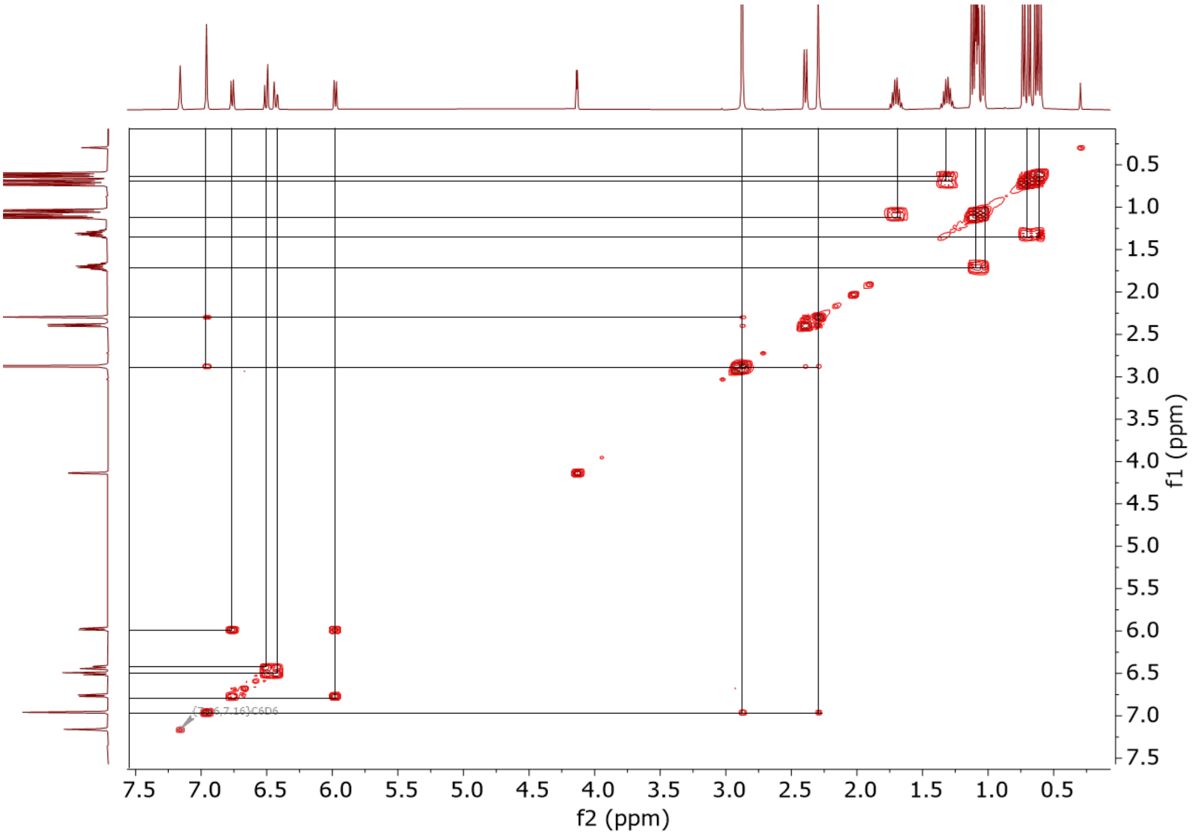

Figure S5: The ^{1}H-^{1}H COSY NMR spectrum of complex **1** in C_6D_6 at 298 K.

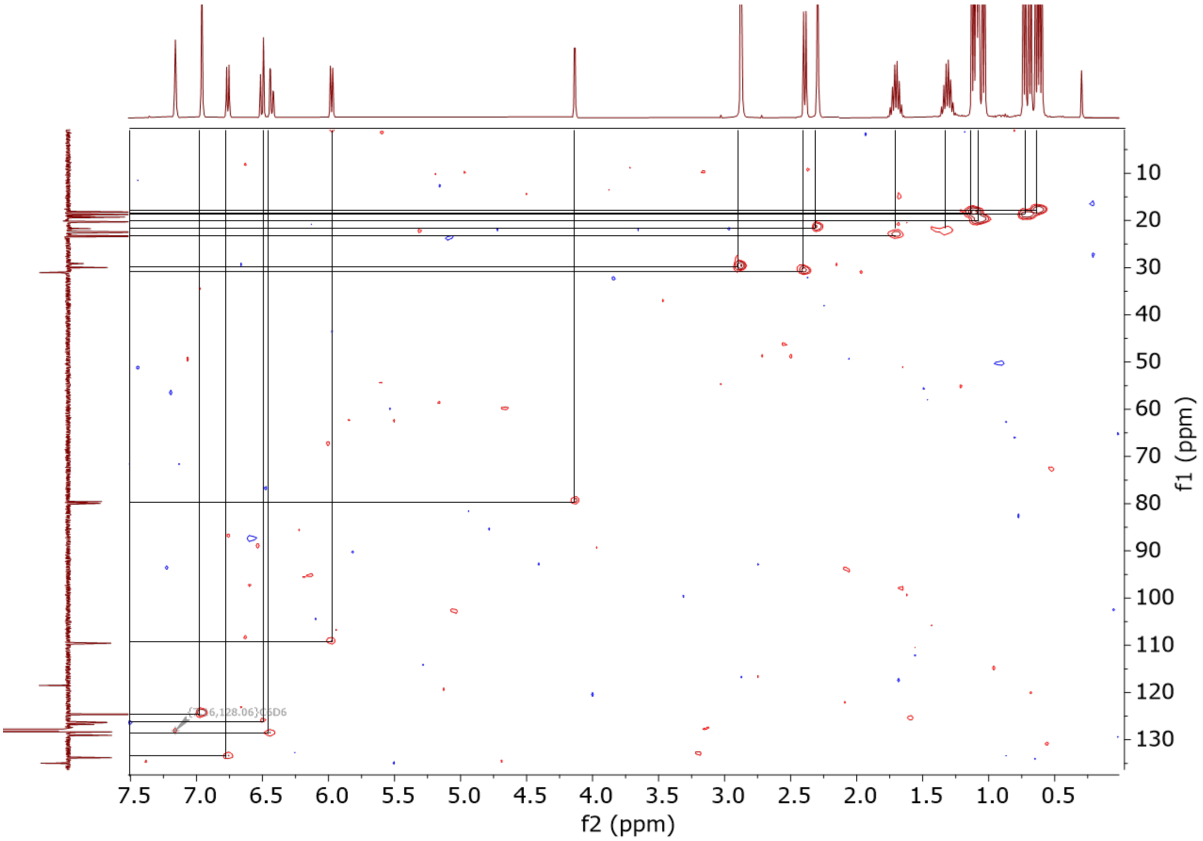

Figure S6: The ^{1}H-^{13}C ASAPHMQC NMR spectrum of complex **1** in C_6D_6 at 298 K.

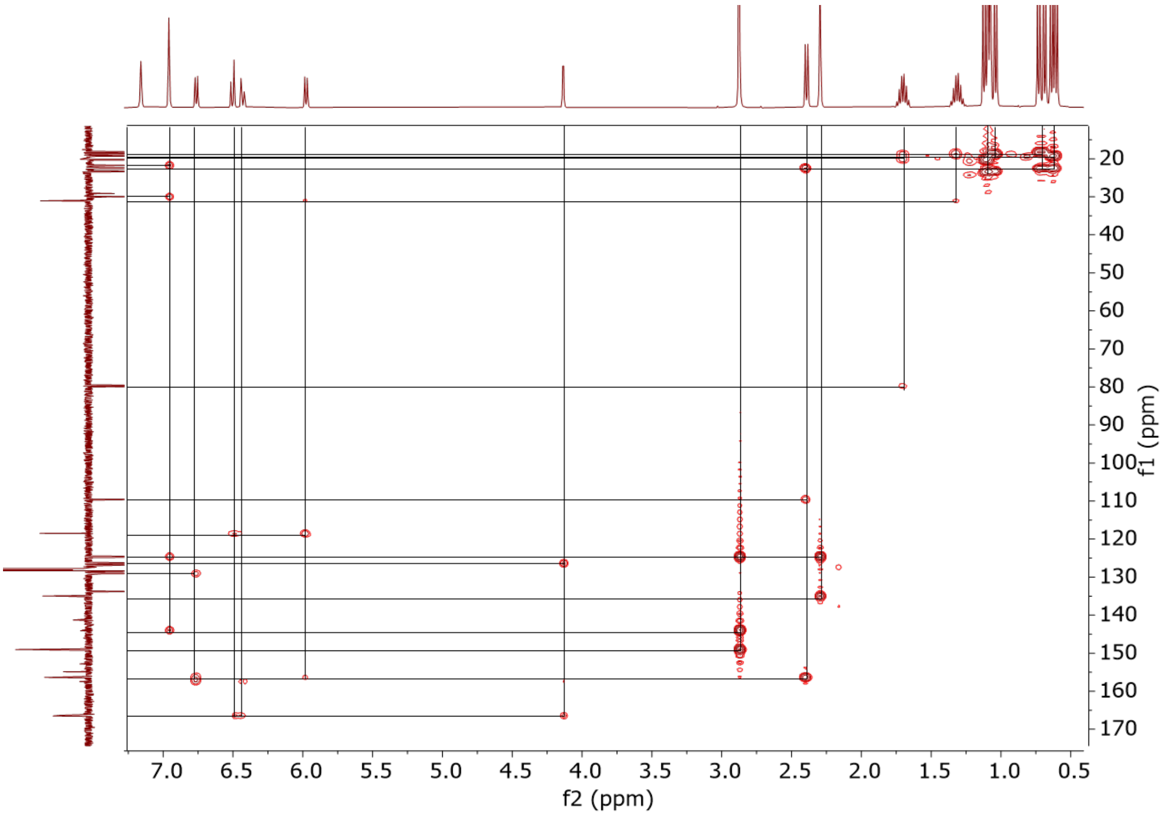

Figure S7: The ^{1}H-^{13}C gHMBCAD NMR spectrum of complex **1** in C_6D_6 at 298 K.

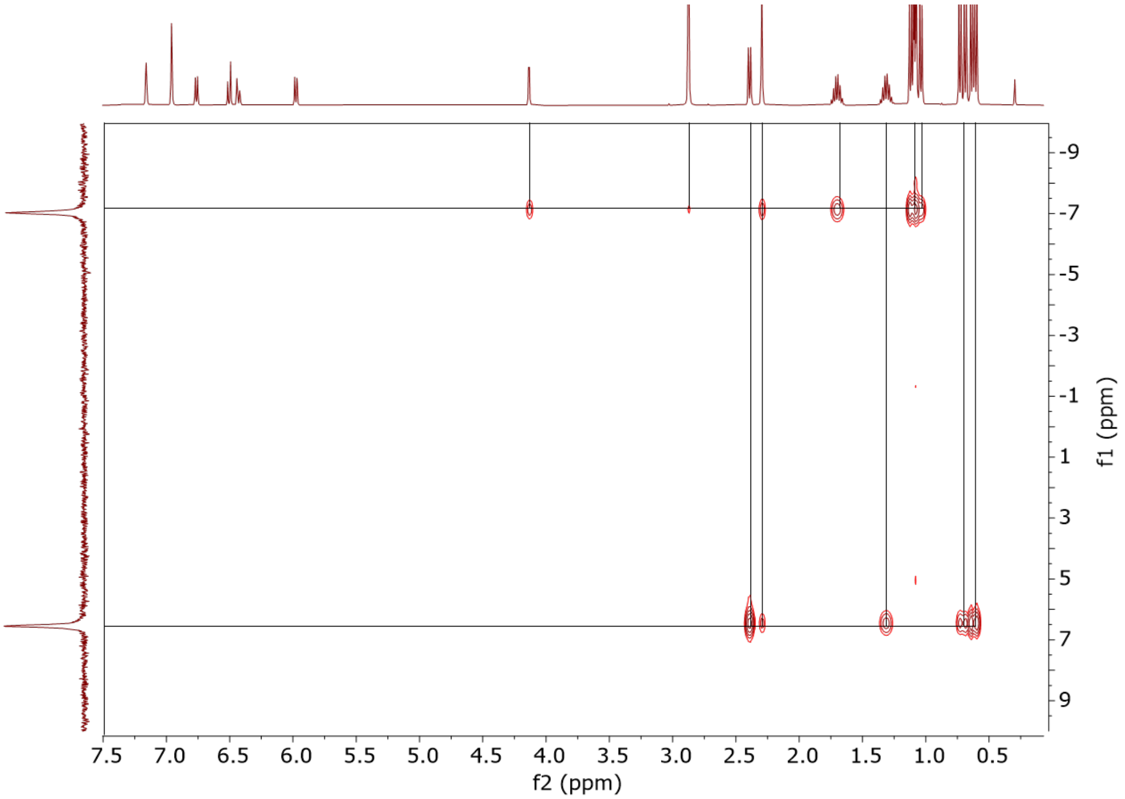

Figure S8: The ^{1}H-^{31}P HMBC NMR spectrum of complex **1** in C_6D_6 at 298 K.

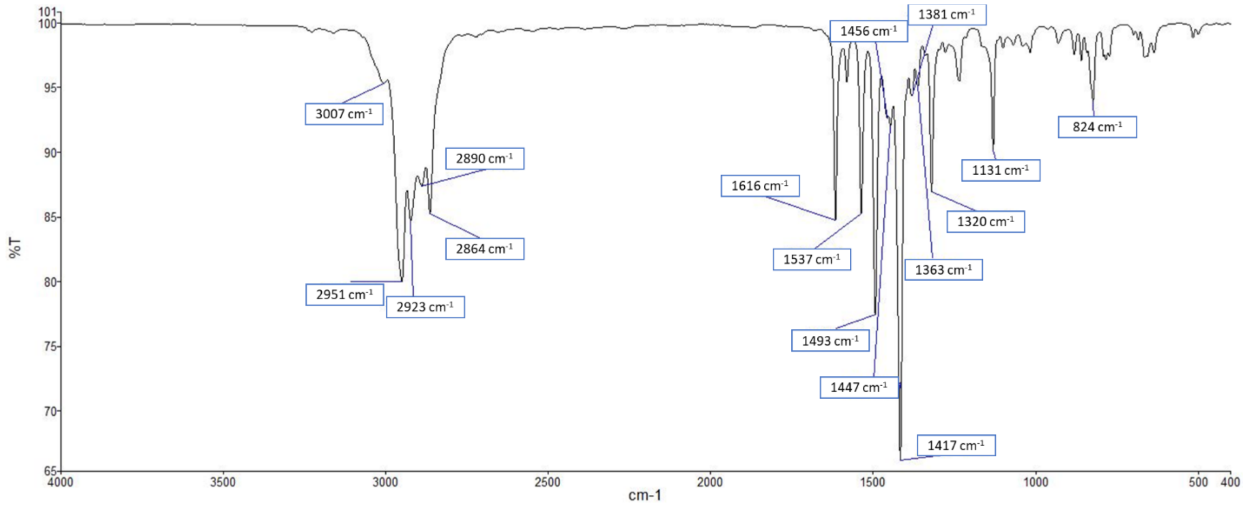

Figure S9: The ATR-IR spectrum of complex **1** measured as a film under N_2 flow.

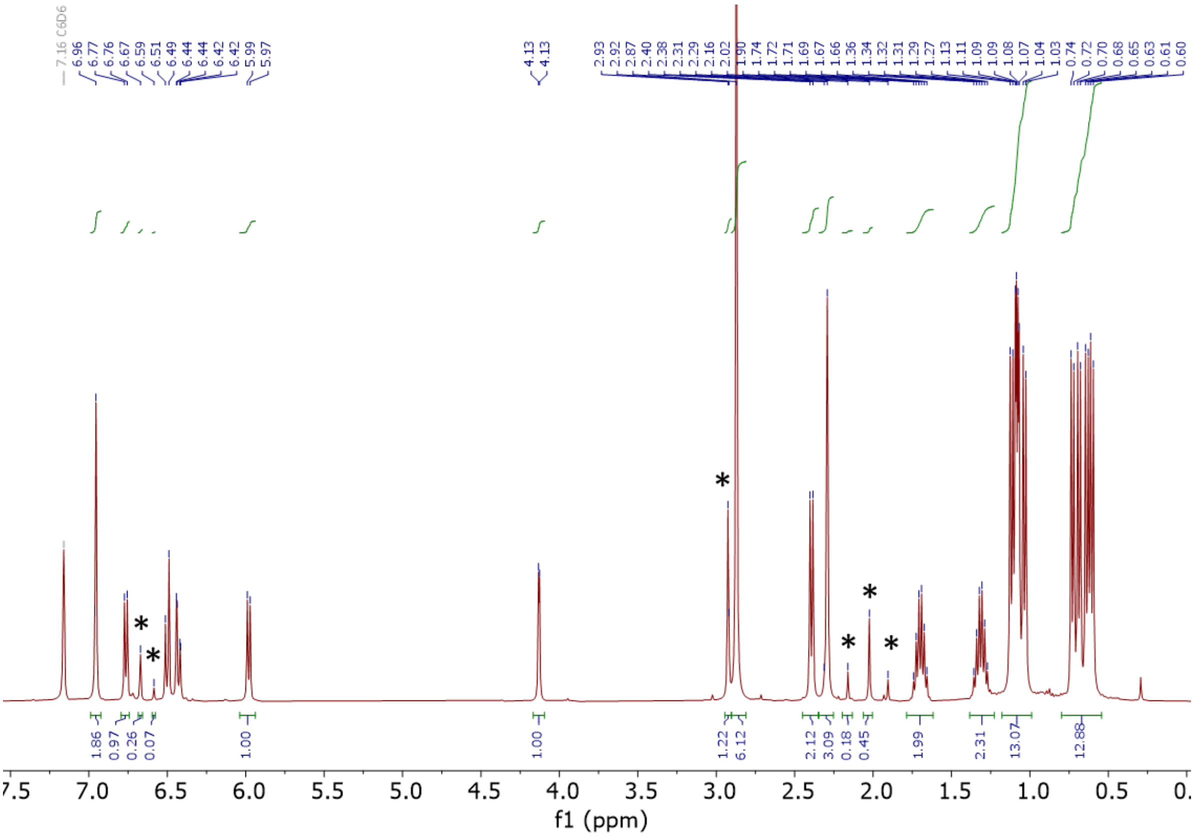

Figure S10: The ^{1}H NMR spectrum of impure complex **1** in C_6D_6 at 298 K obtained after pentane extraction from the crude mixture. The impurities (mesitylene and possibly copper mesityl) are assigned with *.

$[Cu_2(^{iPr}PNNP^*)(\mu\text{-}C{\equiv}C\text{-}p\text{-}F\text{-}C_6H_4)]$ (2)

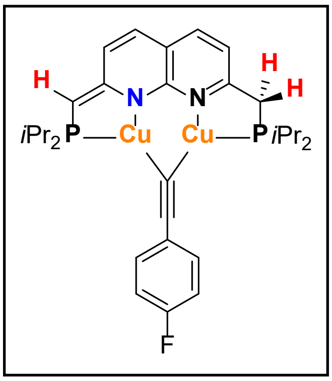

1-ethynyl-4-fluorobenzene (37.2 µL, 39.1 mg, 0.32 mmol, 2 equiv) was added by to a stirred red solution of complex **1** in THF (4 mL) (102.4 mg, 0.16 mmol) at ambient temperature. After 19 h, the red reaction mixture was concentrated under a dynamic vacuum. Pentane (12 mL) was added to the resulting red oil and the obtained mixture was vigorously stirred for 90 min. The resulting red mixture was filtered and the mixture was concentrated under a dynamic vacuum resulting in complex **2**, which was isolated as a red oil (89 mg, 89%) that still contains around 0.1 equiv of mesitylene as observed by ^{1}H NMR analysis.

^{1}H NMR (400 MHz, C_6D_6, 298 K): δ = 7.65 - 7.43 (m, 2H, *m*-phenyl), 6.79 - 6.63 (m, 3H, overlapping napy-H and o-phenyl), 6.52 (d, $^3J_{H,H}$ = 9.0 Hz, 1H, napy-*H*), 6.41 (dd, $^3J_{H,H}$ = 9.0 Hz, $^4J_{H,P}$ = 1.9 Hz, 1H, napy-*H*), 5.95 (d, $^3J_{H,H}$ = 7.1 Hz, 1H, napy-*H*), 4.16 (d, $^2J_{H,P}$ = 2.5 Hz, 1H, methine-*H*), 2.40 (d, $^2J_{H,P}$ = 7.0 Hz, 2H, methylene-*H*), 1.79 (dhept, $^3J_{H,H}$ = 6.9 Hz, $^2J_{H,P}$ = 6.9 Hz, 2H, -C*H*(CH$_3$)$_2$), 1.46 (dhept, $^3J_{H,H}$ = 7.0 Hz, $^2J_{H,P}$ = 7.0 Hz, 2H, -C*H*(CH$_3$)$_2$), 1.08 (m, 6H, two overlapping dd -CH(C*H*$_3$)$_2$), 1.04 (m, 6H, two overlapping dd -CH(C*H*$_3$)$_2$), 0.79 (dd, $^3J_{H,P}$ = 16.6 Hz, $^3J_{H,H}$ = 7.0 Hz, 6H, CH(C*H*$_3$)$_2$), 0.65 (dd, $^3J_{H,P}$ = 13.2 Hz, $^3J_{H,H}$ = 7.0 Hz, 6H, CH(C*H*$_3$)$_2$) ppm.

^{13}C{^{1}H} NMR (101 MHz, C_6D_6, 298 K): δ = 167.9 (d, $^2J_{C,P}$ = 17.2 Hz), 162.8, 160.4, 158.0, 157.1, 134.1, 133.1 (d, $^2J_{C,F}$ = 7.6 Hz), 129.2 (d, $^3J_{C,P}$ = 2.7 Hz), 127.0 (d, $^4J_{C,P}$ = 9.9 Hz), 118.9, 115.2 (d, $^3J_{C,F}$ = 21.7 Hz), 110.4, 80.2 (d, $^1J_{C,P}$ = 37.8 Hz), 31.5 (d, $^1J_{C,P}$ = 14.1 Hz), 23.6 (d, $^1J_{C,P}$ = 18.3 Hz), 22.5 (d, $^1J_{C,P}$ = 11.4 Hz), 19.8 (d, $^2J_{C,P}$ = 11.1 Hz), 19.3 (d, $^2J_{C,P}$ = 9.5 Hz), 18.4 (d, $^2J_{C,P}$ = 2.3 Hz), 18.2 (d, $^2J_{C,P}$ = 3.1 Hz) ppm.

^{19}F NMR (376 MHz, C_6D_6, 298 K): δ = –114.9 (tt, $^3J_{F,H}$ = 14.8 Hz, $^4J_{F,H}$ = 5.7 Hz) ppm.

^{31}P{^{1}H} NMR (162 MHz, C_6D_6, 298 K): δ = 10.6 (s, 1P), –9.2 (s, 1P) ppm.

ATR-IR (film, N_2 flow): v = 2952 (m). 2925 (m), 2887 (m), 2865 (m), 2045 (w), 1619 (m), 1539 (m), 1497 (s), 1459 (w), 1411 (s), 1381 (w), 1363 (w), 1321 (m), 1226 (m), 1152 (w), 1134 (m), 832 (m) cm^{-1}.

Anal. Calcd. For $C_{30}H_{39}Cu_2FN_2P_2$: C, 56.68; H, 6.18; N, 4.41. Found C, 58.39; H, 6.48; N, 4.12. The found values are slightly high in C and H and lower in N, which we ascribe to the sample containing residual mesitylene and pentane. Inclusion of these impurities in approximate ratios as observed in the ^{1}H NMR spectrum (0.1 eq mesitylene and 0.3 equiv pentane, Figure S11) leads to a satisfactory elemental analysis.

Note 1: Using impure batches of complex **1** for the synthesis of complex **2**, leads to a proposed tricopper byproduct, which is very challenging to separate from complex **2**. Therefore, spectroscopically clean batches of complex **1** should be used for the synthesis of **2**. The NMR spectra of a mixture containing **2** and the byproduct **Cu_3-acetylide** are shown in Figures S48 – S51. Note 2: Complex **2** is thermally unstable in the solid phase in a matter of weeks. Therefore, complex **2** should be stored as a solid in the freezer at 243 K.

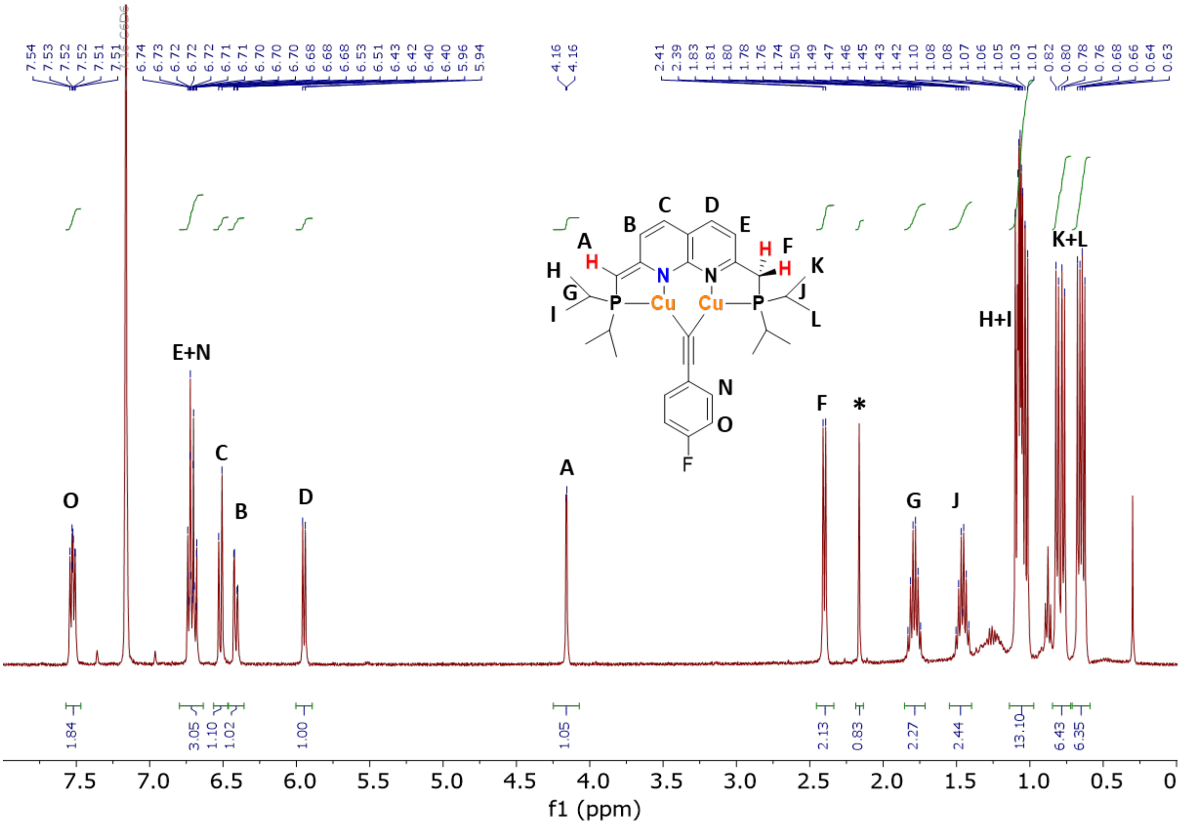

Figure S11 The ^{1}H NMR spectrum of complex **2** in C_6D_6 at 298 K. The resonance marked with * is assigned to residual mesitylene.

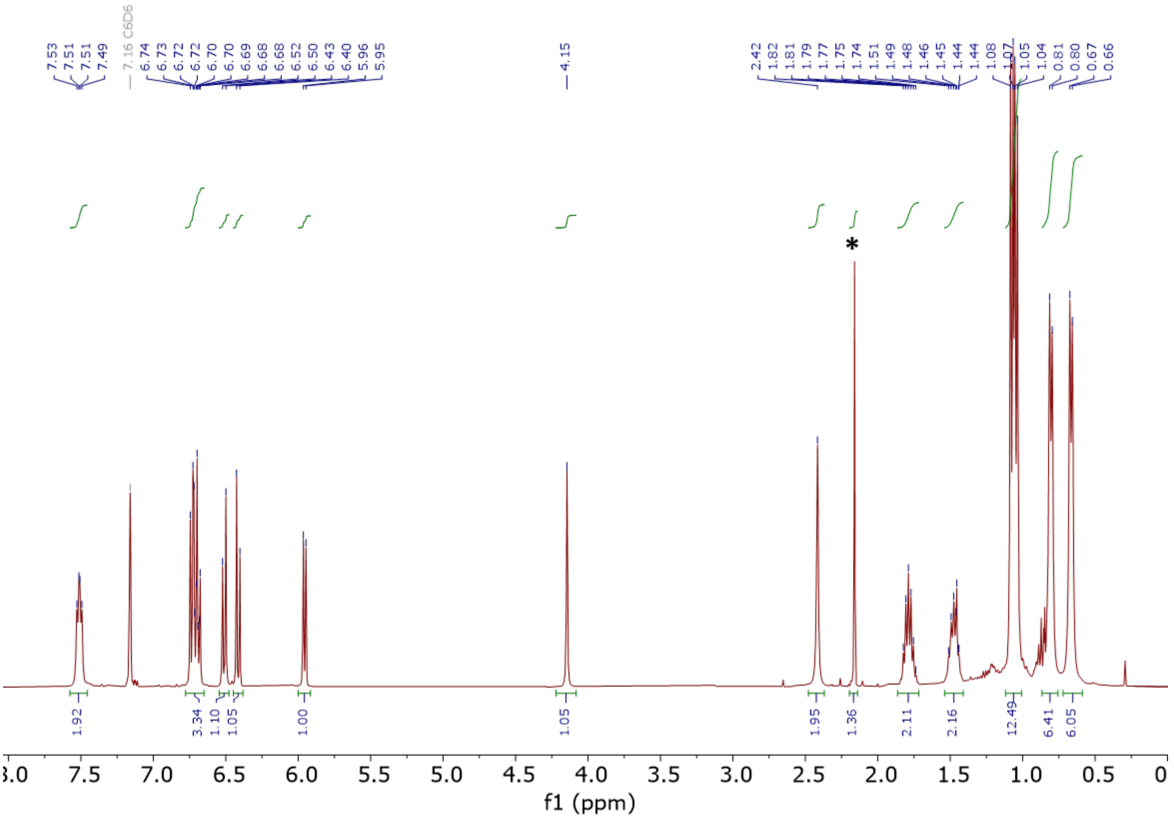

Figure S12: The $^{1}H\{^{31}P\}$ NMR spectrum of complex **2** in C_6D_6 at 298 K. The resonance marked with * is assigned to residual mesitylene.

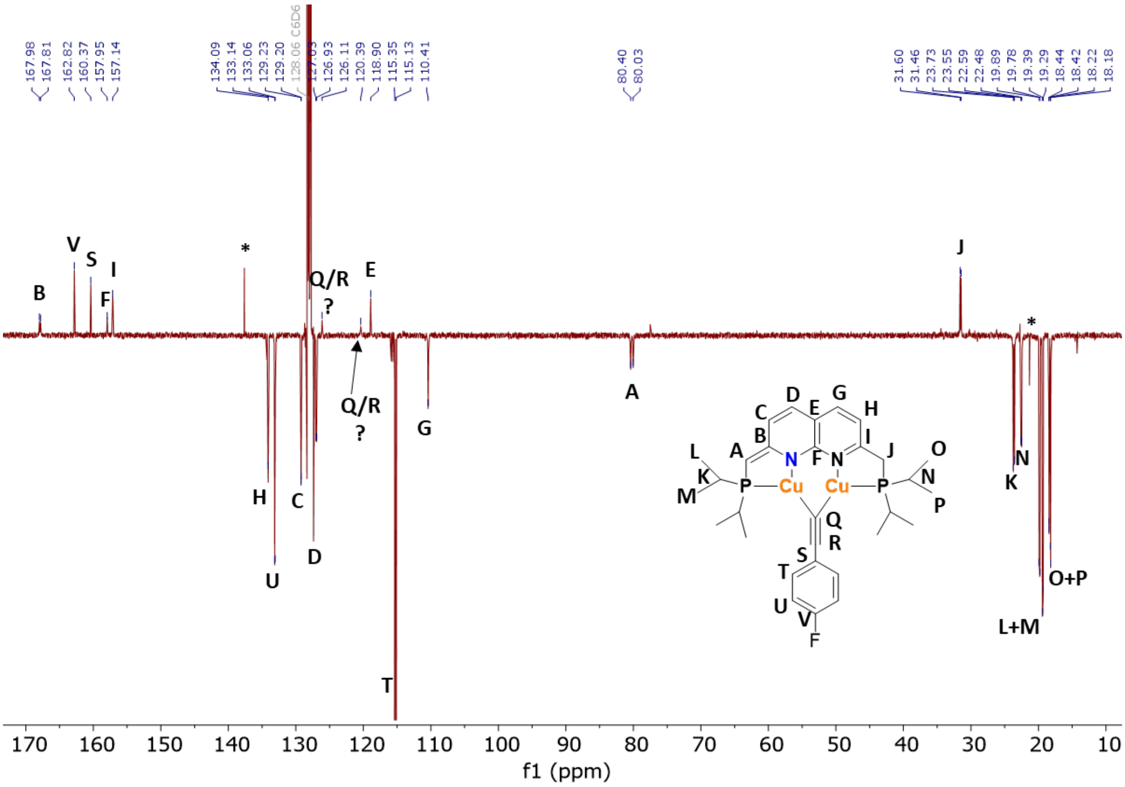

Figure S13: The $^{13}C\{^1H\}$ APT NMR spectrum of complex **2** in C_6D_6 at 298 K. The resonances marked with * are assigned to residual mesitylene.

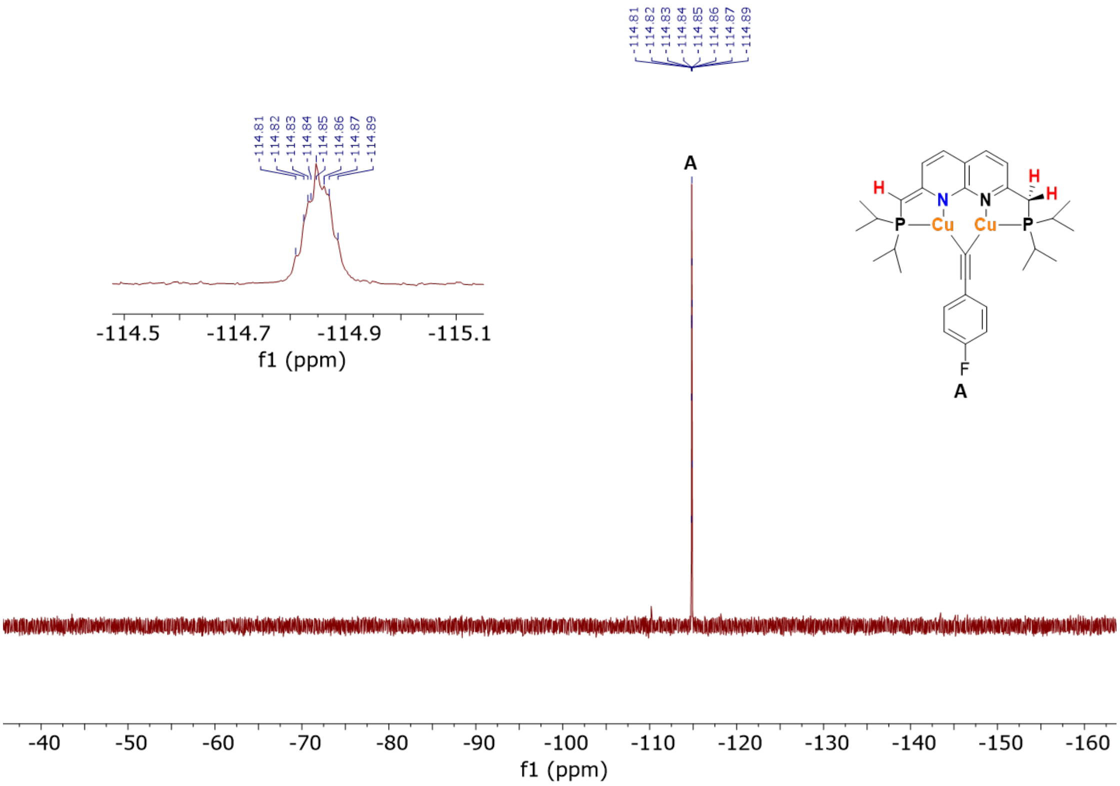

Figure S14: The ^{19}F NMR spectrum of complex **2** in C_6D_6 at 298 K.

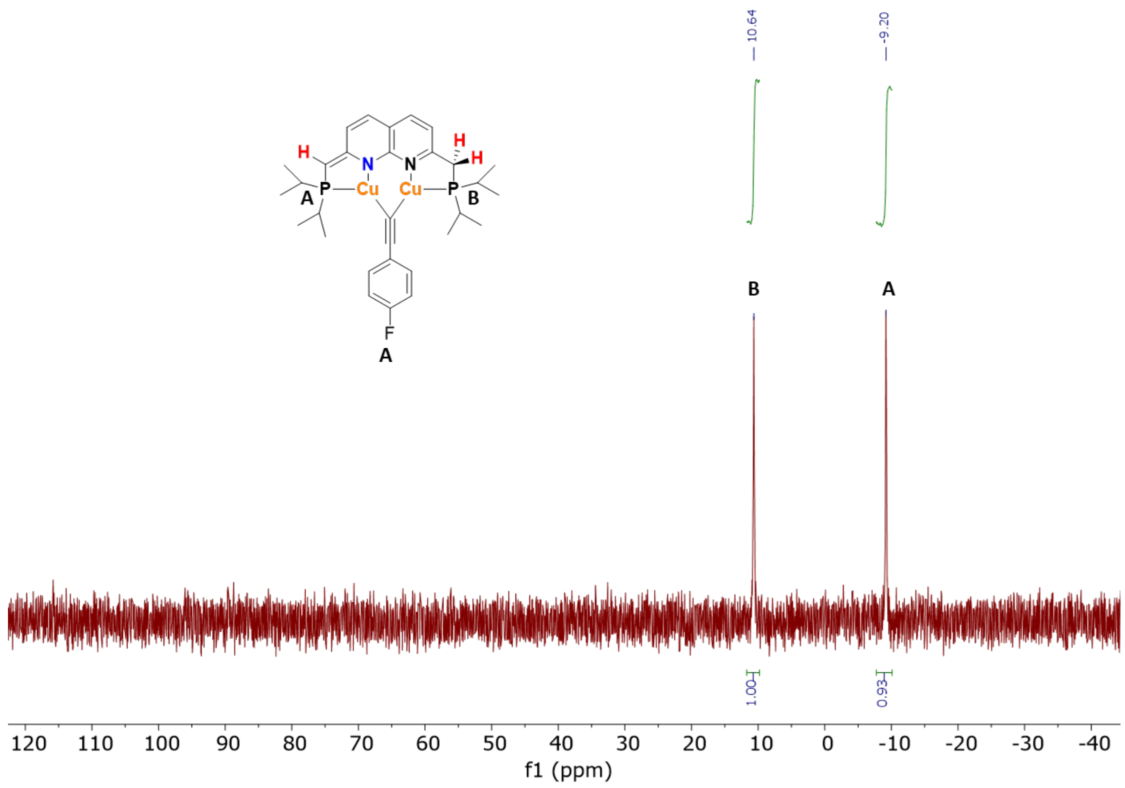

Figure S15: The $^{31}P\{^{1}H\}$ NMR spectrum of complex **2** in C_6D_6 at 298 K.

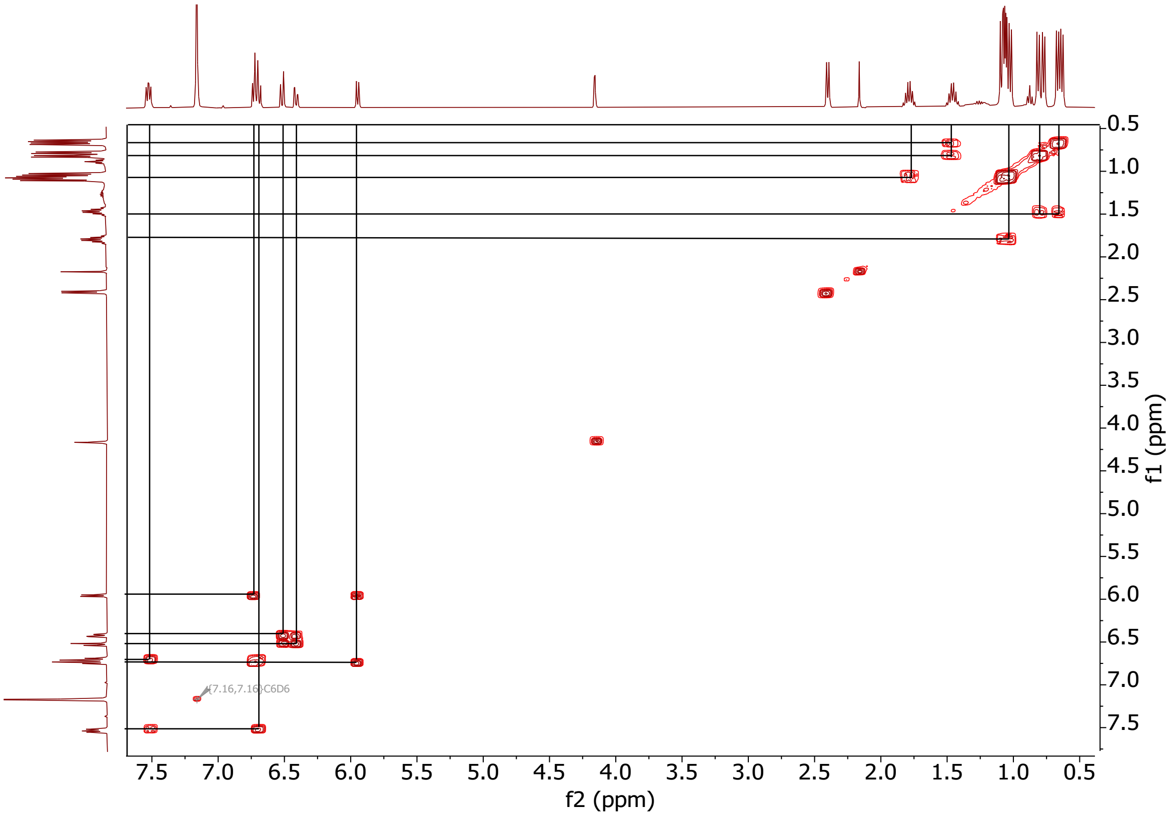

Figure S16: The ^{1}H-^{1}H gCOSY NMR spectrum of complex **2** in C_6D_6 at 298 K.

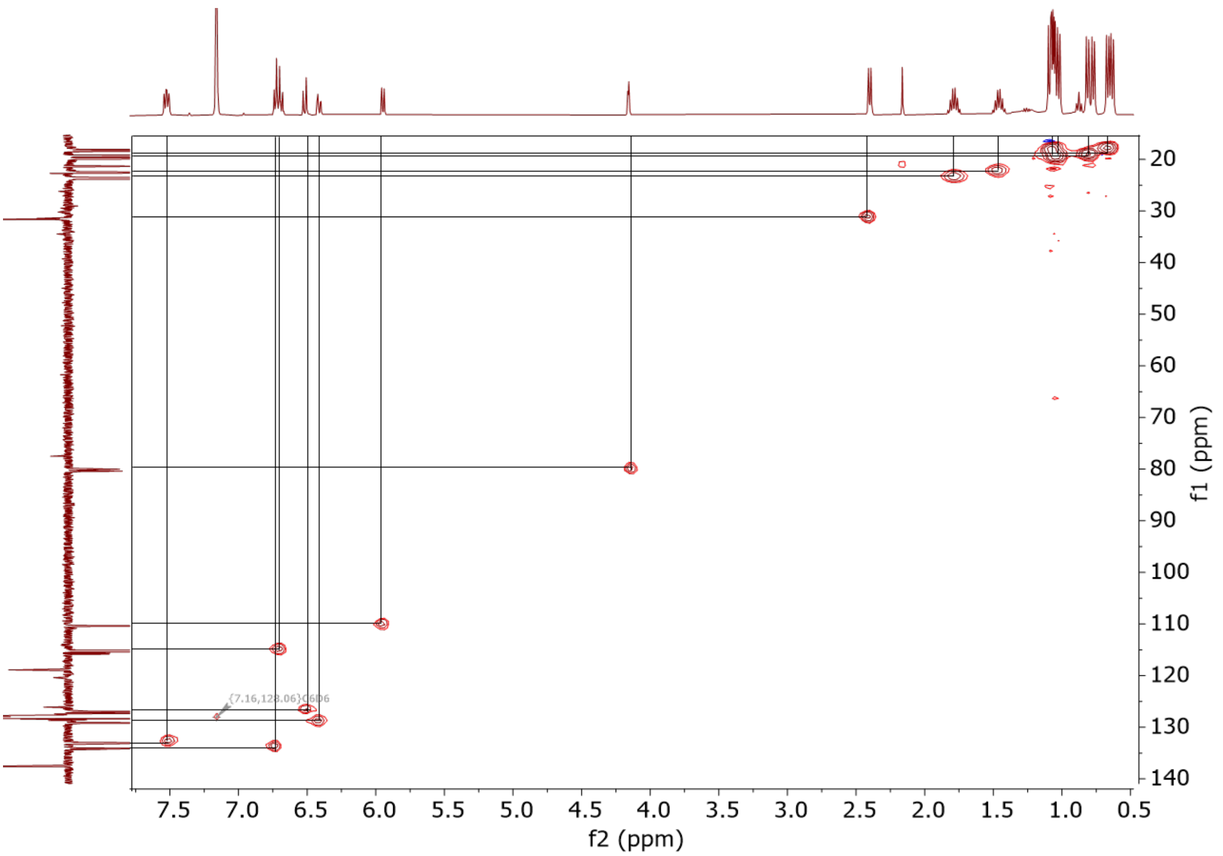

Figure S17: The ^{1}H-^{13}C ASAPHMQC NMR spectrum of complex **2** in C_6D_6 at 298 K.

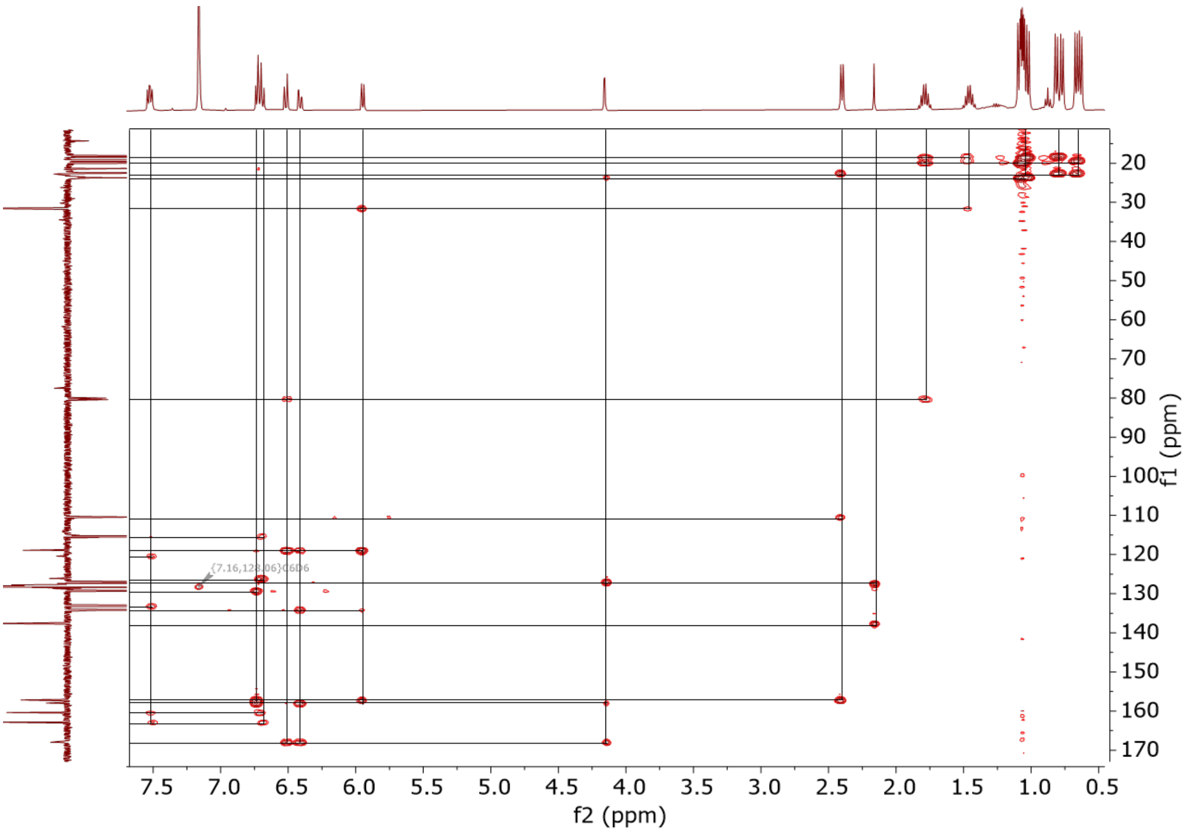

Figure S18: The ^{1}H-^{13}C gHMBCAD NMR spectrum of complex **2** in C_6D_6 at 298 K.

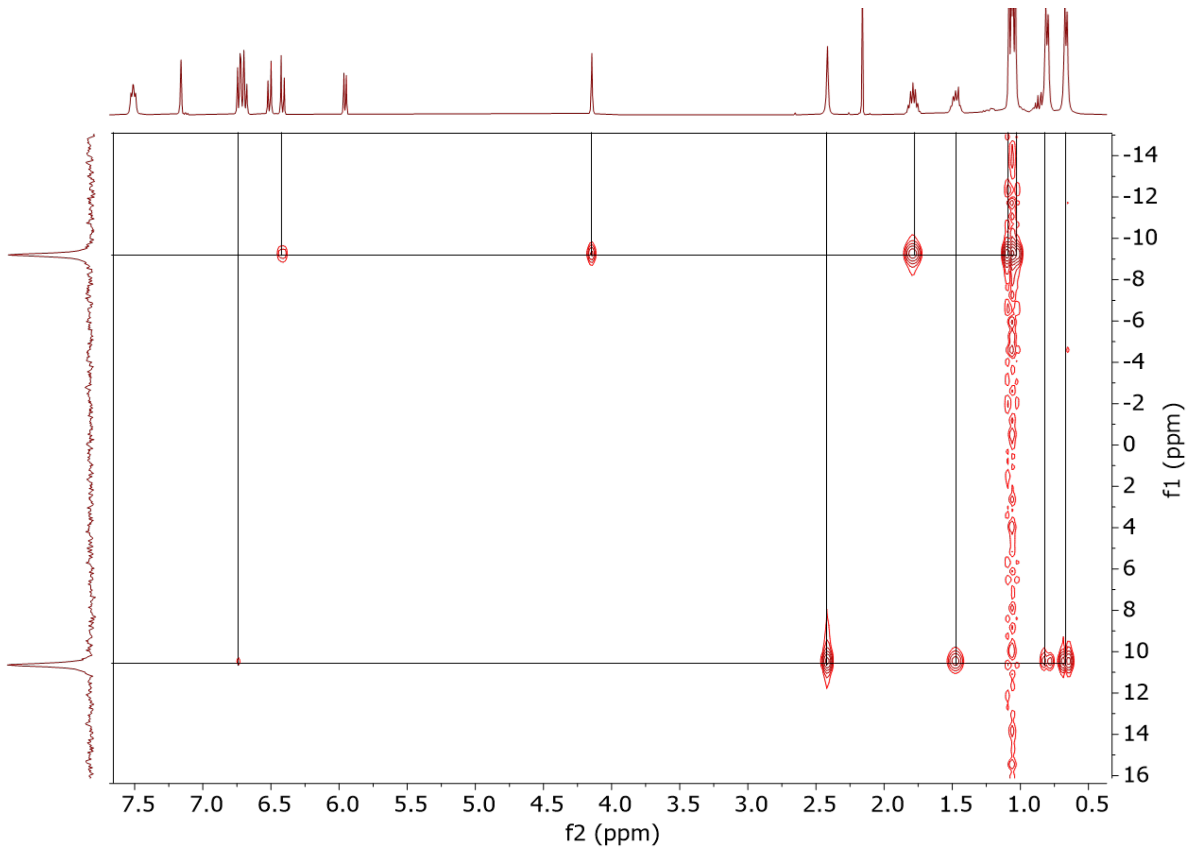

Figure S19: The ^{1}H-^{31}P HMBC NMR spectrum of complex **2** in C_6D_6 at 298 K.

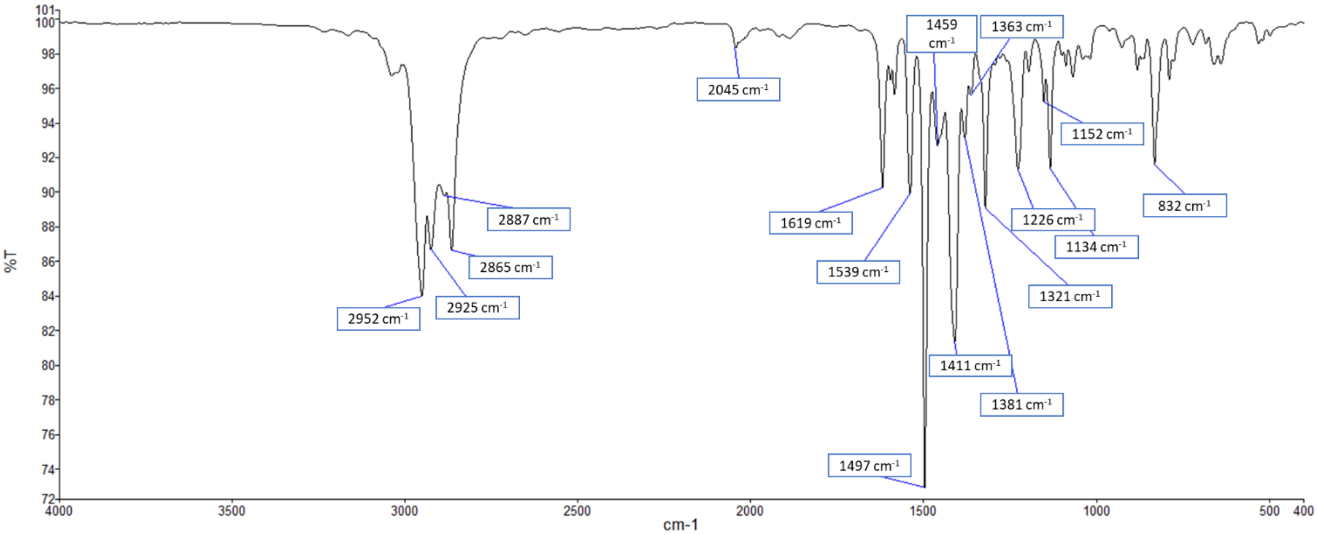

Figure S20: The ATR-IR spectrum of complex **2** measured as a film under N_2 flow.

[Cu_2(^{iPr}PNNP*)(μ-(1,4-bis(*p*-fluorophenyl)-1,2,3-triazolide)] (**3**)

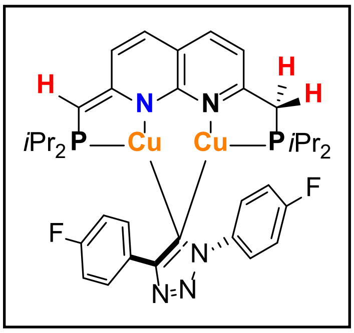

A solution of 1-azido-4-fluorobenzene (24.3 µL, 29.8 mg, 0.22 mmol, 2 equiv)* in THF (1.5 mL) was added dropwise over the course of 90 s to a stirred bright red solution of complex **2** (72.6 mg, 0.11 mmol) in THF (4.5 mL) at ambient temperature. After 2.5 h, the mixture was concentrated under a dynamic vacuum giving a red-brown film. Pentane (4 mL) was added and the resulting mixture was stirred for 1 h, after which the solution was pipetted off. The residue was extracted with Et_2O (3 mL), after which pentane (3 mL) was slowly added to the extract under stirring. The orange-red turbid mixture was filtered and the filtrate was stored at 243 K. Overnight, orange-red crystals had formed, which were isolated by pipetting off the supernatant. The orange-red crystals were washed with cold pentane (2 mL), and dried under a dynamic vacuum giving complex **3** as an orange-red solid (19.8 mg, 24%).

^{1}H NMR (400 MHz, C_6D_6, 298 K): δ = 8.69 - 8.61 (m, 2H, *m*-phenyl), 8.60 - 8.50 (m, 2H, *m*-phenyl), 6.83 - 6.73 (m, 2H, *o*-phenyl), 6.73 - 6.62 (m, 3H, overlapping *o*-phenyl and napy-*H*), 6.42 (d, $^3J_{H,H}$ = 9.2 Hz, 1H, napy-*H*), 6.34 (dd, $^3J_{H,H}$ = 9.2 Hz, $^4J_{H,P}$ = 1.8 Hz, 1H, napy-*H*), 5.97 (d, $^3J_{H,H}$ = 7.2 Hz, 1H, napy-*H*), 4.05 (d, $^2J_{H,P}$ = 2.5 Hz, 1H, methine-*H*), 2.34 (dd, $^2J_{H,H}$ = 17.2 Hz, $^2J_{H,P}$ = 7.9 Hz, 1H, geminal coupling, methylene-*H*), 2.28 (dd, $^2J_{H,H}$ = 17.2 Hz, $^2J_{H,P}$ = 7.9 Hz, 1H, geminal coupling, methylene-*H*), 1.46 (dhept, $^3J_{H,H}$ = 10.5 Hz, $^2J_{H,P}$ = 7.0 Hz, 2H, -C*H*(CH_3)$_2$), 1.17 - 1.04 (m, 2H, *overlapping with Et_2O* -C*H*(CH_3)$_2$), 0.92 - 0.78 (m, 6H, -CH(C*H*$_3$)$_2$), 0.78 - 0.64 (m, 6H, -CH(C*H*$_3$)$_2$), 0.47 - 0.30 (m, 12H, -CH(C*H*$_3$)$_2$) ppm.

^{13}C{^{1}H} NMR (101 MHz, C_6D_6, 298 K): δ = 166.3 (d, $^2J_{C,P}$ = 16.0 Hz), 162.8 (d, $^1J_{C,F}$ = 68.5 Hz), 160.4 (d, $^1J_{C,F}$ = 69.3 Hz), 157.6, 157.3, 156.5 (d, $^2J_{C,P}$ = 4.8 Hz), 138.9 (d, $^4J_{C,F}$ = 3.1 Hz), 134.4, 133.0, 130.7, 129.1 (d, $^3J_{C,P}$ = 2.3 Hz), 128.4, 126.7 (d, $^4J_{C,P}$ = 9.2 Hz), 122.0 (d, $^2J_{C,F}$ = 7.8 Hz), 118.9 (t, *overlapping dd*, $^5J_{C,P}$ = 2.3 Hz), 116.0 (d, $^3J_{C,F}$ = 22.6 Hz), 115.7 (d, $^3J_{C,F}$ = 21.3 Hz), 110.4 (d, $^4J_{C,P}$ = 3.3 Hz), 80.6 (d, $^1J_{C,P}$ = 38.9 Hz), 30.4 (d, $^1J_{C,P}$ = 16.4 Hz), 23.0 (d, $^1J_{C,P}$ = 9.4 Hz), 22.8 (d, $^1J_{C,P}$ = 9.1 Hz), 22.2 (d, $^1J_{C,P}$ = 12.4 Hz), 21.8 (d, $^1J_{C,P}$ = 13.1 Hz), 19.9 (d, $^2J_{C,P}$ = 7.9 Hz), 19.8 (d, $^2J_{C,P}$ = 7.9 Hz), 18.9 (d, $^2J_{C,P}$ = 10.7 Hz), 18.7 (d, $^2J_{C,P}$ = 10.7 Hz), 18.4 (d, $^2J_{C,P}$ = 2.2 Hz), 18.2 (d, $^2J_{C,P}$ = 1.9 Hz), 17.9 (d, $^2J_{C,P}$ = 2.6 Hz), 17.7 (d, $^2J_{C,P}$ = 1.9 Hz) ppm.

^{19}F NMR (376 MHz, C_6D_6, 298 K): δ = –116.8 - –116.9 (m, 1F), –116.9 - –117.1 (m, 1F) ppm.

^{31}P{^{1}H} NMR (162 MHz, C_6D_6, 298 K): δ = 8.8 (s, 1P), –2.4 (s, 1P) ppm.

ATR-IR (film, N_2 flow): ν = 2955 (m), 2925 (m), 2865 (m), 1618 (m), 1541 (m), 1519 (w), 1505 (s), 1496 (s), 1417 (s), 1322 (m), 1226 (m), 1152 (w), 1133 (m), 835 (m), 823 (w), 656 (w) cm^{-1}.

Anal. Calcd. For $C_{36}H_{43}Cu_2F_2N_5P_2$·0.2 Et_2O ($C_4H_{10}O$): C, 56.12; H, 5.76; N, 8.89. Found: C, 56.44; H, 5.36; N, 8.98. The 0.2 equiv of Et_2O are included as it is also observed in this ratio in the ^{1}H NMR spectrum.

* Note: In the synthesis of complex **3**, a few percent of the 1,4-triazole product is formed, as well as the formation of unidentified side products. Using two equiv of azide resulted in a product that was more easily purified from side products. A few percent of these side products can often be observed in the recrystallized batches of complex **3**, see for example the ^{31}P{^{1}H} NMR and ^{19}F NMR spectra in Figures S67 and S68.

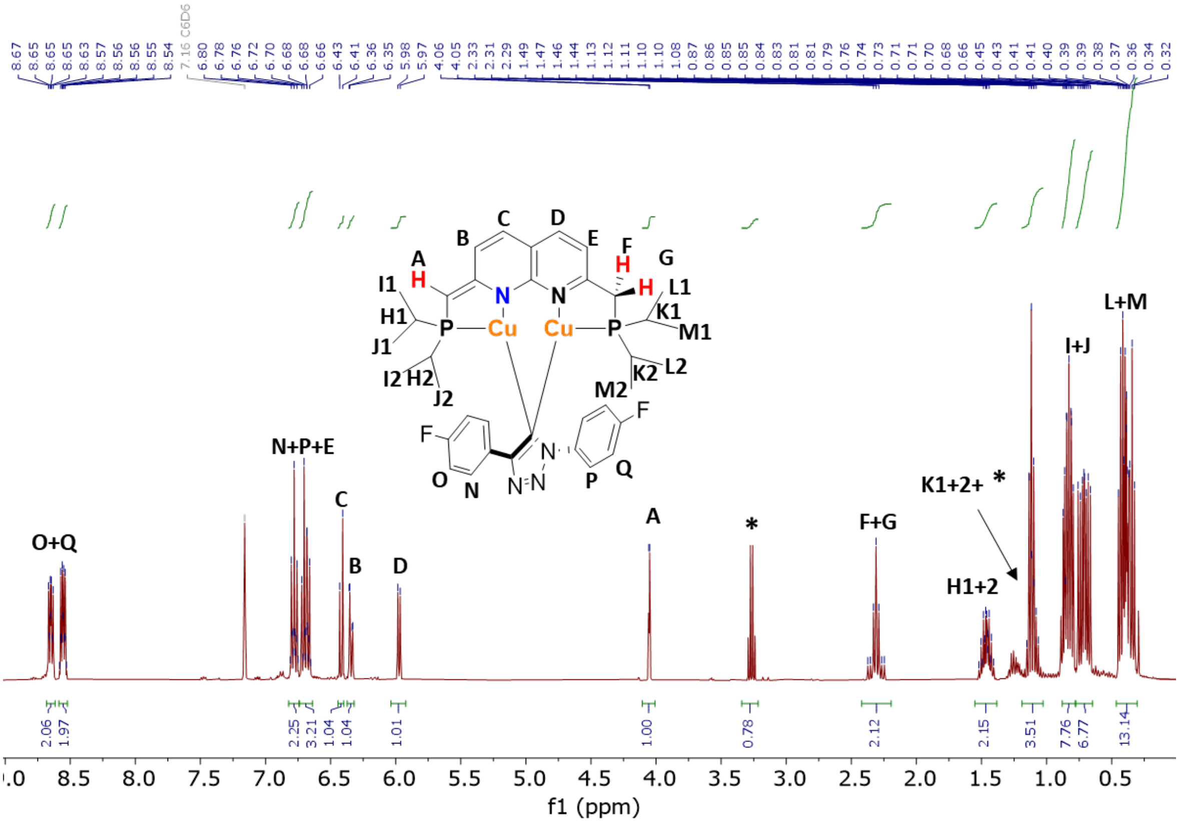

Figure S21: The ^{1}H NMR spectrum of complex **3** in C_6D_6 at 298 K. The resonances marked with * are assigned to residual Et_2O.

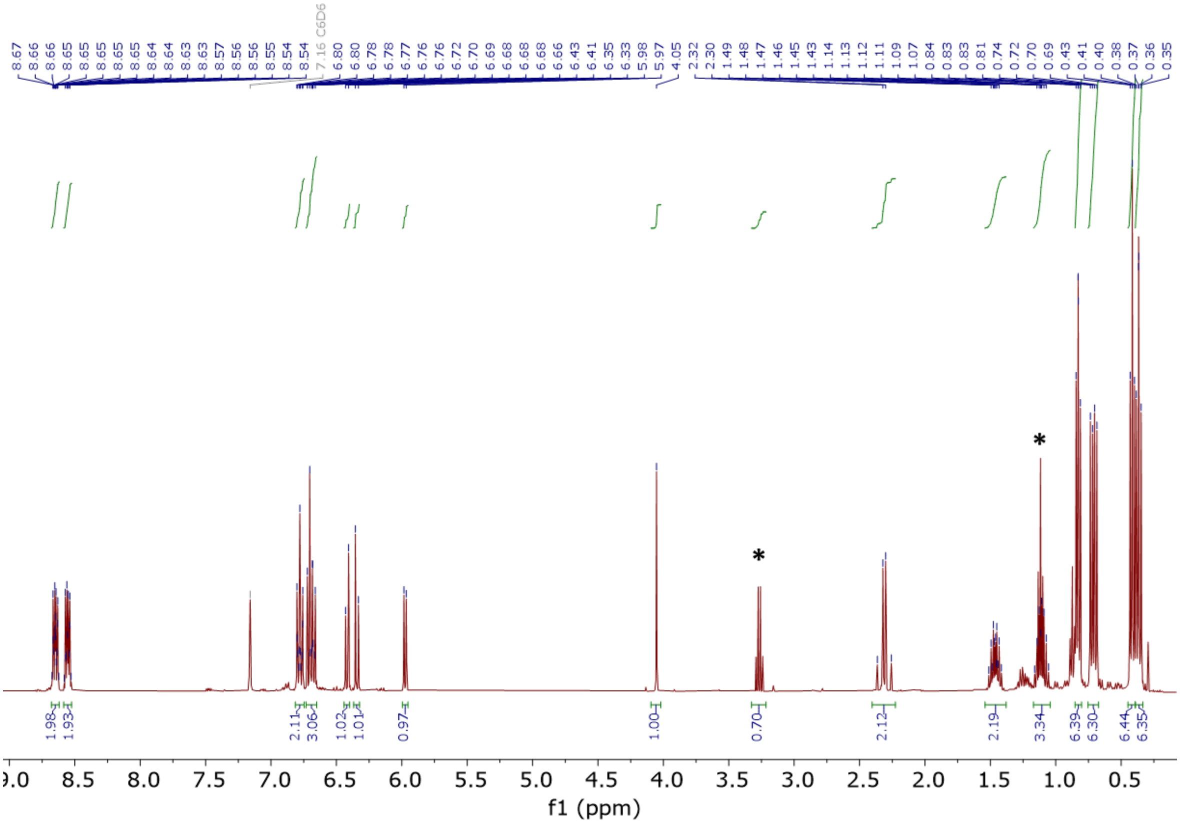

Figure S22: The $^{1}H\{^{31}P\}$ NMR spectrum of complex **3** in C_6D_6 at 298 K. The resonances marked with * are assigned to residual Et_2O.

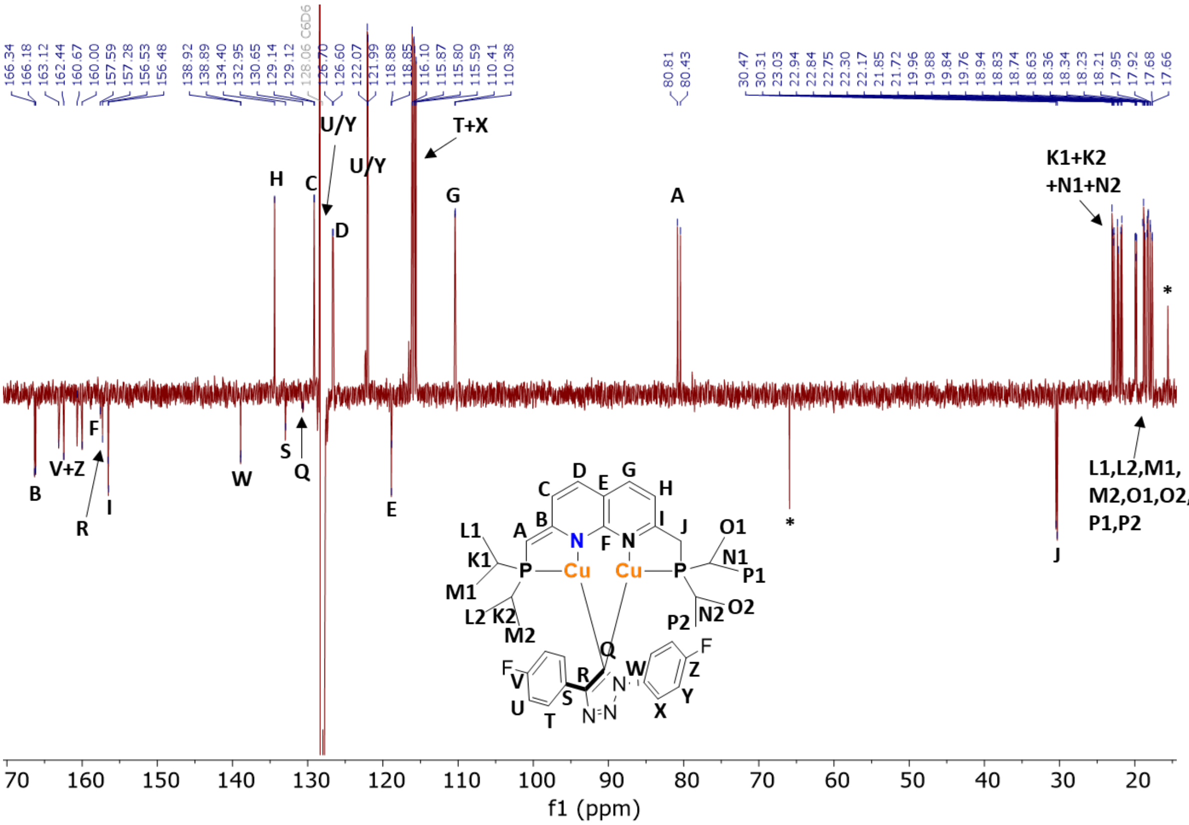

Figure S23: The $^{13}C\{^1H\}$ APT NMR spectrum of complex **3** in C_6D_6 at 298 K. The resonances marked with * are assigned to residual Et_2O.

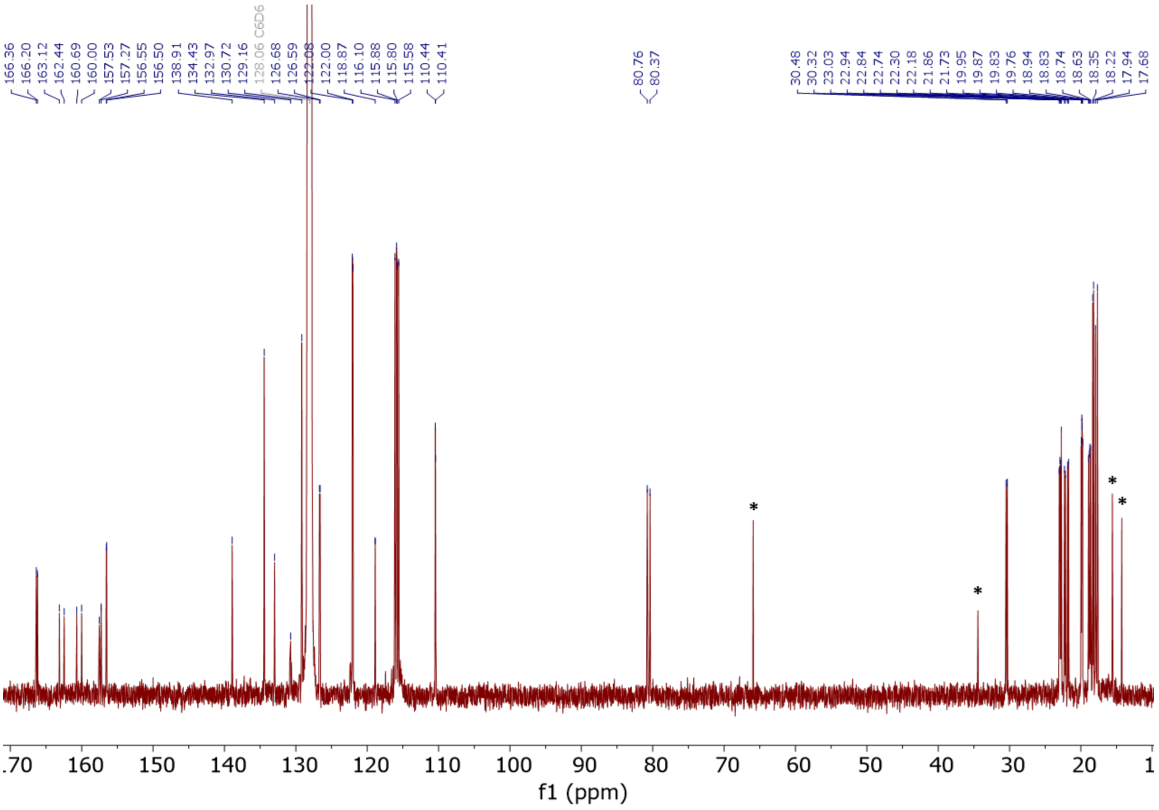

Figure S24: The $^{13}C\{^1H\}$ NMR spectrum of complex **3** in C_6D_6 at 298 K. The resonances marked with * are assigned to residual Et_2O and pentane.

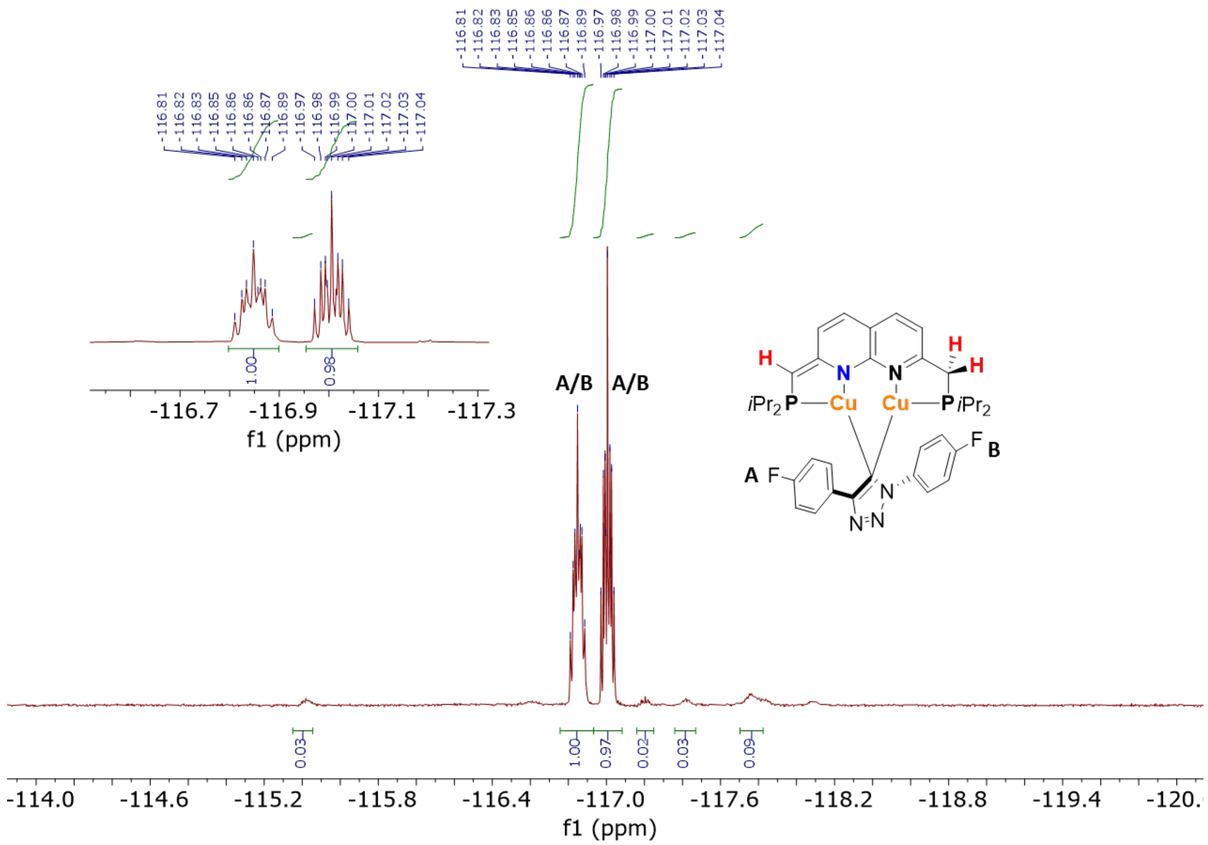

Figure S25: The ^{19}F NMR spectrum of complex **3** in C_6D_6 at 298 K. Some unidentified side products are observed.

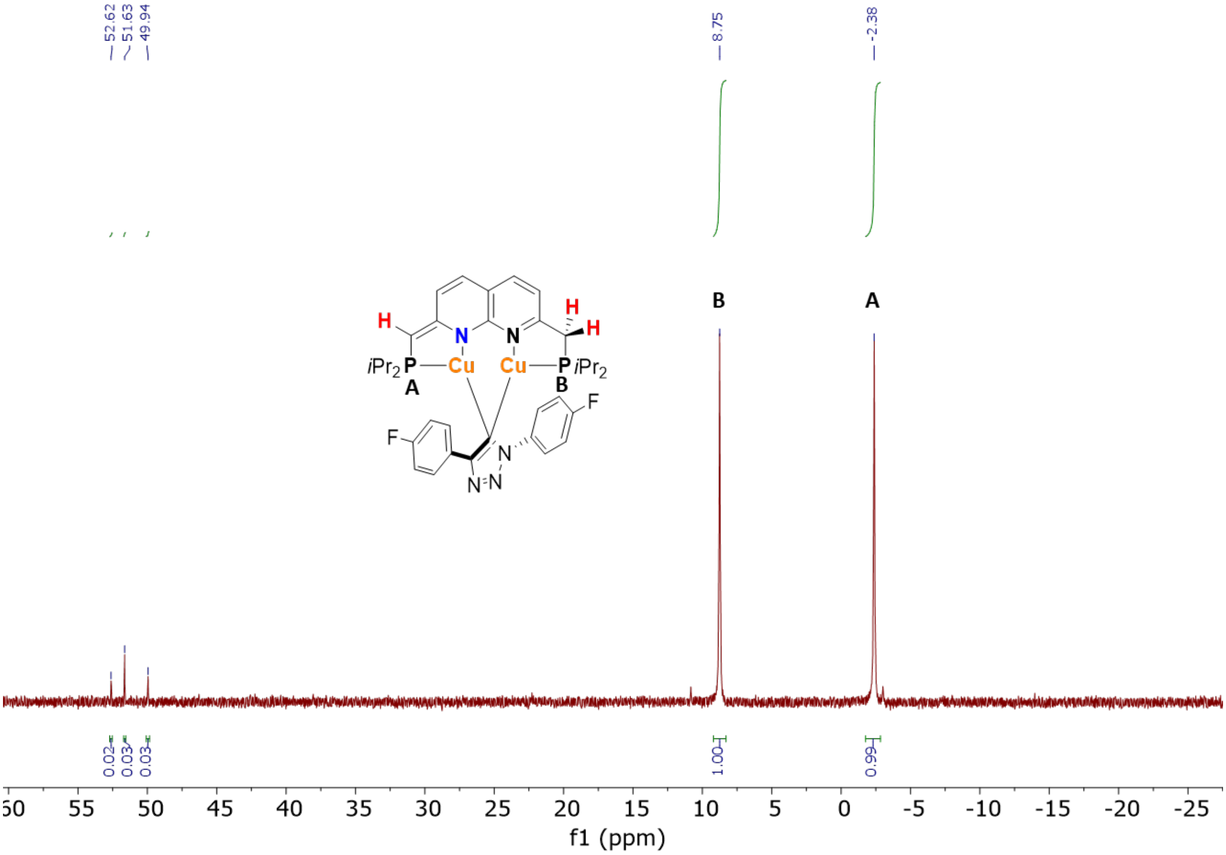

Figure S26: The $^{31}P\{^{1}H\}$ NMR spectrum of complex **3** in C_6D_6 at 298 K. The resonances observed between δ = 55-50 ppm are from unidentified side products.

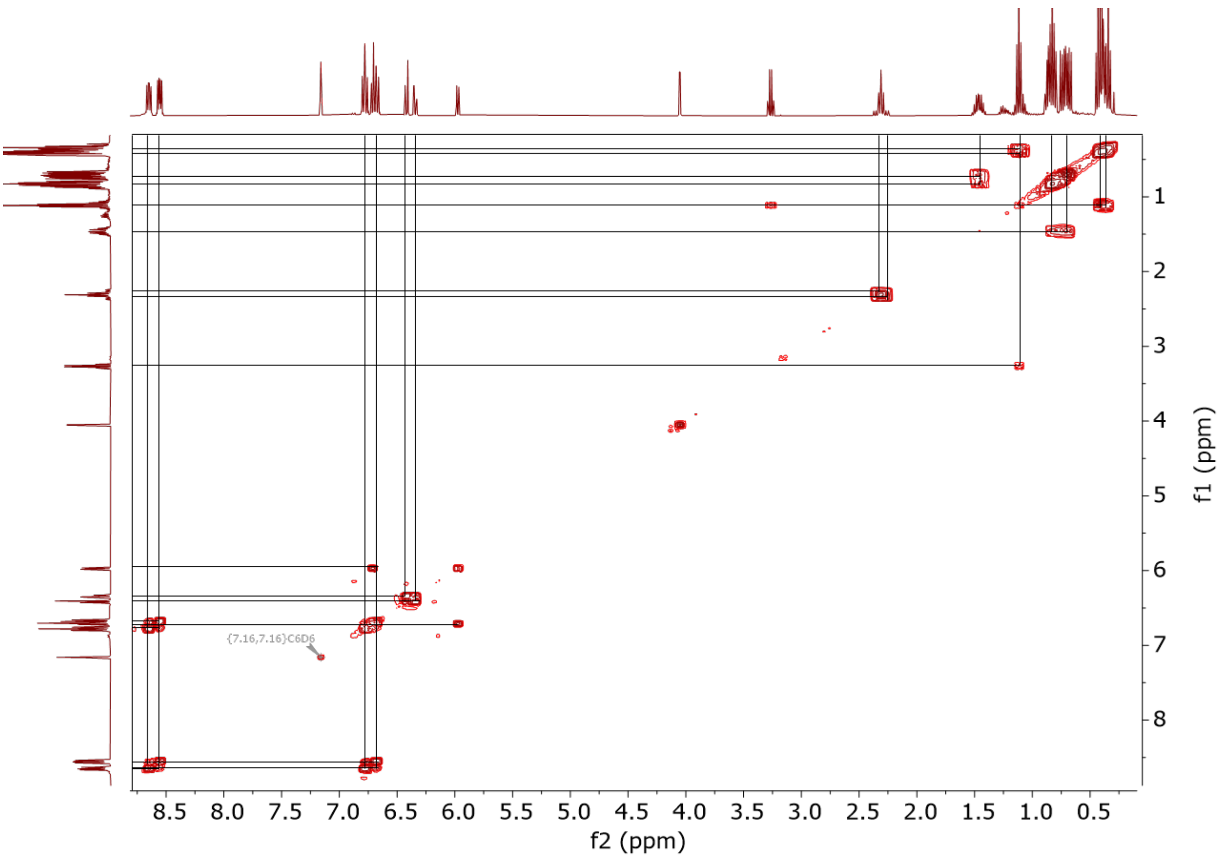

Figure S27: The ^{1}H-^{1}H gCOSY NMR spectrum of complex **3** in C_6D_6 at 298 K.

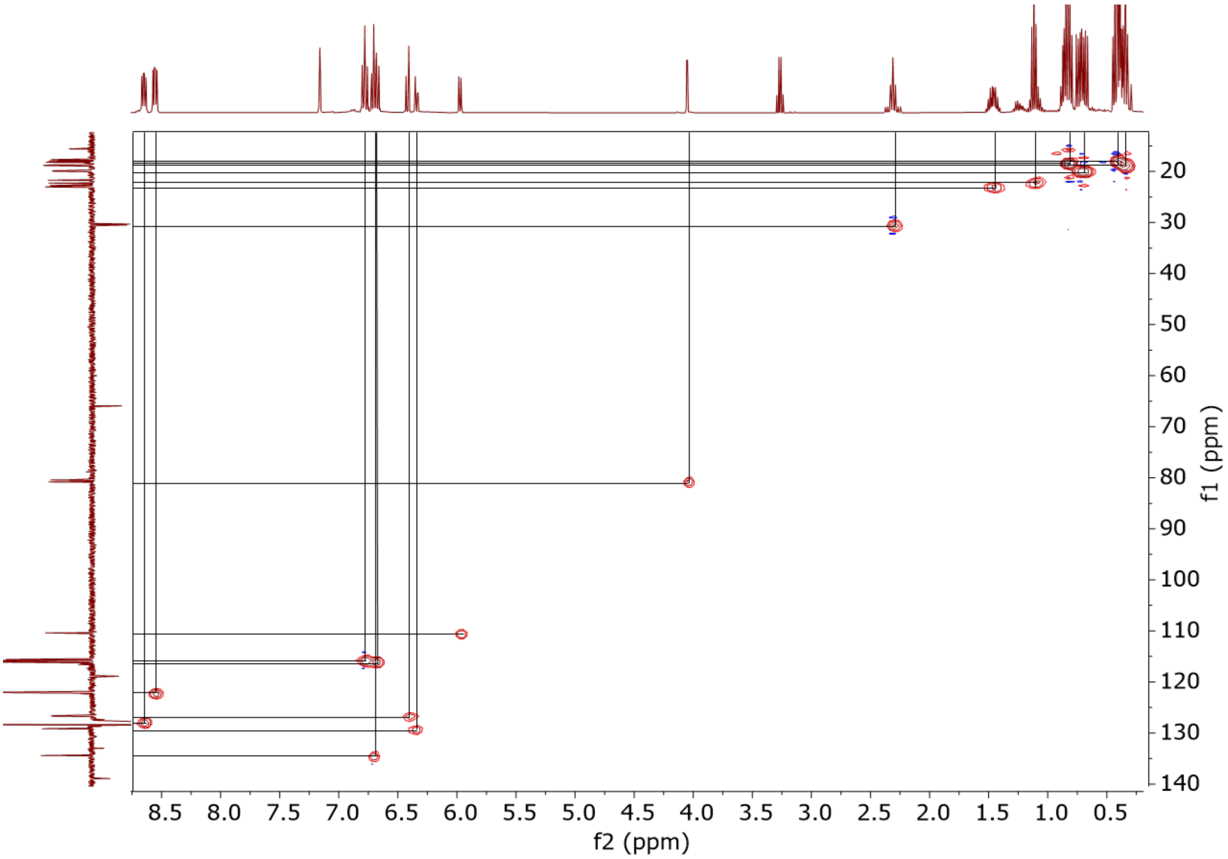

Figure S28: The ^{1}H-^{13}C ASAPHMQC NMR spectrum of complex **3** in C_6D_6 at 298 K.

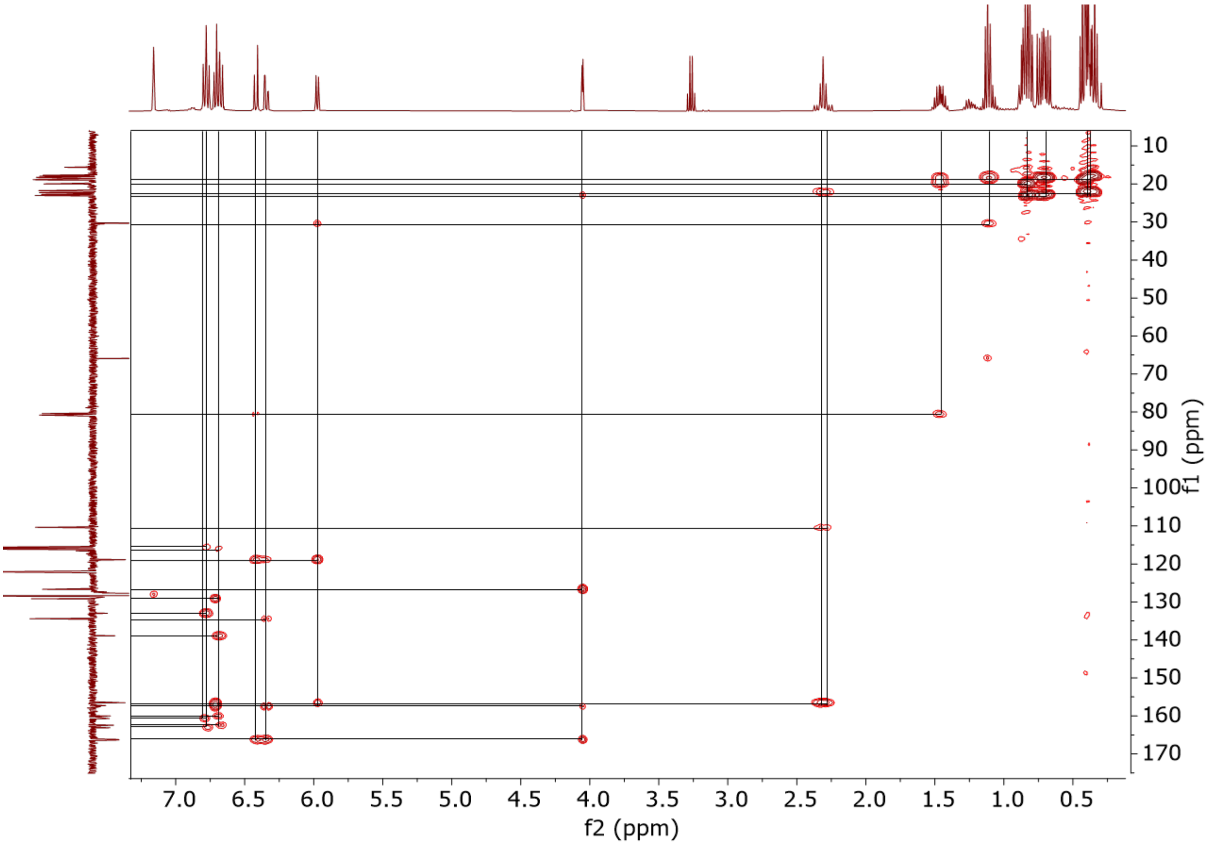

Figure S29: The ^{1}H-^{13}C gHMBCAD NMR spectrum of complex **3** in C_6D_6 at 298 K.

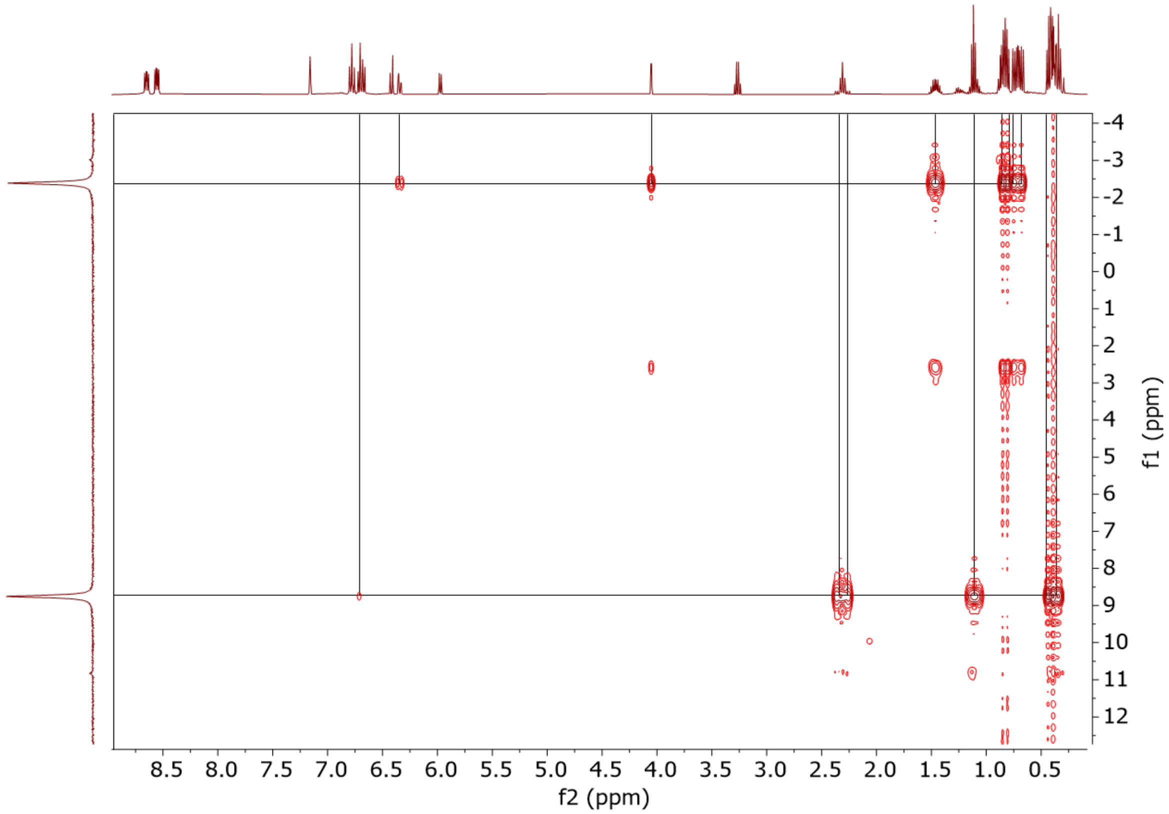

Figure S30: The ^{1}H-^{31}P gHMBCAD NMR spectrum of complex **3** in C_6D_6 at 298 K.

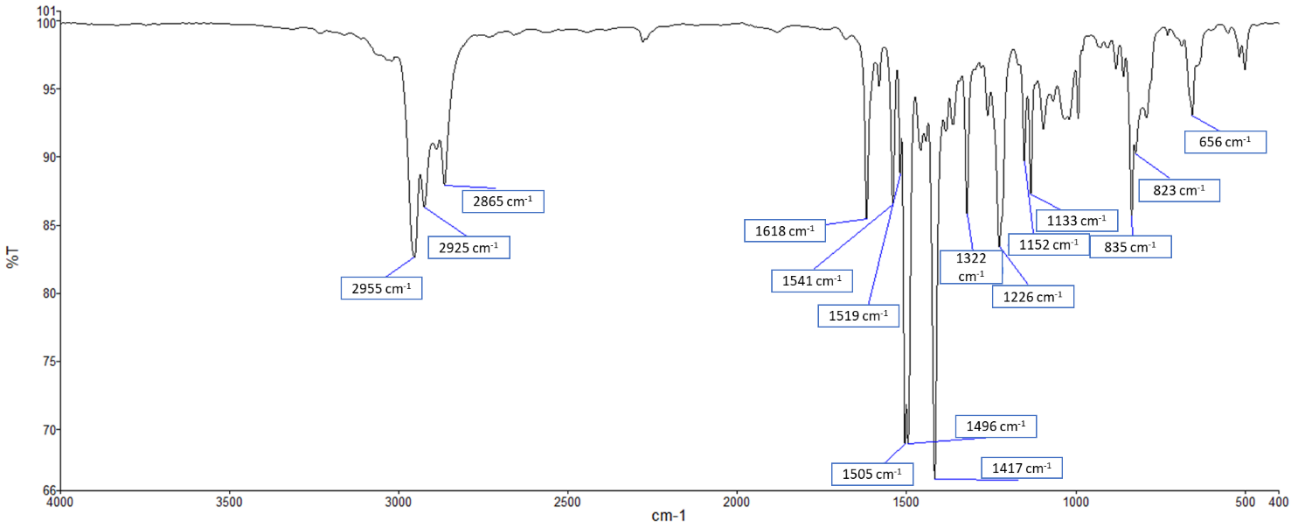

Figure S31: The ATR-IR spectrum of complex **3** measured as a film under N_2 flow.

1,4-bis(4-fluorophenyl)-1H-1,2,3-triazole and 1,5-bis(4-fluorophenyl)-1H-1,2,3-triazole

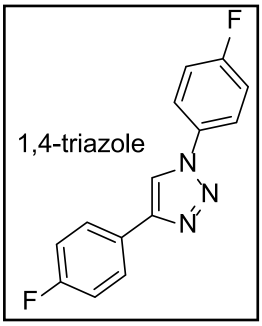

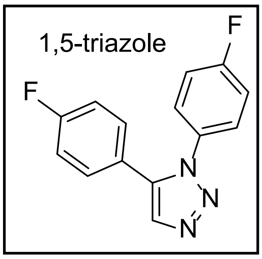

The azide-alkyne Huisgen cycloaddition reaction was performed to synthesize both isomers of the corresponding 1,2,3-triazoles described in the main text. The synthesis is based on a literature procedure.[7] *Reaction performed in air:* A round bottom flask was charged with 1-ethynyl-4-fluorobenzene (0.42 mL, 440 mg, 3.7 mmol) and 1-azido-4-fluorobenzene (256.9 mg of liquid, 1.874 mmol) and toluene (4 mL, not dried). The resulting orange/brown mixture was refluxed for 19.5 h. After the reaction, a darker brown mixture had formed, containing a small amount of crystals. The mixture was slowly allowed to cool to ambient temperature. Off-white crystals were grown in 3 h and filtered off and washed with petroleum ether (40-60 °C) (10 mL). The crystals were dried under a dynamic vacuum, giving the 1,4-triazole as off-white crystals in a yield of 168.8 mg (35%). The red/brown decanted mother liquor was concentrated under a dynamic vacuum giving the 1,5-triazole as a beige solid in a yield of 300 mg (57%), containing 11 mol% of 1-ethynyl-4-fluorobenzene as an impurity according to ^1H NMR analysis ($CDCl_3$).

The NMR resonances of the products were assigned as either the 1,4-triazole or the 1,5-triazole based on the work published by Creary and co-workers.[8] Note: The 1,4-triazole product has a poor solubility in most common organic solvents. Dissolving appreciable amounts of the compound for characterization by NMR was achieved in DMSO-d_6. Low solubility is observed in C_6D_6, $CDCl_3$ and THF. Note2: The 1,5-triazole product has a significantly higher solubility in most common organic solvents in comparison with the 1,4-triazole product. Therefore, high purity product can be obtained either through recrystallization or chromatography methods.

1,4-triazole: ^1H NMR (400 MHz, DMSO-d_6, 298 K): δ = 9.27 (s, 1H, triazole-*H*), 8.02 - 7.92 (m, 4H, *m*-phenyl), 7.55 - 7.45 (m, 2H, *o*-phenyl), 7.40 - 7.31 (m, 2H, *o*-phenyl) ppm.

^1H NMR (400 MHz, C_6D_6, 298 K): δ = 7.78 - 7.66 (m, 2H, *m*-phenyl), 7.10 - 7.05 (m, 2H, *m*-phenyl), 7.03 (s, 1H, triazole-*H*), 6.94 - 6.83 (m, 2H, *o*-phenyl), 6.70 - 6.57 (m, 2H, *o*-phenyl) ppm.

^1H NMR (400 MHz, $CDCl_3$, 298 K): δ = 8.10 (s, 1H), 7.93 - 7.84 (m, 2H), 7.82 - 7.71 (m, 2H), 7.26 (*m, overlaping with CHCl$_3$*, 2H), 7.20 - 7.11 (m, 2H) ppm.

^1H NMR (400 MHz, THF-h_8, 298 K): δ = 8.71 (s, 1H, triazole-*H*), 8.00 - 7.87 (m, 4H, *m*-phenyl), 7.39 - 7.29 (m, 2H, *o*-phenyl), 7.24 - 7.13 (m, 2H, *o*-phenyl) ppm.

$^{13}C\{^1H\}$ NMR (101 MHz, DMSO-d_6, 298 K): δ = 163.1 (d, $J_{F,C}$ = 31.8 Hz), 160.6 (d, $J_{F,C}$ = 32.3 Hz), 146.5, 133.18 (d, $J_{F,C}$ = 3.4 Hz), 127.3, 126.8 (d, $J_{F,C}$ = 2.9 Hz), 122.4 (d, $J_{F,C}$ = 8.7 Hz), 119.9, 116.8 (d, $J_{F,C}$ = 23.1 Hz), 116.0 (d, $J_{F,C}$ = 21.7 Hz) ppm.

^{19}F NMR (376 MHz, DMSO-d_6, 298 K): δ = –112.9 (tt, $^3J_{F,H}$ = 8.5 Hz, $^4J_{F,H}$ = 4.7 Hz, 1F), –113.4 (tt, $^3J_{F,H}$ = 9.0 Hz, $^4J_{F,H}$ = 5.5 Hz, 1F) ppm.

^{19}F NMR (376 MHz, C_6D_6, 298 K): δ = –113.1 (tt, $^3J_{F,H}$ = 8.3 Hz, $^4J_{F,H}$ = 4.6 Hz, 1F), –113.2 (tt, $^3J_{F,H}$ = 8.6 Hz, $^4J_{F,H}$ = 5.2 Hz, 1F) ppm.

^{19}F NMR (376 MHz, $CDCl_3$, 298 K): δ = –112.0 (tt, $^3J_{F,H}$ = 8.1 Hz, $^4J_{F,H}$ = 4.2 Hz, 1F), –112.9 (tt, $^3J_{F,H}$ = 8.7 Hz, $^4J_{F,H}$ = 4.4 Hz, 1F) ppm.

^{19}F NMR (376 MHz, THF-h_8, 298 K): δ = –115.0 (tt, $^3J_{F,H}$ = 8.3 Hz, $^4J_{F,H}$ = 4.3 Hz, 1F), –115.5 (tt, $^3J_{F,H}$ = 9.3 Hz, $^4J_{F,H}$ = 4.4 Hz, 1F) ppm.

ATR-IR (film, N_2 flow): ν = 3104 (m), 1606 (w), 1561 (m), 1517 (s), 1493 (s), 1409 (w), 1226 (s), 1159 (m), 1098 (w), 1041 (m), 995 (w), 843 (s), 817 (s), 646 (m), 539 (m) cm^{-1}.

Anal. Calcd. For $C_{14}H_9F_2N_3$: C, 65.37; H, 3.53; N, 16.33. Found: C, 65.29; H, 3.52; N, 16.40.

1,5-triazole: ^1H NMR (400 MHz, DMSO-d_6, 298 K): δ = 8.12 (s, 1H, triazole-*H*), 7.53 - 7.45 (m, 2H, *m*-phenyl), 7.44 - 7.30 (m, 4H, overlapping *m*-phenyl and *o*-phenyl), 7.31 - 7.22 (m, 2H, *o*-phenyl) ppm.

^1H NMR (400 MHz, $CDCl_3$, 298 K): δ = 7.85 (s, 1H, triazole-*H*), 7.38 - 7.30 (m, 2H, *m*-phenyl), 7.25 - 7.16 (m, 2H, *m*-phenyl), 7.16 - 7.11 (m, 2H, *o*-phenyl), 7.11 - 7.02 (m, 2H, *o*-phenyl) ppm.

$^{13}C\{^1H\}$ NMR (101 MHz, DMSO-d_6, 298 K): δ = 163.6 (d, $J_{F,C}$ = 26.9 Hz), 161.1 (d, $J_{F,C}$ = 26.6 Hz), 137.0, 133.2, 132.5 (d, $J_{F,C}$ = 3.0 Hz), 130.9 (d, $J_{F,C}$ = 8.6 Hz), 128.1 (d, $J_{F,C}$ = 9.1 Hz), 122.7 (d, $J_{F,C}$ = 3.2 Hz), 116.6 (d, $J_{F,C}$ = 23.0 Hz), 116.0 (d, $J_{F,C}$ = 22.0 Hz) ppm.

^{19}F NMR (376 MHz, DMSO-d_6, 298 K): δ = –111.4 (tt, $^3J_{F,H}$ = 8.5 Hz, $^4J_{F,H}$ = 4.4 Hz, 1F), –111.6 (tt, $^3J_{F,H}$ = 8.9 Hz, $^4J_{F,H}$ = 4.9 Hz, 1F) ppm.

^{19}F NMR (376 MHz, $CDCl_3$, 298 K): δ = –110.6 (tt, $^3J_{F,H}$ = 8.1 Hz, $^4J_{F,H}$ = 5.4 Hz, 1F), –110.8 (tt, $^3J_{F,H}$ = 8.2 Hz, $^4J_{F,H}$ = 4.7 Hz, 1F) ppm.

ATR-IR (film, N_2 flow): ν = 3056 (w), 2966 (w), 1608 (w), 1510 (s), 1493 (s), 1216 (m), 1161 (m) 1140 (m), 1097 (m), 1052 (m), 993 (m), 968 (w), 815 (m), 553 (w), 524 (w) cm^{-1}.

Anal. Calcd. For $C_{14}H_9F_2N_3 \cdot 0.1\ C_8H_5F$ (alkyne): C, 66.02; H, 3.56; N, 15.61. Found: C, 65.43; H, 3.72; N, 15.06. *

* The calculated value takes into account the presence of 0.1 equiv of 1-ethynyl-4-fluorobenzene as observed in the ^1H NMR spectrum (11%). The small deviation for the found N content can be explained by a slightly higher ratio of 1-ethynyl-4-fluorobenzene within the accuracy of the ^1H NMR analysis.

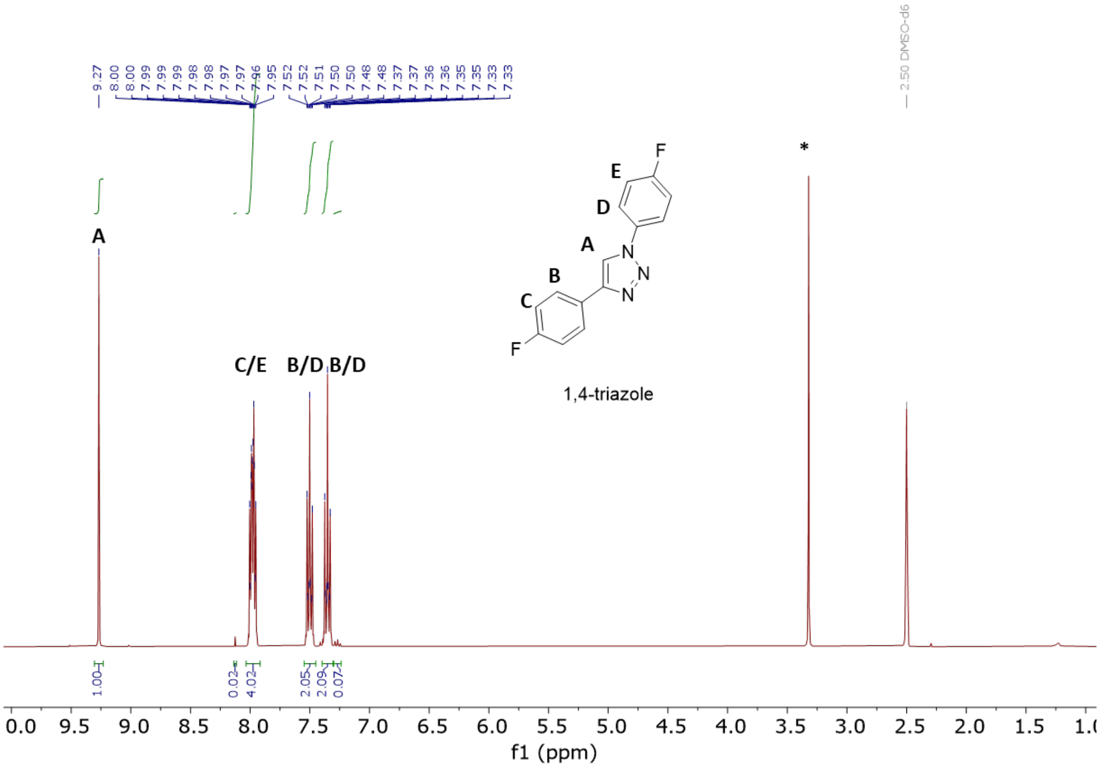

Figure S32: The ^{1}H NMR spectrum of 1,4-bis(4-fluorophenyl)-1H-1,2,3-triazole in DMSO-d_6 at 298 K. The resonance marked with a star is assigned to residual water in the DMSO-d_6.

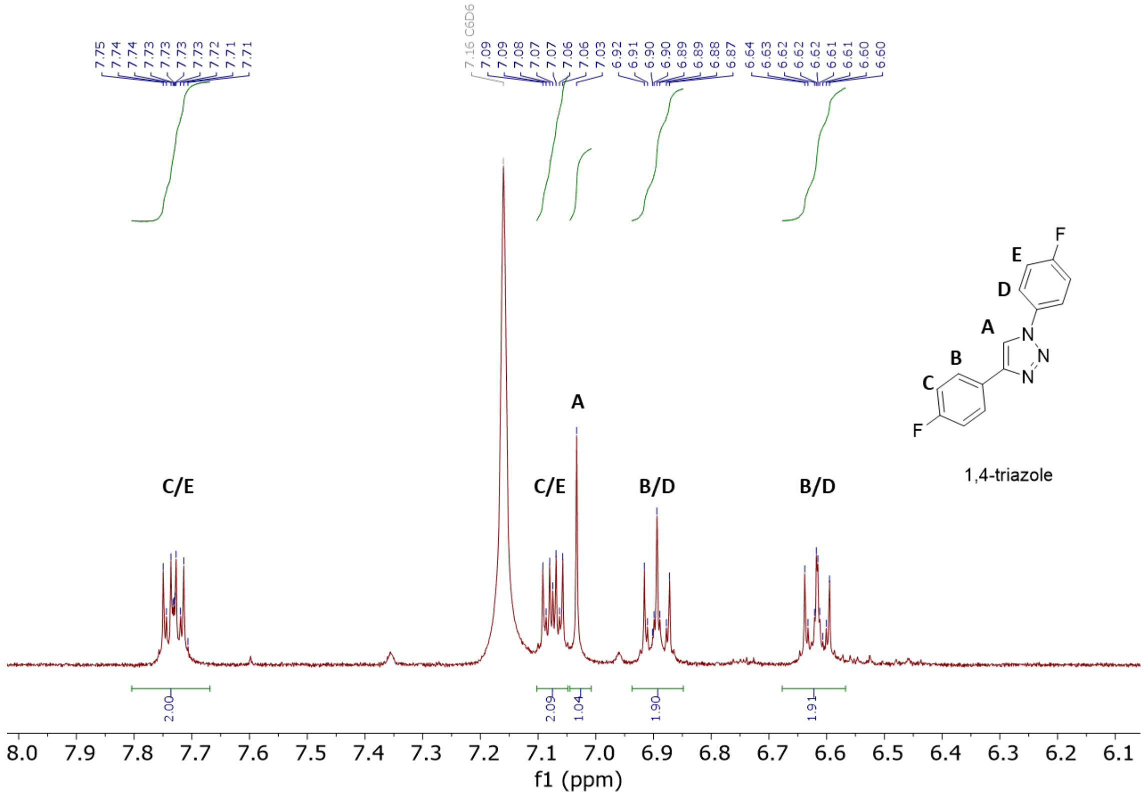

Figure S33: A zoom-in of the aromatic region of the ^{1}H NMR spectrum of 1,4-bis(4-fluorophenyl)-1H-1,2,3-triazole in C_6D_6 at 298 K.

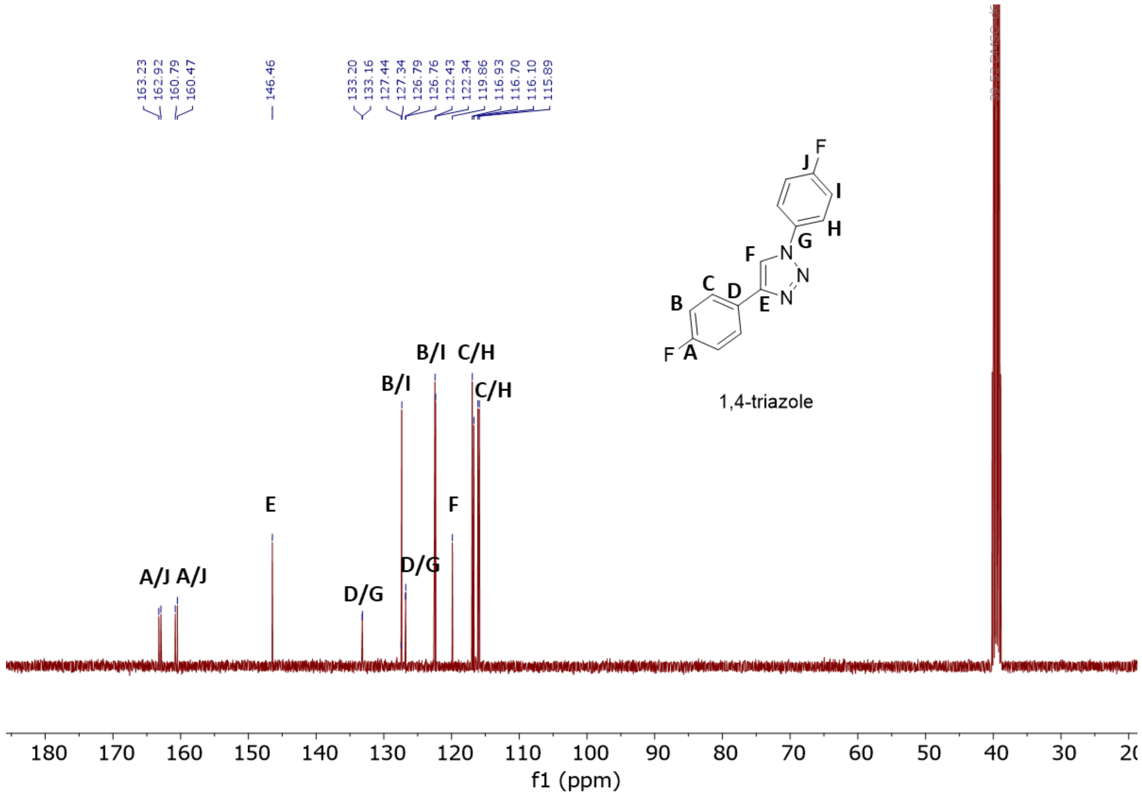

Figure S34: The $^{13}C\{^{1}H\}$ NMR spectrum of 1,4-bis(4-fluorophenyl)-1H-1,2,3-triazole in DMSO-d_6 at 298 K.

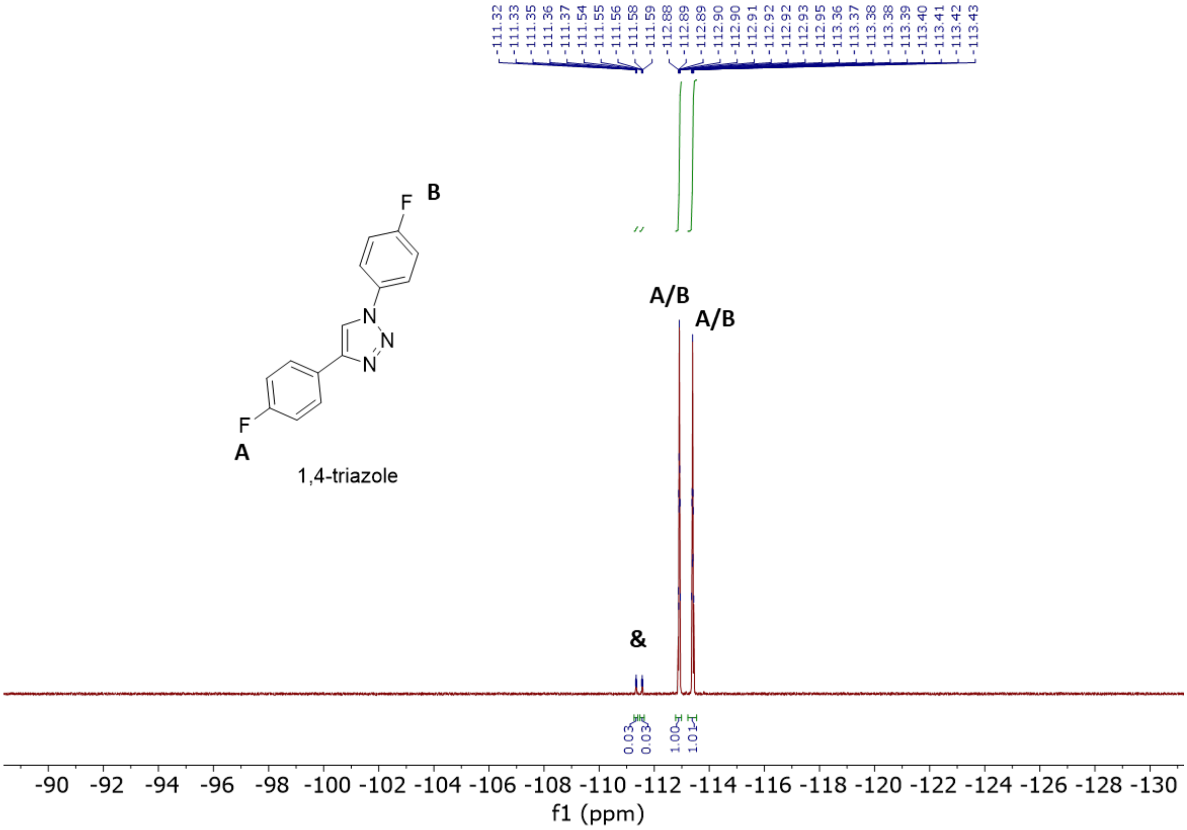

Figure S35: The ^{19}F NMR spectrum of 1,4-bis(4-fluorophenyl)-1H-1,2,3-triazole in DMSO-d_6 at 298 K. The resonance marked with & is assigned to the isomeric 1,5-triazole present as minor byproduct.

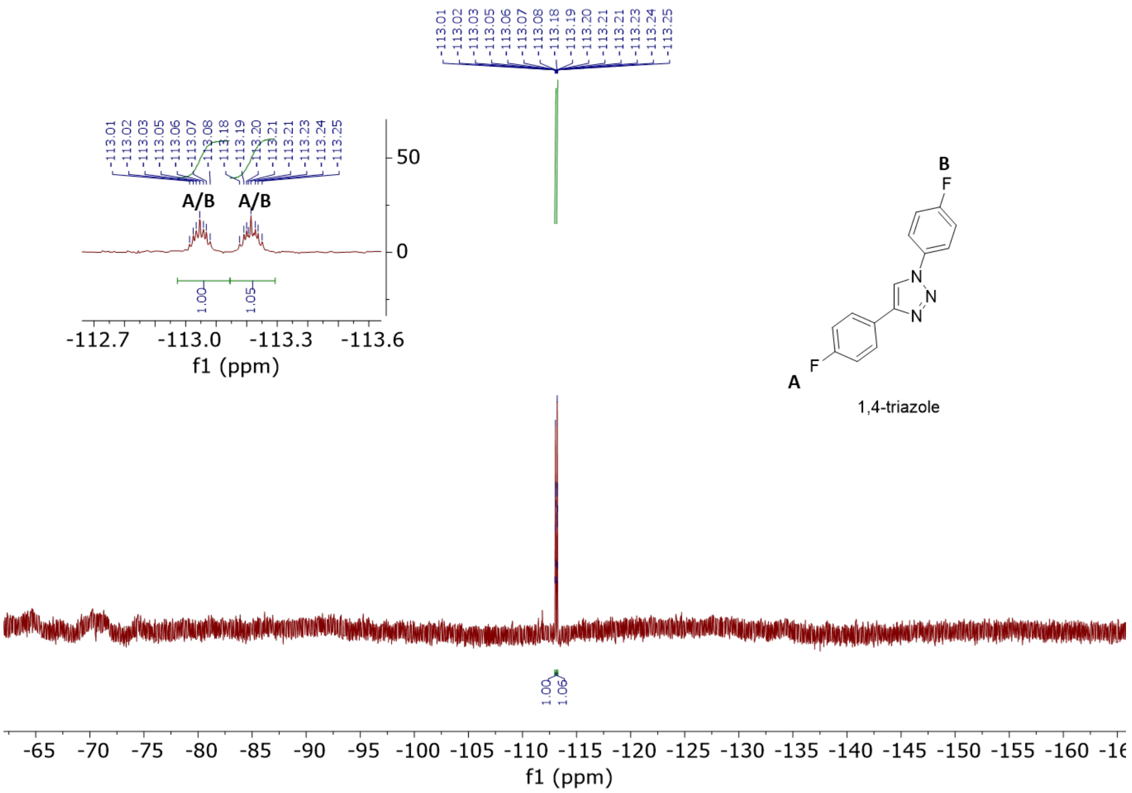

Figure S36: The ^{19}F NMR spectrum of 1,4-bis(4-fluorophenyl)-1H-1,2,3-triazole in C_6D_6 at 298 K. Between -60 until 75 ppm the background displays broad humps, which are an artefact in the probe of the NMR spectrometer.

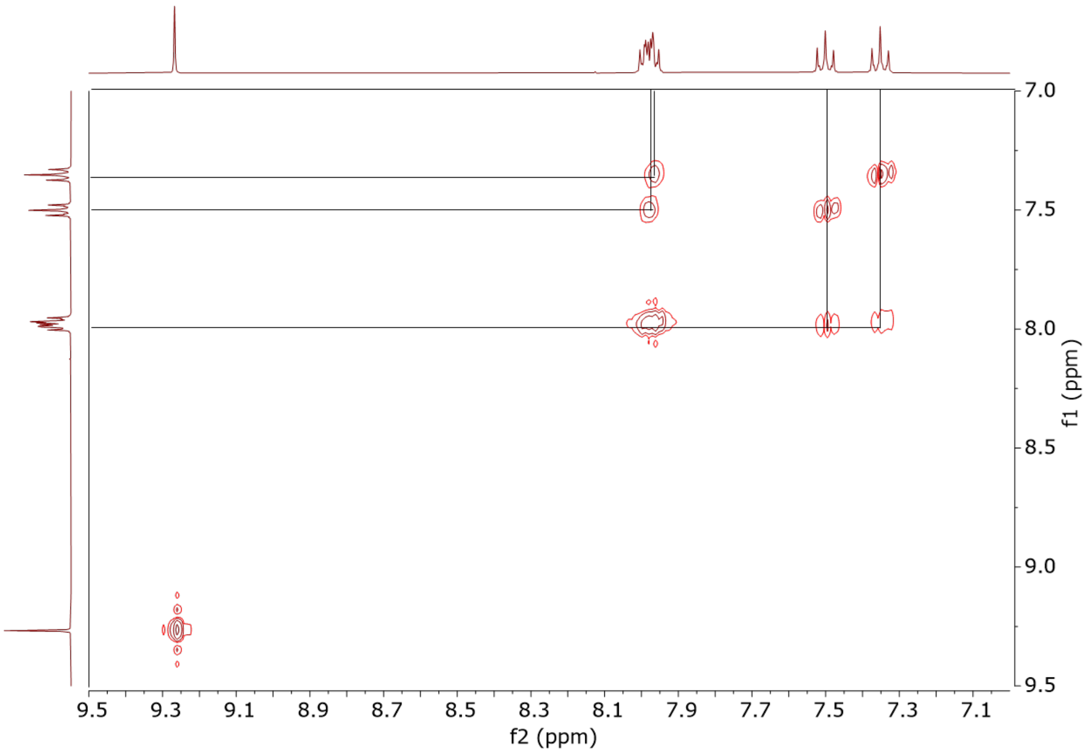

Figure S37: The ^{1}H-^{1}H COSY NMR spectrum of 1,4-bis(4-fluorophenyl)-1H-1,2,3-triazole in DMSO-d_6 at 298 K.

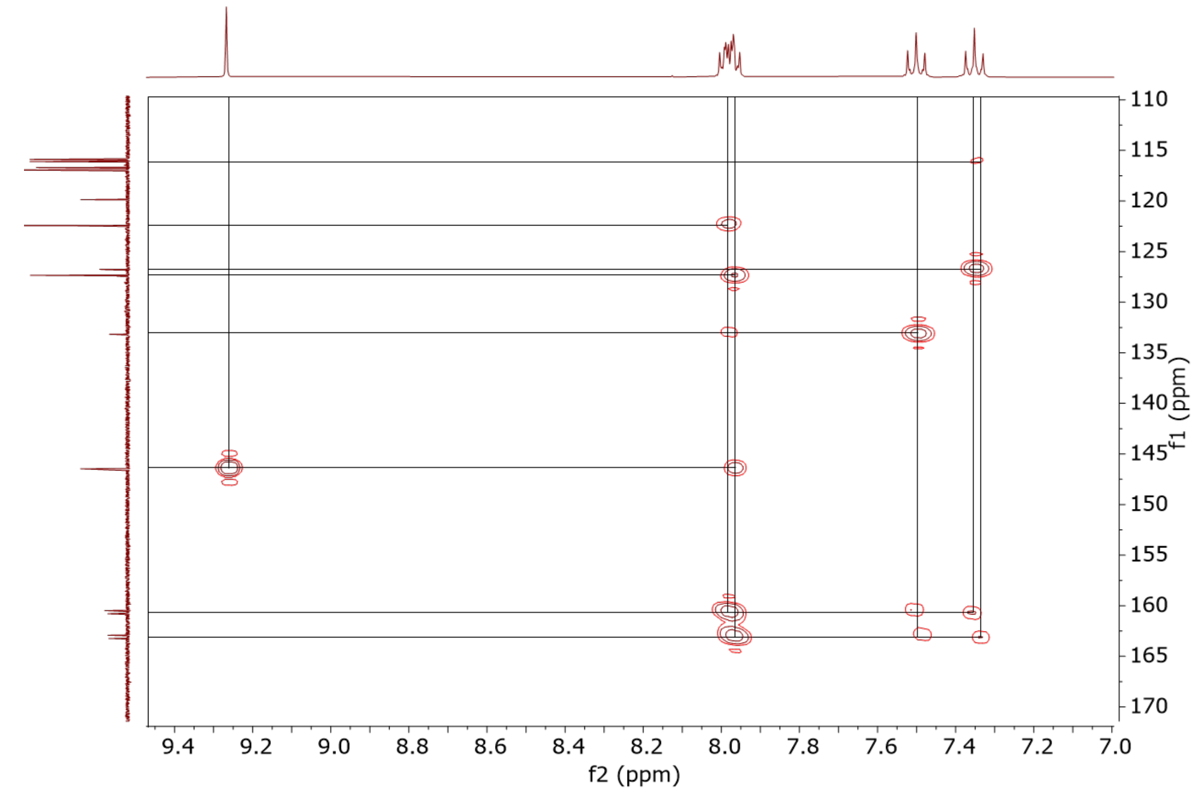

Figure S38: The ^{1}H-^{13}C HSQC NMR spectrum of 1,4-bis(4-fluorophenyl)-1H-1,2,3-triazole in DMSO-d_6 at 298 K.

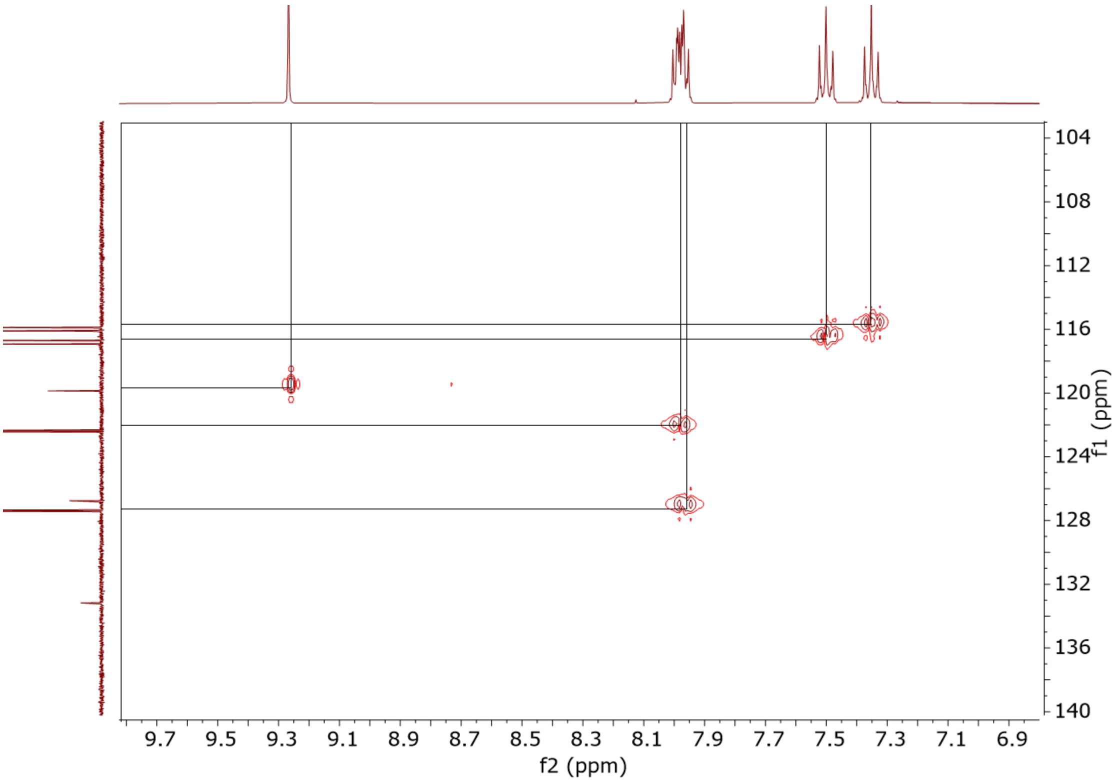

Figure S39: The ^{1}H-^{13}C HMBC NMR spectrum of 1,4-bis(4-fluorophenyl)-1H-1,2,3-triazole in DMSO-d_6 at 298 K.

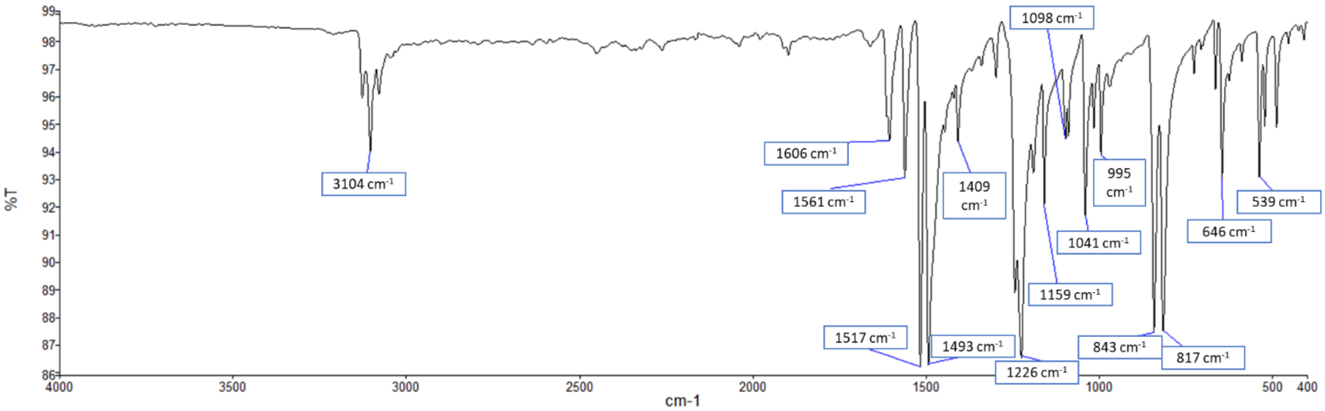

Figure S40: The ATR-IR spectrum of 1,4-bis(4-fluorophenyl)-1H-1,2,3-triazole measured as a solid under air.

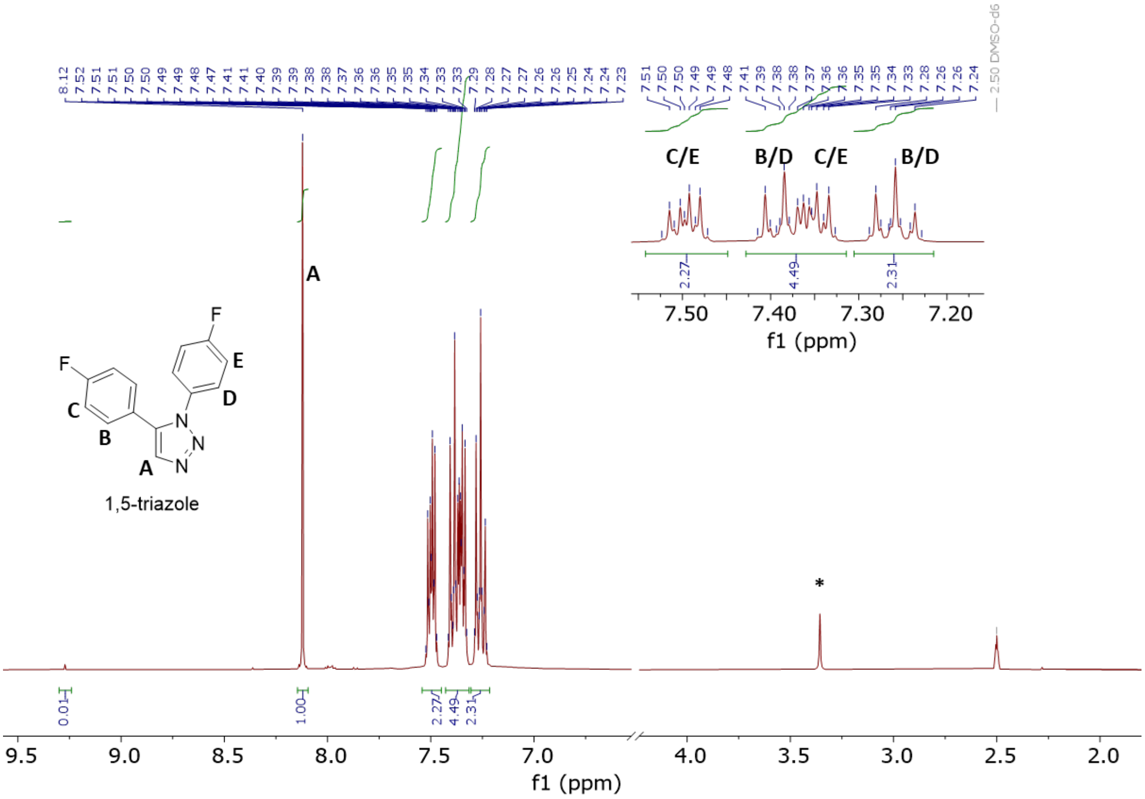

Figure S41: The ^{1}H NMR spectrum of 1,5-bis(4-fluorophenyl)-1H-1,2,3-triazole in DMSO-d_6 at 298 K. The resonance marked with a star is assigned to residual water in the DMSO-d_6.

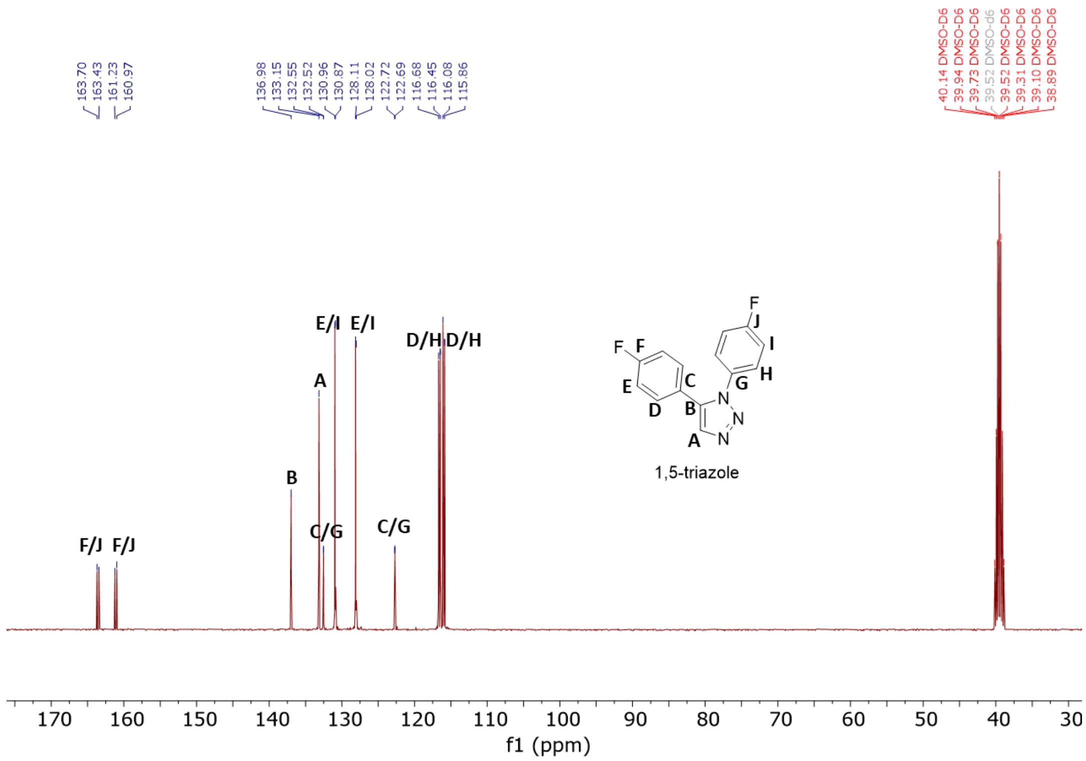

Figure S42: The $^{13}C\{^{1}H\}$ NMR spectrum of 1,5-bis(4-fluorophenyl)-1H-1,2,3-triazole in DMSO-d_6 at 298 K.

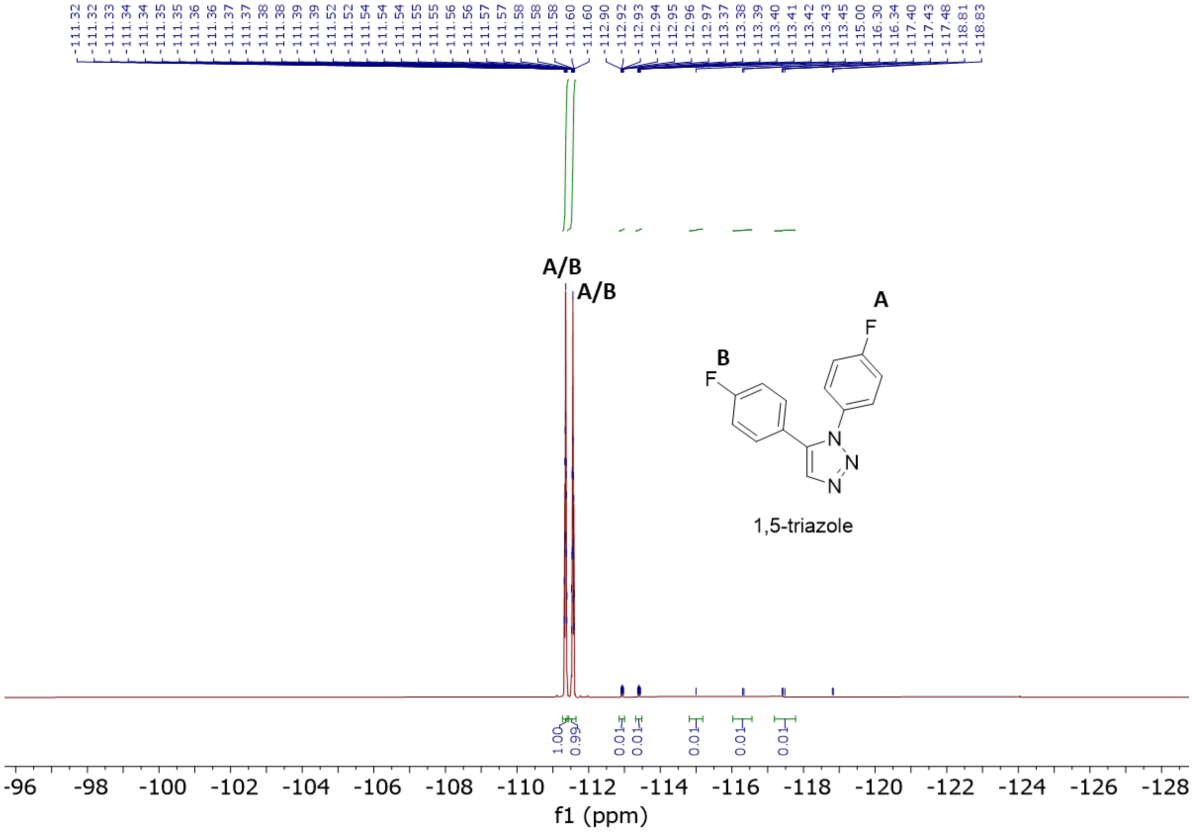

Figure S43: The ^{19}F NMR spectrum of 1,5-bis(4-fluorophenyl)-1H-1,2,3-triazole in DMSO-d_6 at 298 K.

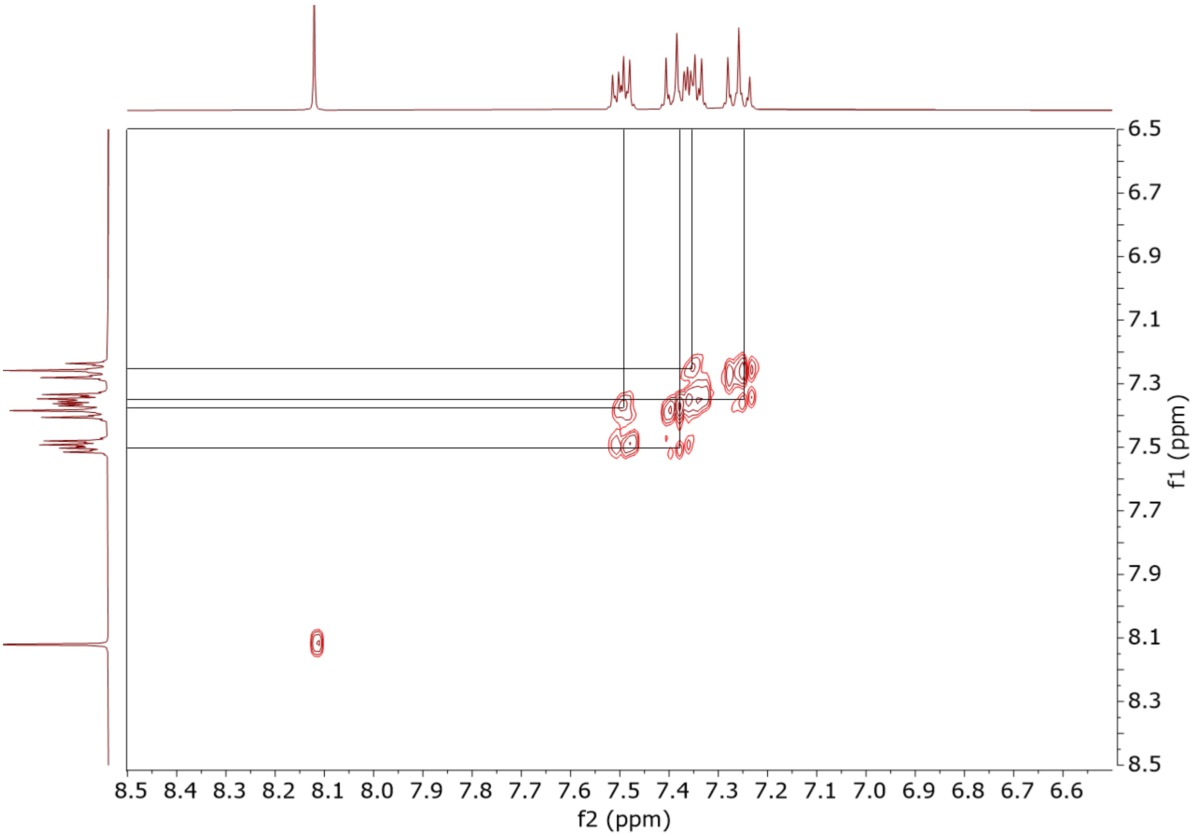

Figure S44: The ^{1}H-^{1}H COSY NMR spectrum of 1,5-bis(4-fluorophenyl)-1H-1,2,3-triazole in DMSO-d_6 at 298 K.

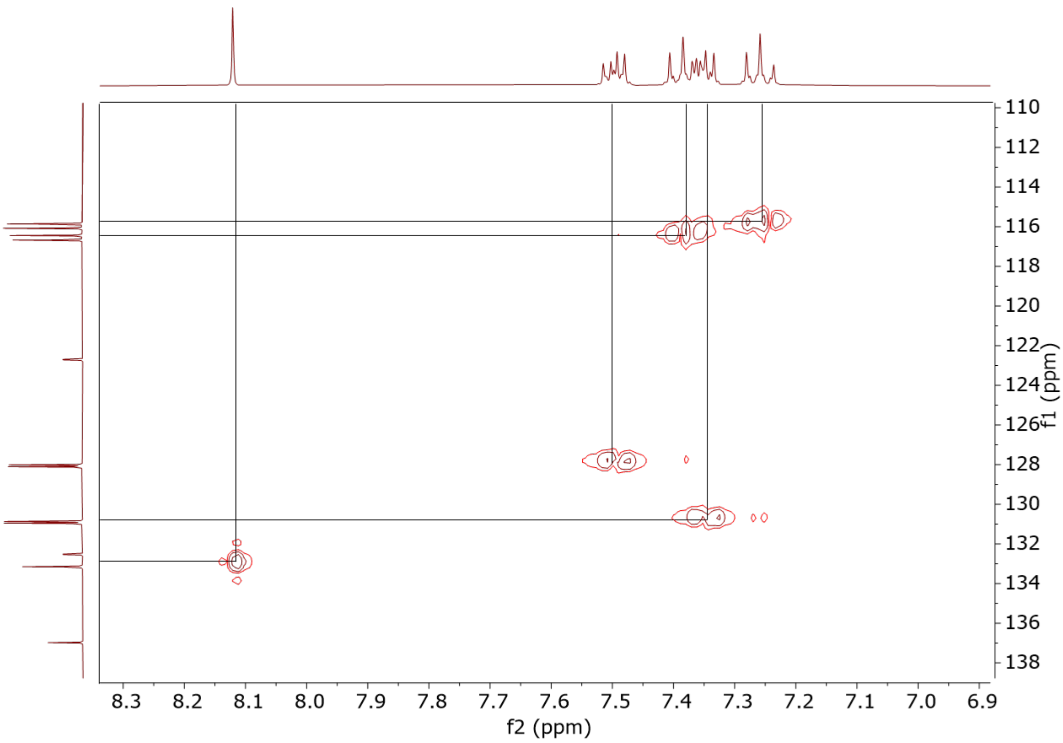

Figure S45: The ^{1}H-^{13}C HSQC NMR spectrum of 1,5-bis(4-fluorophenyl)-1H-1,2,3-triazole in DMSO-d_6 at 298 K.

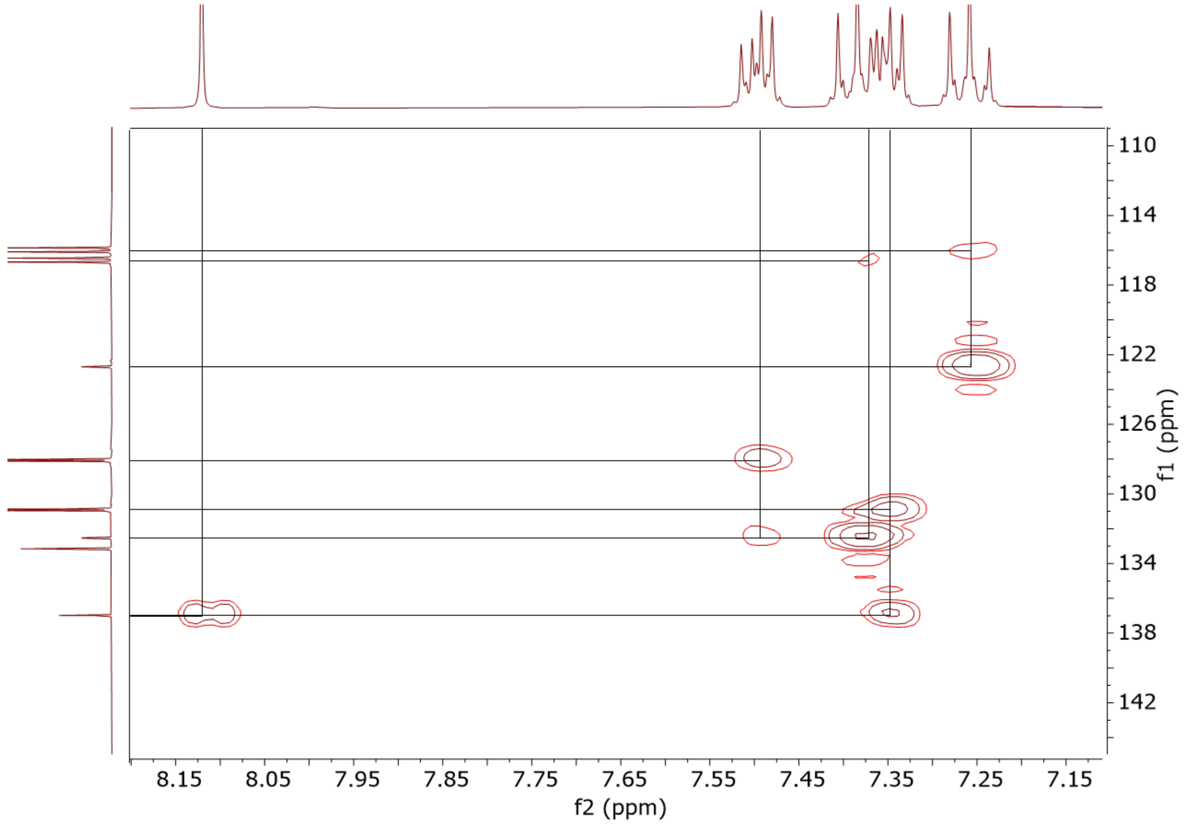

Figure S46: The ^{1}H-^{13}C HMBC NMR spectrum of 1,5-bis(4-fluorophenyl)-1H-1,2,3-triazole in DMSO-d_6 at 298 K.

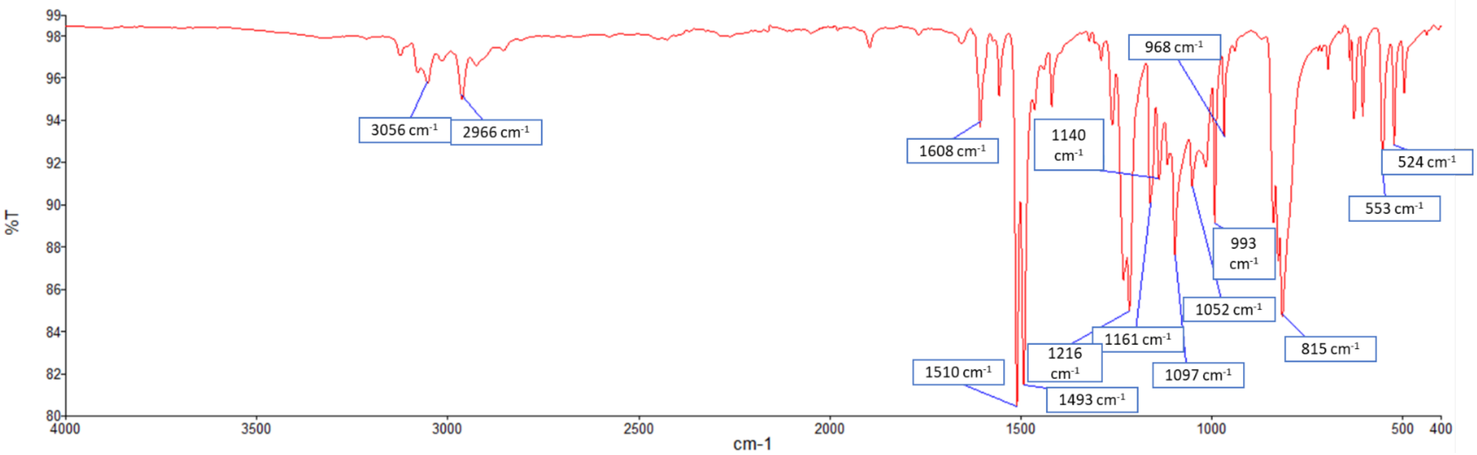

Figure S47: The ATR-IR spectrum of 1,5-bis(4-fluorophenyl)-1H-1,2,3-triazole measured as a solid under air.

Formation of a tricopper(I) acetylide complex

As described in the text above about the synthesis of complex **2**, some batches contain an inseparable byproduct, which was found to be **Cu_3-acetylide** (Scheme S1, Figure 1). This is due to the thermal instability of **2** in alkane solvents. The NMR spectra of a mixture containing **2** and **Cu_3-acetylide** in C_6D_6 at 298 K (Figures S48-S51) showed that **Cu_3-acetylide** is non-symmetric in solution, consistent with the solid-state structure. We observed that heating a hexane solution of complex **2** resulted in a mixture of species (Scheme S1) and we propose **Cu_3-acetylide** is one of them, based on the crystal structure obtained from pentane solution and the resonances observed in the ^{1}H, ^{19}F and ^{31}P NMR spectra that indicate the presence of multiple species (Figures S52-S54).

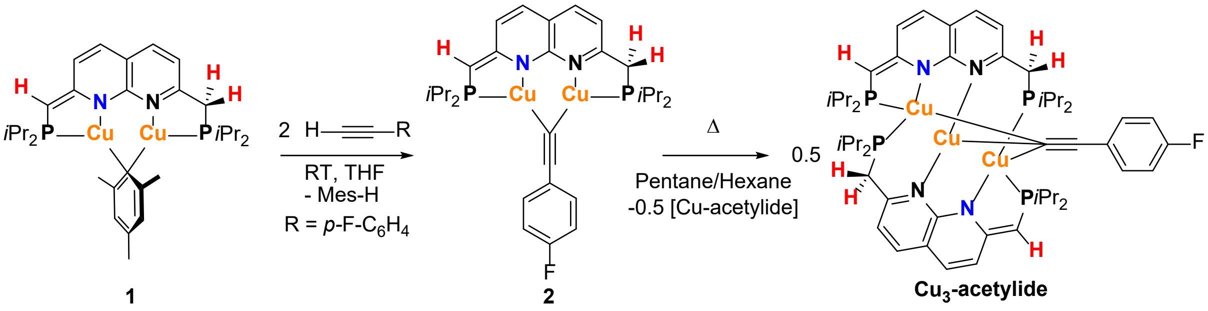

Scheme S1: The synthesis of complex **2** and its decomposition to **Cu_3-acetylide**.

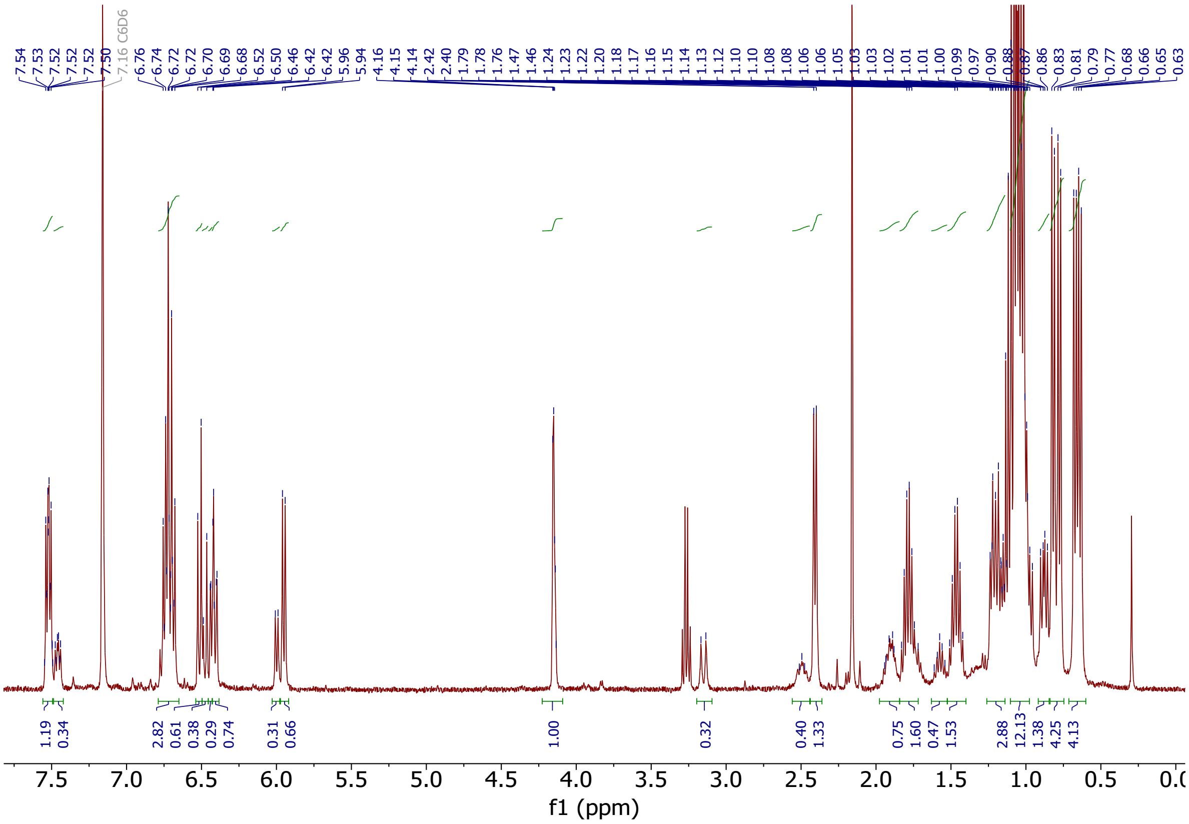

Figure S48: The ^{1}H NMR spectrum of a mixture containing complex **2** and **Cu_3-acetylide** in C_6D_6 at 298 K.

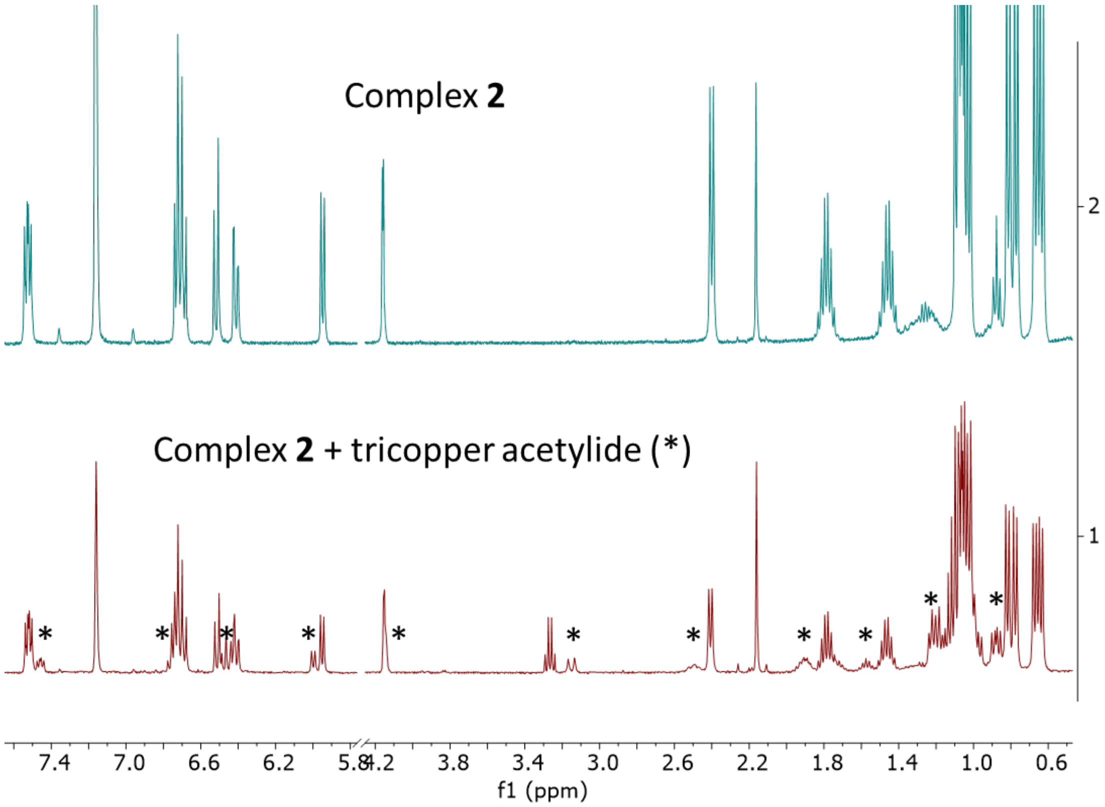

Figure S49: The ^{1}H NMR spectrum of a mixture containing complex **2** and **Cu_3-acetylide** in C_6D_6 at 298 K. The resonances marked with * are assigned to **Cu_3-acetylide**.

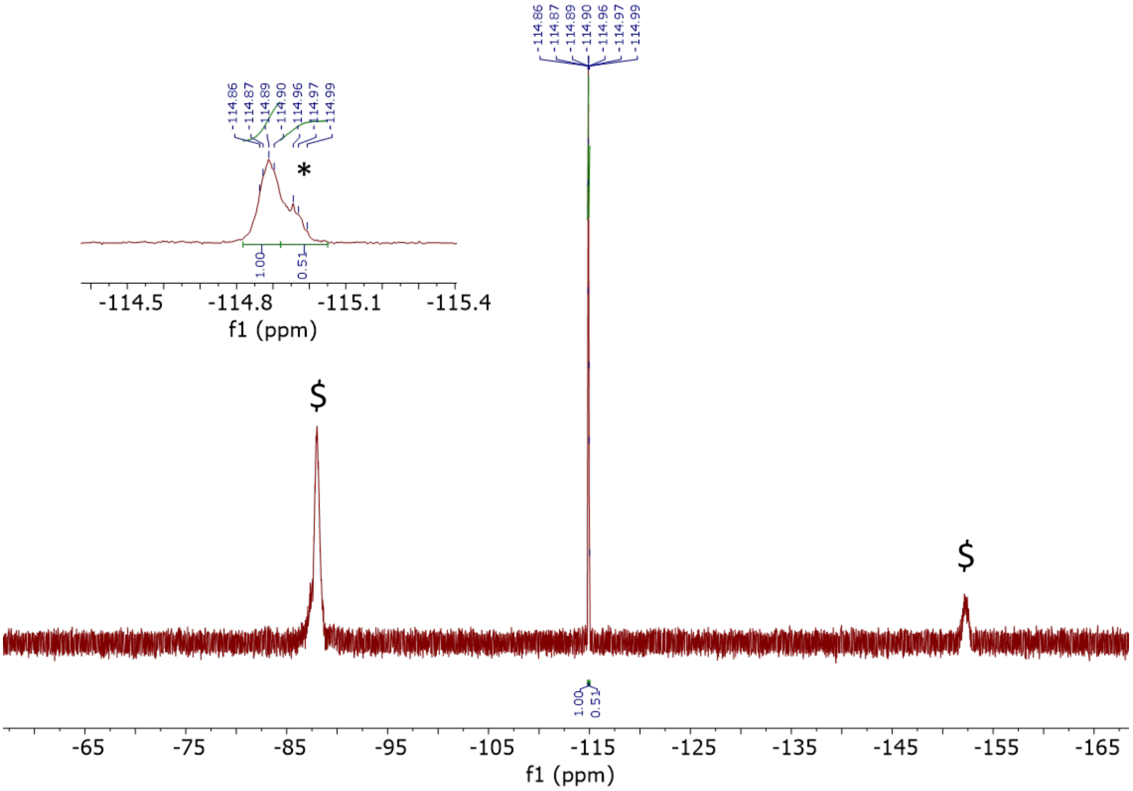

Figure S50: The ^{19}F NMR spectrum of a mixture containing complex **2** and **Cu_3-acetylide** in C_6D_6 at 298 K. The resonances marked with * are assigned to **Cu_3-acetylide** and with $ are artefacts in the probe.

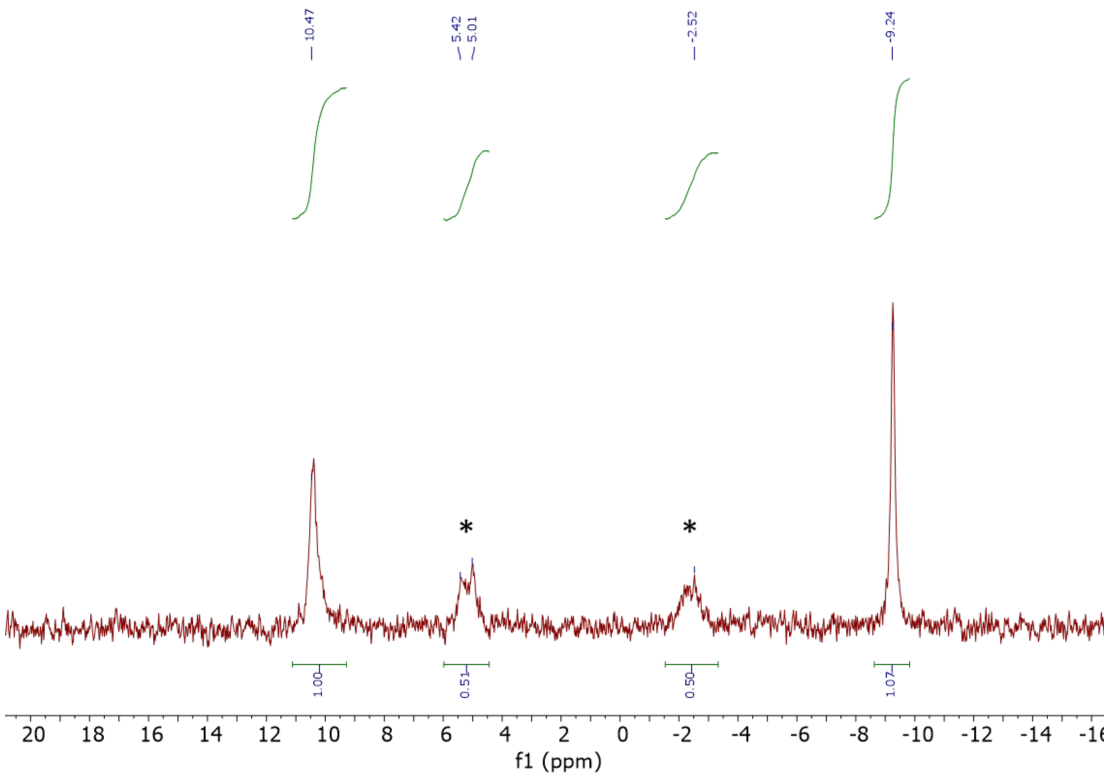

Figure S51: The $^{31}P\{^{1}H\}$ NMR spectrum of a mixture containing complex **2** and **Cu_3-acetylide** in C_6D_6 at 298 K. The resonances marked with * are assigned to **Cu_3-acetylide**.

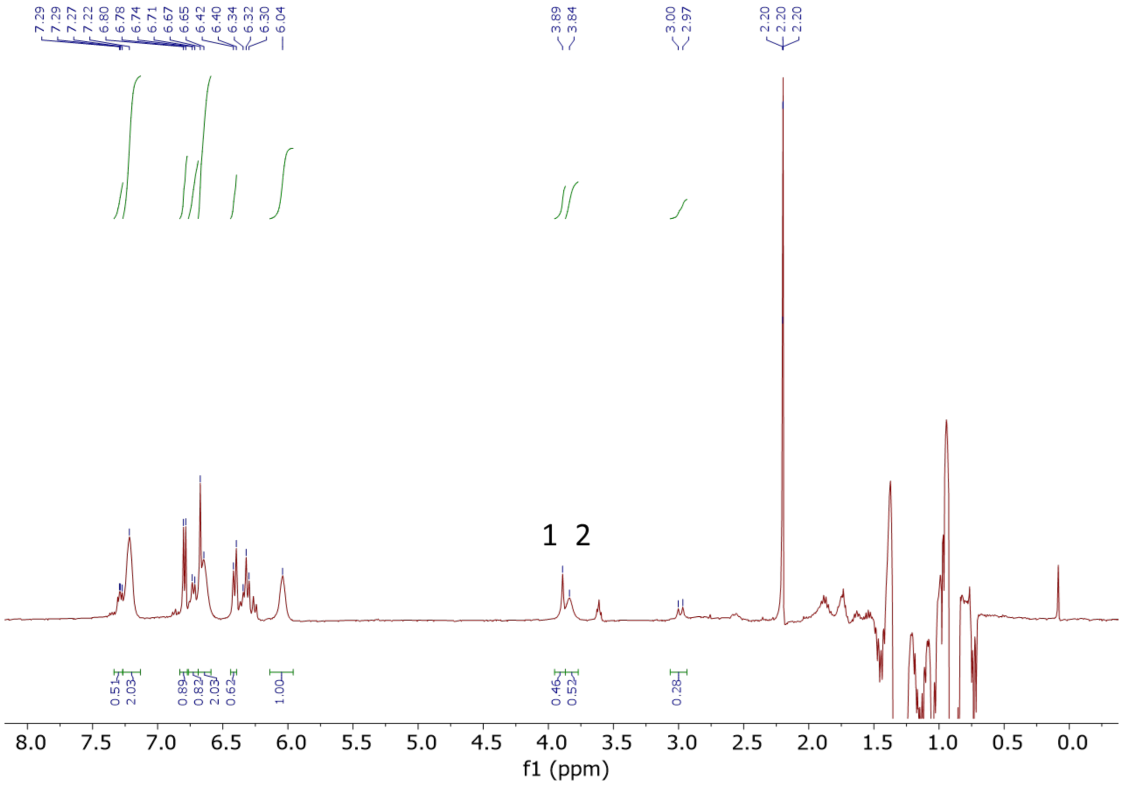

Figure S52: The ^{1}H NMR spectrum, obtained after heating complex **2** at 343 K for 2 h, in hexane at 298 K with solvent suppression (Presat). The resonances indicated with 1 and 2 and the many different resonances observed in the aromatic region are indicative of the presence of at least two compounds, showing that complex **2** is not stable in hexane solution.

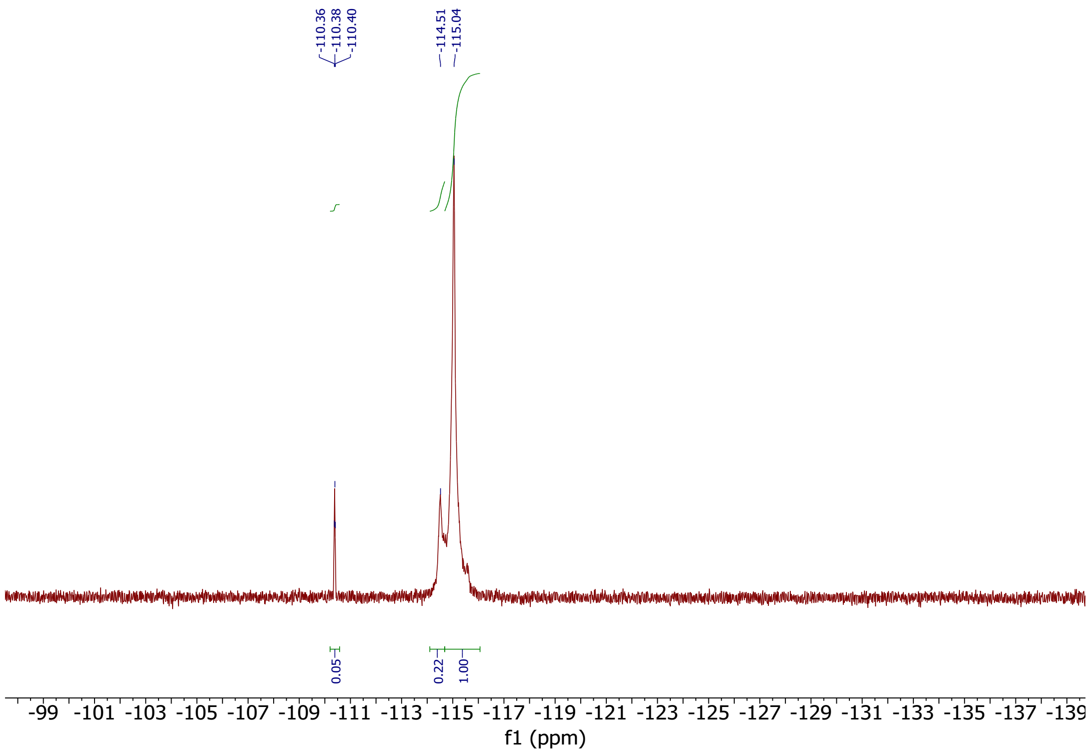

Figure S53: The ^{19}F NMR spectrum, obtained after heating complex **2** at 343 K for 2 h, in hexane at 298 K. The observation of more than one resonance is indicative for the presence of multiple species containing an alkyne/acetylide ligand, showing that complex **2** is not stable in hexane solution.

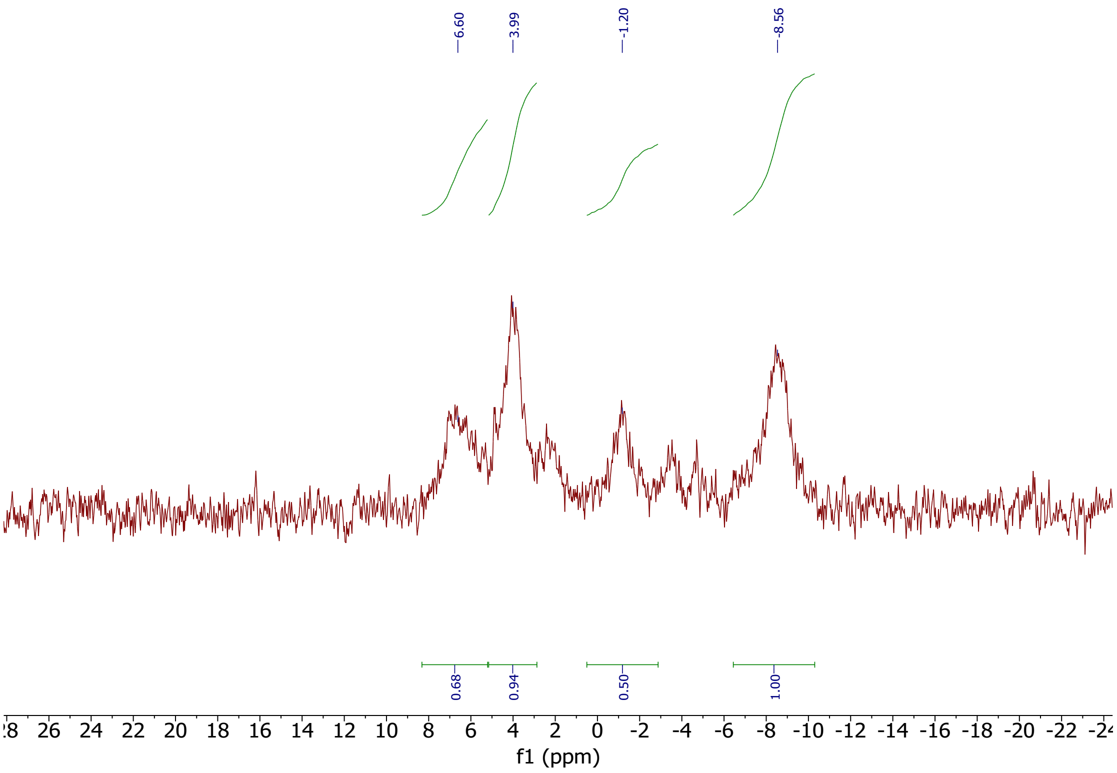

Figure S54: The $^{31}P\{^{1}H\}$ NMR spectrum, obtained after heating complex **2** at 343 K for 2 h, in hexane at 298 K. The observation of multiple broad NMR resonances is a clear indication for the presence of multiple species, showing that complex **2** is not stable in hexane solution.

H/D scrambling in dicopper acetylide complex **2**

Complex **2** (7.0 mg, 11 µmol) dissolved in C_6D_6 (0.60 mL) was placed in a J. Young NMR tube. To the red solution was added 1-ethynyl-4-fluorobenzene-d_1 (1.3 µL, 1.3 mg, 11 µmol) and then mesitylene as internal standard (2.0 µL, 1.7 mg, 14.4 µmol) by microsyringe. The closed NMR tube was then shaken to homogenize the solution. The red solution was monitored by NMR spectroscopy. Over time, a small decrease of the intensity of the methine/methylene linker resonances of complex **2** at δ = 4.16 and 2.40 ppm, respectively, was observed. The intensity of the singlet at δ = 2.65 ppm, assigned to the C^{sp}-H bond of the terminal alkyne, steadily increased during the reaction overnight (18 h). After 18 h, ~25% of the methine and methylene linker protons was exchanged based on integration of these resonances compared to the aromatic naphthyridine resonances that do not exchange. Only one scan was measured for this spectrum using 5 sec of acquisition time to be able to accurately quantify the H/D scrambling. A ^{2}H NMR spectrum was collected after reaction overnight that showed the presence of deuterated alkyne and methylene linker, but the resonances were obscured by the large resonance caused by C_6D_6. Therefore, after 19 h, the red solution was concentrated under a dynamic vacuum and the obtained solid was redissolved in benzene and another ^{2}H NMR spectrum was recorded that showed the presence of deuterated methine and methylene linkers (the volatile alkyne was evaporated), see Figure S57.

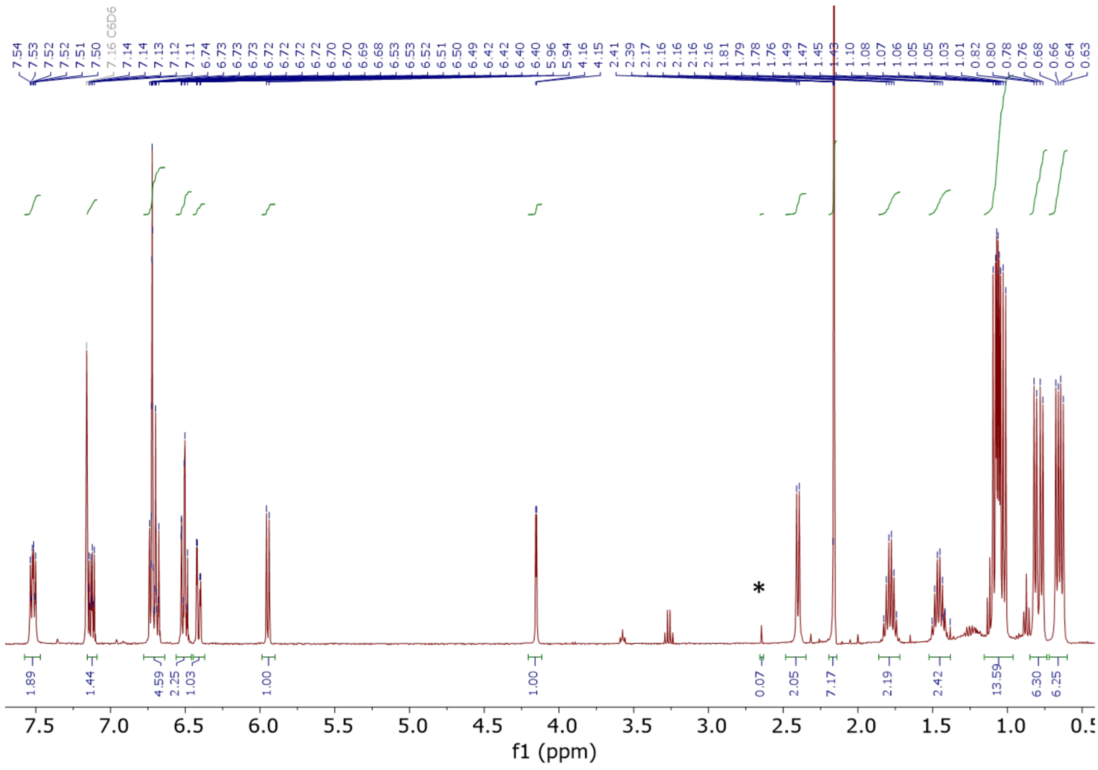

Figure S55: The ^{1}H NMR spectrum of the mixture containing complex **2** and 1-ethynyl-4-fluorobenzene-d_1 in C_6D_6 at 298 K obtained after 1 h. The resonances marked with * is from the alkyne's C^{sp}–H bond.

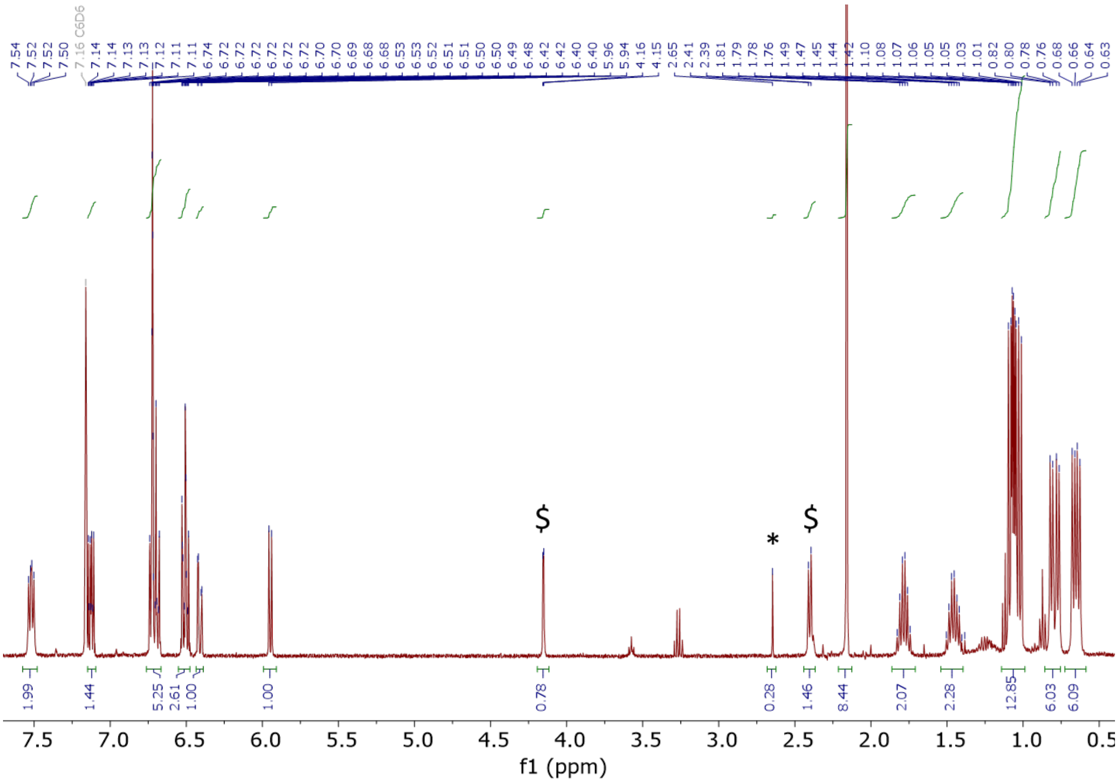

Figure S56: The quantitative ^{1}H NMR spectrum of the mixture containing complex **2** and 1-ethynyl-4-fluorobenzene-d_1 in C_6D_6 at 298 K obtained after 18 h. The resonances marked with * is from the alkyne's C^{sp}–H bond. The resonances indicated by $ are assigned to the methine and methylene linker, which show ~25% decreased intensity.

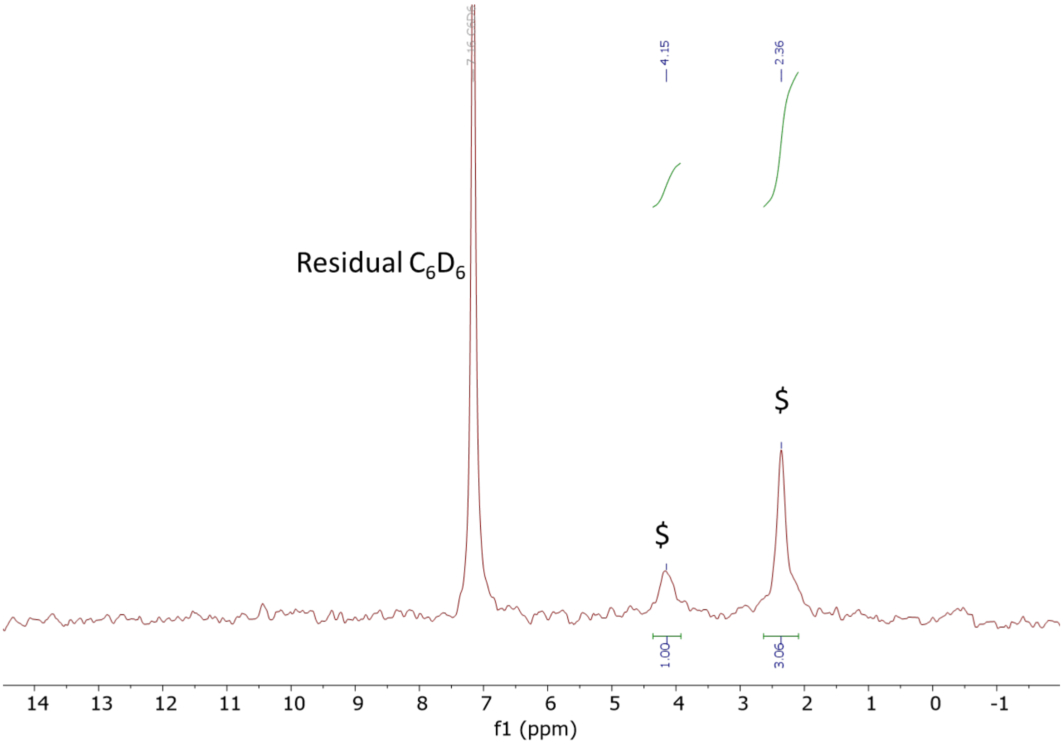

Figure S57: The ^{2}H NMR spectrum obtained after reaction between complex **2** and 1-ethynyl-4-fluorobenzene-d_1 for 19 h at 298 K. The mixture was redissolved in benzene (C_6H_6) after concentrating the mixture under a dynamic vacuum. The volatile alkyne had evaporated. The resonances indicated with $ are assigned to the (partially) deuterated methine and methylene linkers of complex **2**.

Protodemetalation of complex **3** with (deuterated) alkyne

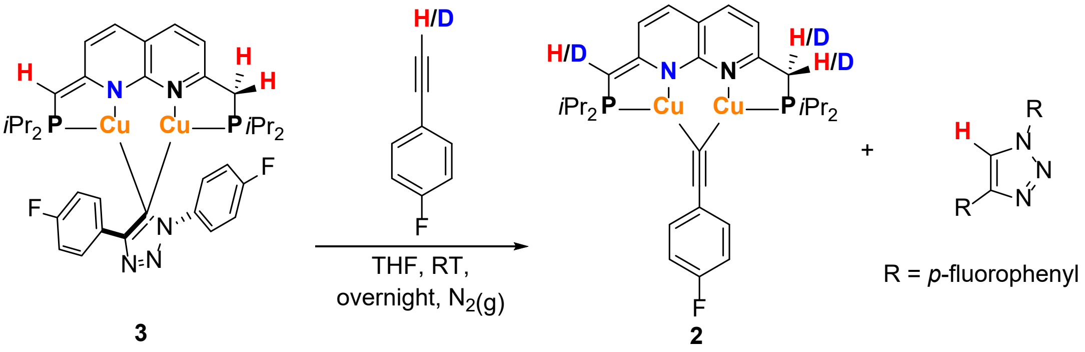

Scheme S2: Protodemetalation of complex **3** using deuterated or non-deuterated 1-ethynyl-4-fluorobenzene.

A solution of complex **3** (24.8 mg, 32.0 µmol) was prepared in C_6D_6 and its purity was determined by NMR spectroscopy. The mixture was quantitatively added to a vial and the mixture was concentrated under a dynamic vacuum. Then, two reactions were performed in parallel using this batch of complex **3**, which was made into a stock solution of complex **3** (9.9 mg, 12.8 µmol) and mesitylene (1.73 mg, 14.4 µmol) per 0.60 mL THF. 0.60 mL of this stock solution was added in two different J. Young NMR tubes. To one tube 1-ethynyl-4-fluorobenzene (1.5 µL, 1.55 mg, 12.8 µmol) was added and to the other tube 1-ethynyl-4-fluorobenzene-d_1 (1.5 µL, 1.55 mg, 12.8 µmol) was added. No color change was observed and both tubes were closed. Next, the solutions were homogenized by shaking the tubes. The mixtures were monitored by NMR spectroscopy and ^{1}H, ^{19}F, ^{31}P{^{1}H} and ^{2}H NMR spectra were collected. For both experiments, the ^{1}H NMR spectra in THF collected at 298 K using the Presat settings for solvent suppression, indicated that a triazole product forms rapidly. Interestingly, for both samples the triazole-H proton at δ = 8.71 ppm (singlet) was observed and only traces of deuterated triazole product were identified in the ^{2}H NMR spectra (see Figure S65).

The deuteration percentage of the triazole was determined by integration of the resonance at δ = 8.71 ppm (1 proton) and comparing it with the resonances overlapping at δ = 7.94 ppm that integrate for 4 protons of the 1,4-triazole product and comparing the ratio between these for both experiments. The experiment without deuterated alkyne gave a ratio between these resonances of 1.16 : 4.00 and the experiment with the deuterated alkyne gave a ratio of 1.09 : 4.00. Dividing the integrals of the resonance at δ = 8.71 ppm for both experiments then gives the percentage of protonated triazole: (1.09/1.16)*100% = 94%. This calculation shows thus that the experiment involving 1-ethynyl-4-fluorobenzene-d_1, results in a triazole product that is 94% protonated; The triazole is 6% deuterated.

The ^{2}H NMR spectra showed in the experiment with 1-ethynyl-4-fluorobenzene-d_1 that the methylene and methine linkers in the product complex **2** are deuterated, which is not observed in the experiment with 1-ethynyl-4-fluorobenzene. After reaction overnight, most of complex **3** is converted to complex **2** and triazole product for both experiments. However, full conversion of the alkyne is observed for both experiments, indicating a substoichiometric addition or the alkyne is partly consumed in an unobserved side reaction.

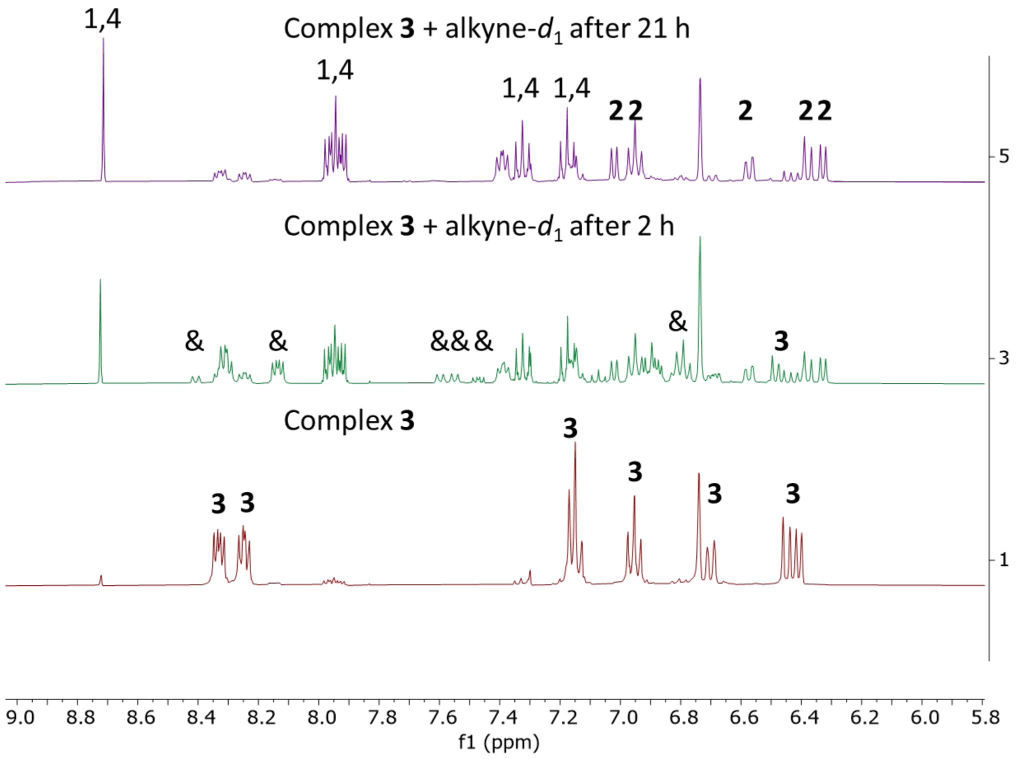

Figure S58: Zoom-in of the aromatic region in the stacked ^{1}H NMR spectra after mixing complex **3** and 1-ethynyl-4-fluorobenzene-d_1 after several time points in THF-h_8 at 298 K with solvent suppression. The resonance at δ = 8.71 ppm is assigned to the triazole (1,4) proton. Complex **2** (**2**) and complex **3** (**3**) and the observed intermediate (&) are also indicated.

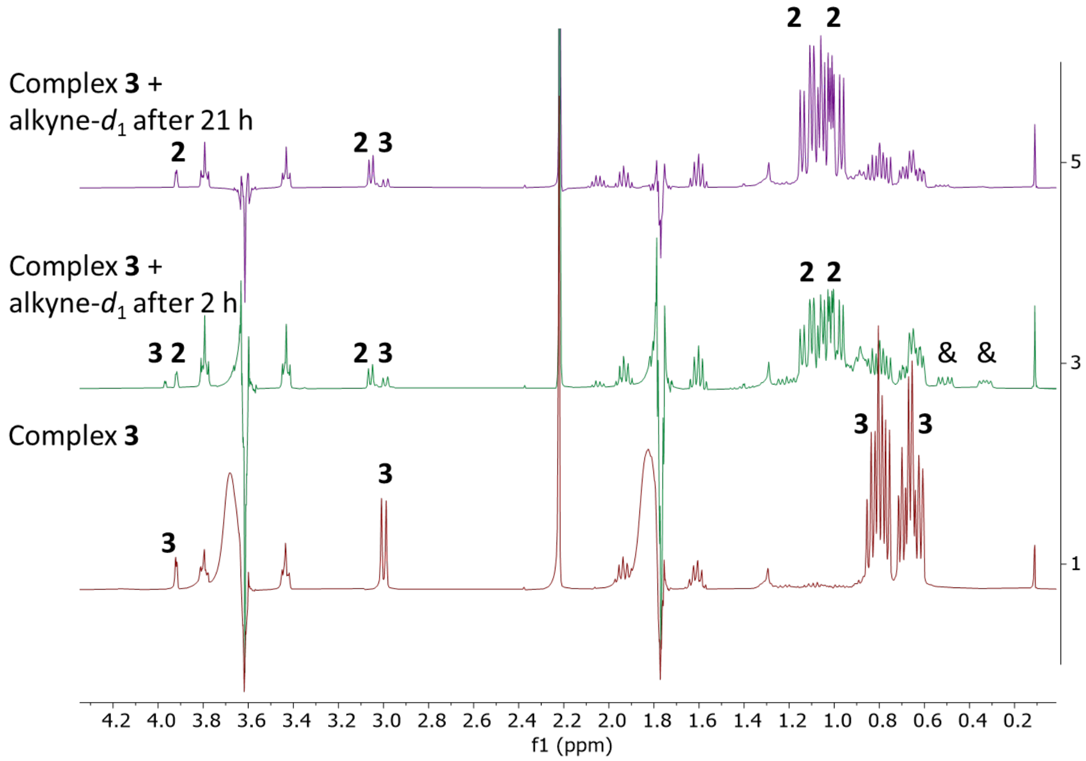

Figure S59: Zoom-in of the aliphatic region in the stacked ^{1}H NMR spectra after mixing complex **3** and 1-ethynyl-4-fluorobenzene-d_1 after several time points in THF-h_8 at 298 K with solvent suppression. Complex **2** (**2**) and complex **3** (**3**) and the observed intermediate (&) are also indicated.

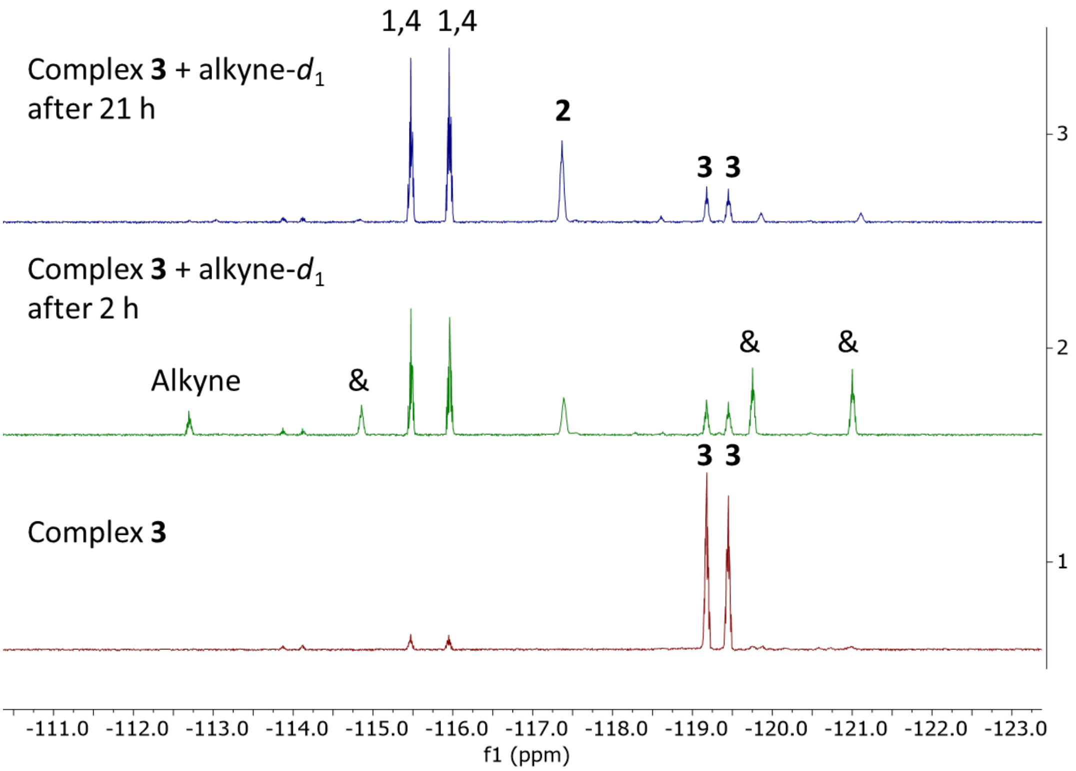

Figure S60: The stacked ^{19}F NMR spectra after mixing complex **3** and 1-ethynyl-4-fluorobenzene-d_1 after several time points in THF-h_8 at 298 K. The resonances at δ = -114.9, -119.8 and -121 ppm observed after 2 h (middle spectrum) are assigned to the observed intermediate (&). Complex **2** (**2**), complex **3** (**3**) and triazole (1,4) are also indicated.

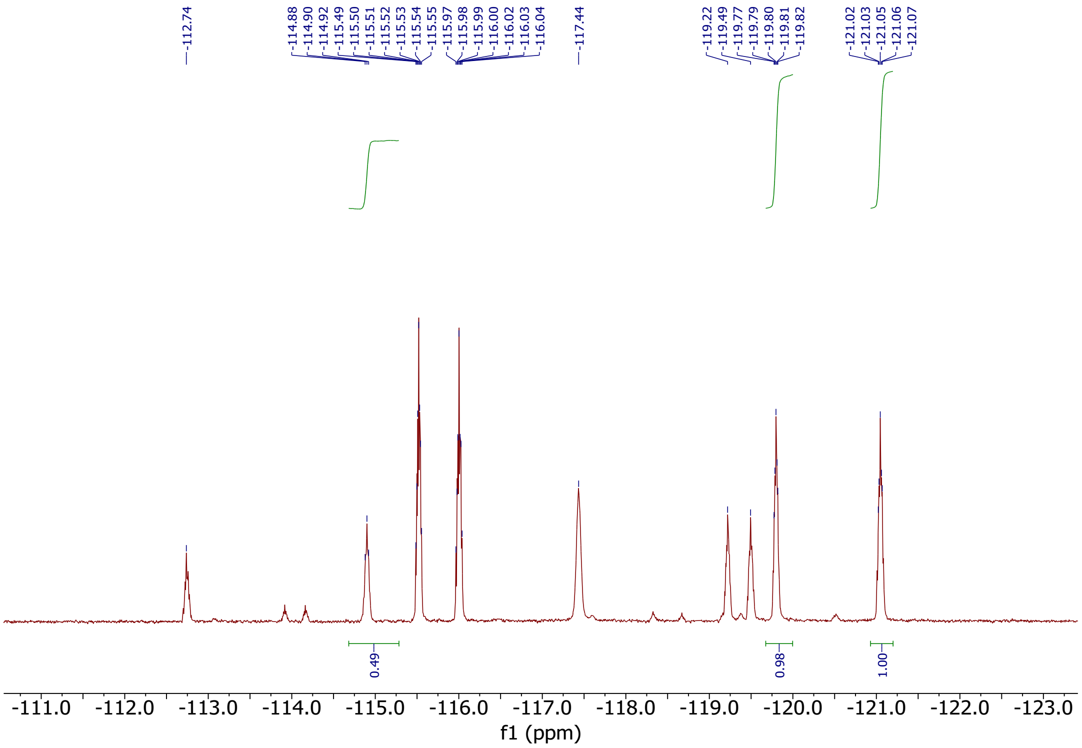

Figure S61: The ^{19}F NMR spectrum obtained 2 h after mixing complex **3** and 1-ethynyl-4-fluorobenzene-d_1 in THF-h_8 at 298 K. The resonances observed at δ = -114.9, -119.8 and -121 ppm are integrated in a 0.5 : 1 : 1 ratio and assigned to the observed intermediate (&).

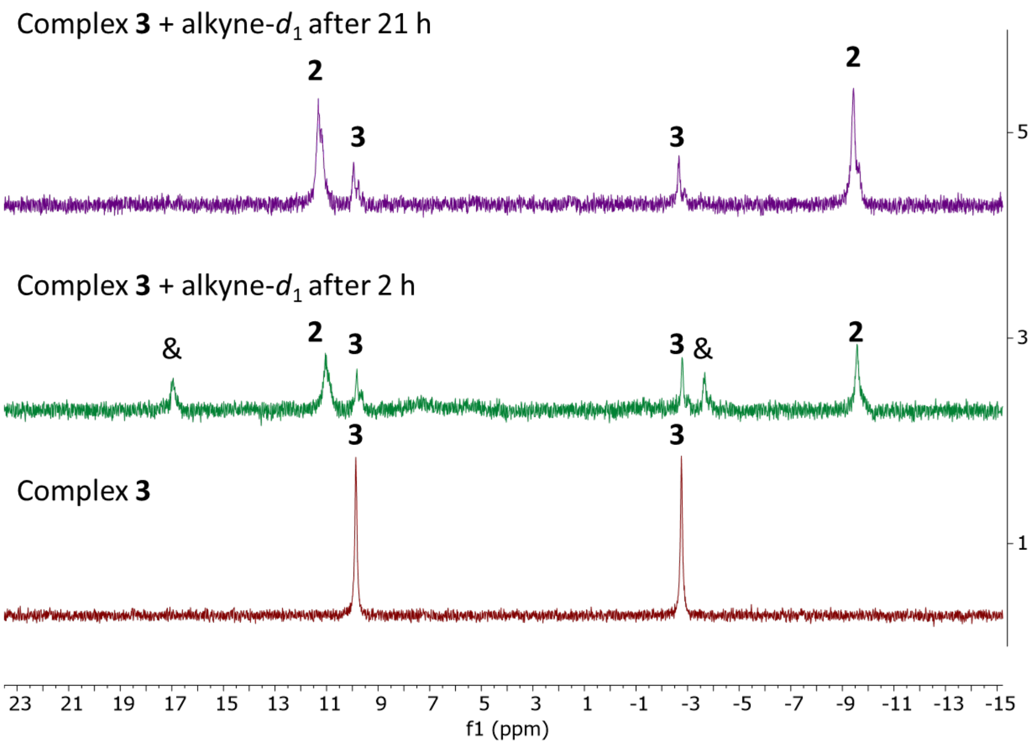

Figure S62: The stacked $^{31}P\{^{1}H\}$ NMR spectra after mixing complex **3** and 1-ethynyl-4-fluorobenzene-d_1 after several time points in THF-h_8 at 298 K. The resonances at δ = 17 and -3.7 ppm observed after 2 h (middle spectrum) are assigned to the observed intermediate (&). Complex **2** (**2**) and complex **3** (**3**) are also indicated.

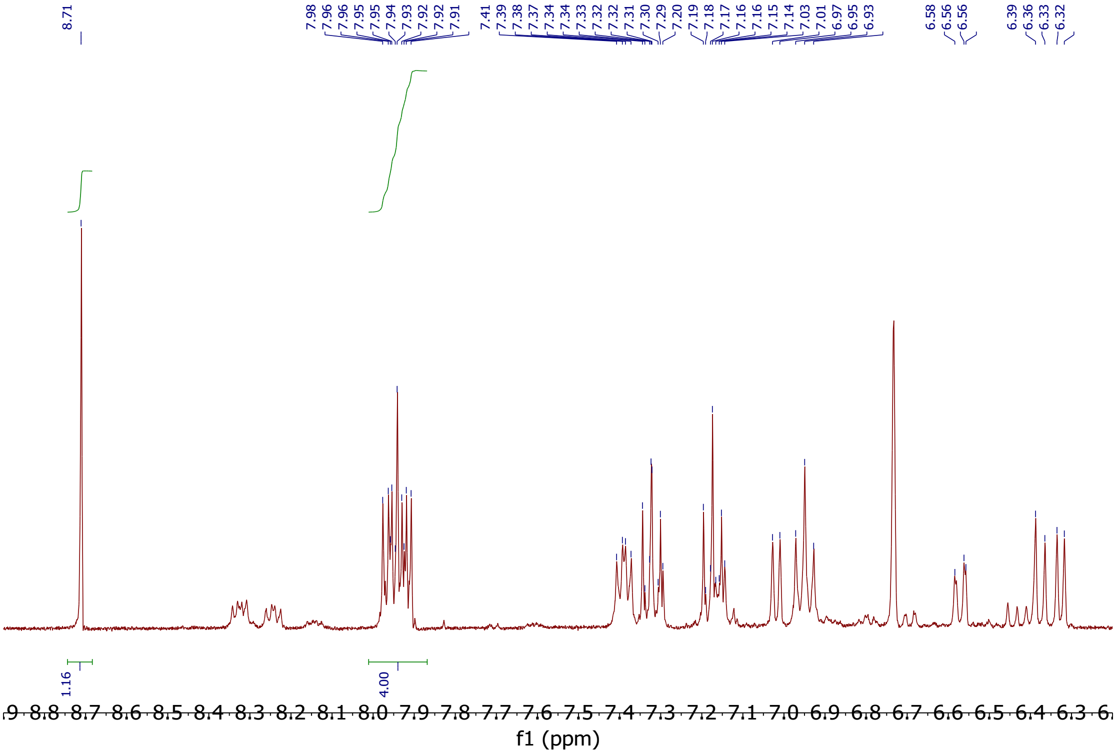

Figure S63: Zoom-in of the aromatic region of the ^{1}H NMR spectrum obtained 21 h after mixing complex **3** and 1-ethynyl-4-fluorobenzene-d_1 in THF-h_8 at 298 K with solvent suppression. The resonance at δ = 8.71 ppm is assigned to the triazole (1,4) proton and its ratio with the resonances at δ = 7.95 ppm (4H) is shown. The ratio close to 1 : 4 indicates that the deuteration percentage is low.

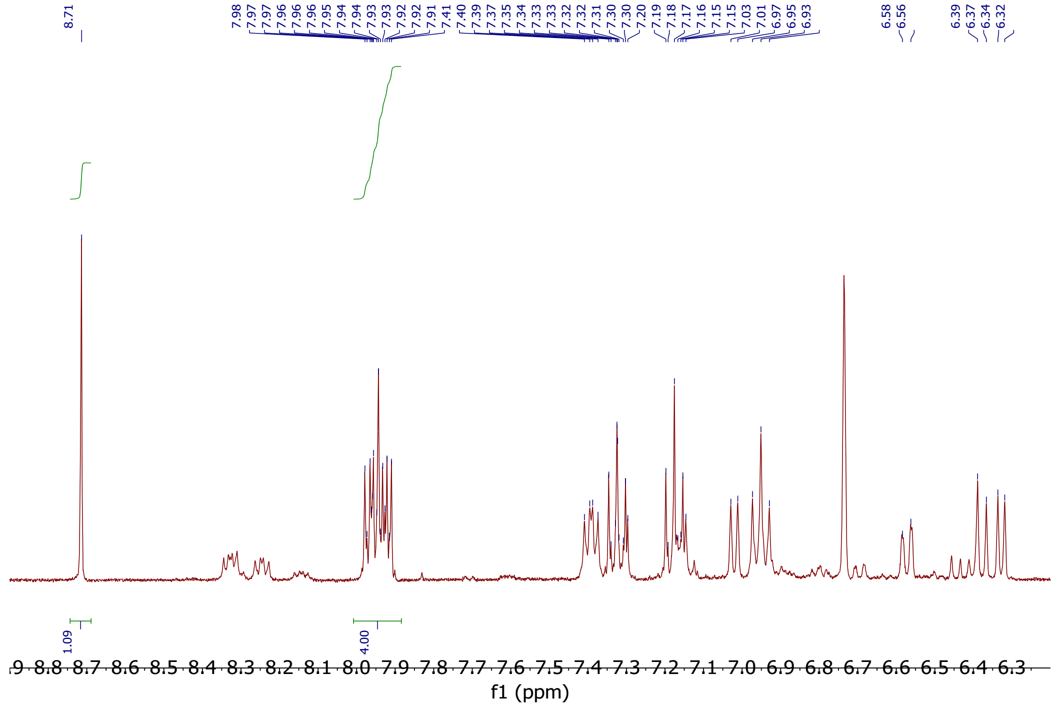

Figure S64: Zoom-in of the aromatic region of the ^{1}H NMR spectrum obtained 21 h after mixing complex **3** and 1-ethynyl-4-fluorobenzene in THF-h_8 at 298 K with solvent suppression. The resonance at δ = 8.71 ppm is assigned to the triazole (1,4) proton and its ratio with the resonances at δ = 7.95 ppm (4H) is shown. The ratio close to 1 : 4 is nearly identical to that in the experiment with the deuterated alkyne in Figure S63.

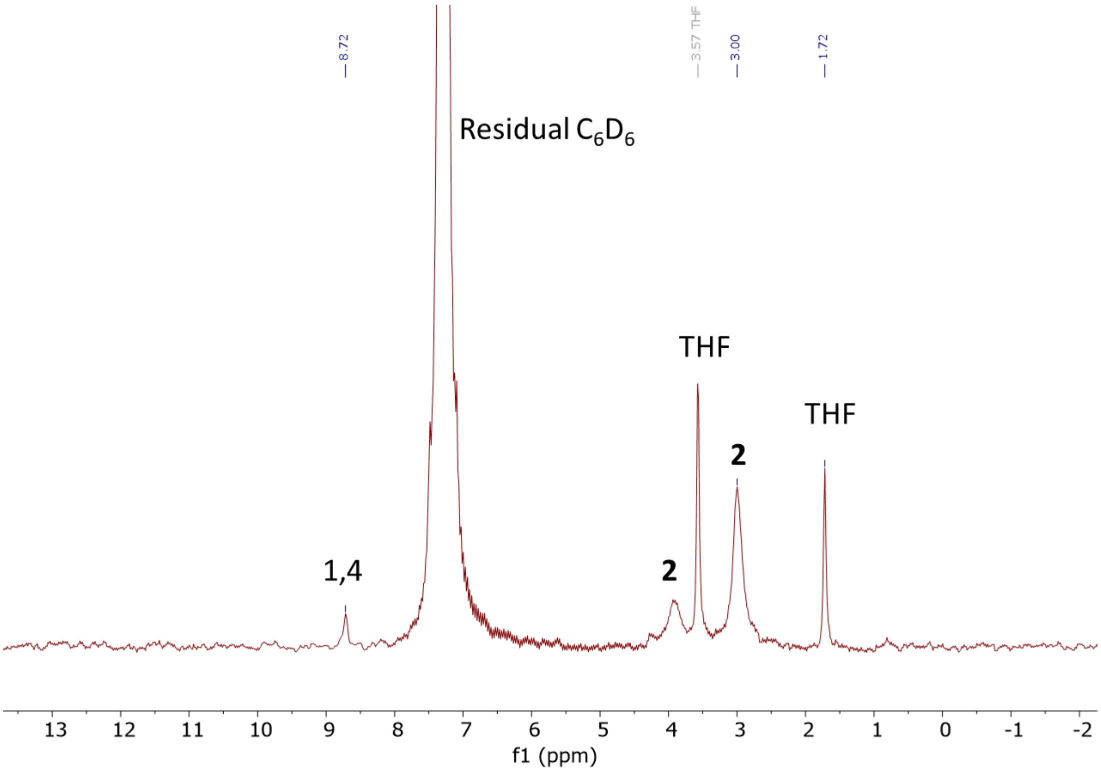

Figure S65: The ^{2}H NMR spectrum obtained 23 h after mixing complex **3** and 1-ethynyl-4-fluorobenzene-d_1 in THF-h_8 at 298 K. The low intensity resonance observed at δ = 8.72 ppm is assigned to the triazole (1,4) proton. The resonances observed at δ = 3.95 and 3.00 ppm indicate that the methine and methylene linker became deuterated in the course of the experiment. Resonances from residual C_6D_6 from the previous analysis and the natural abundance of deuterium in THF are observed.

Crude NMR spectra of the synthesis of complex **3**

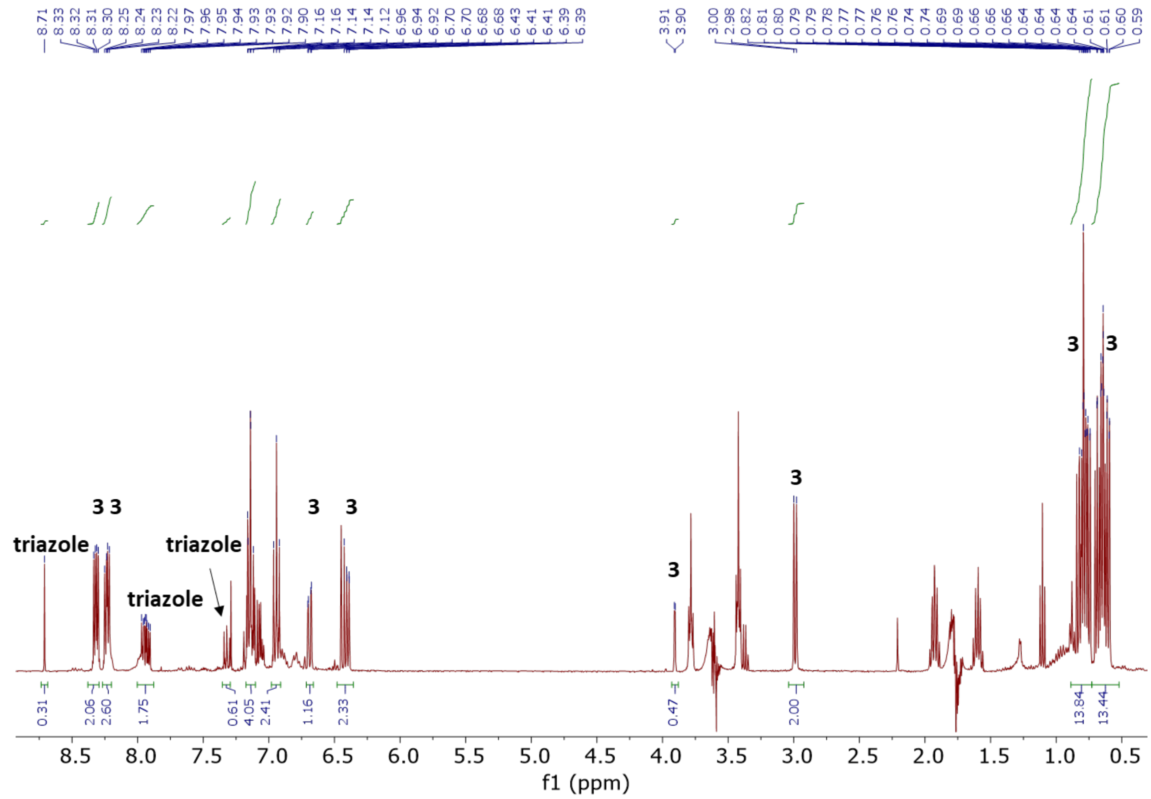

Figure S66: The ^{1}H NMR spectrum of the crude reaction mixture of the synthesis of complex **3** in THF-h_8 at 298 K. The resonances corresponding to complex **3** (**3**) and the 1,4-triazole byproduct (**triazole**) are indicated in the spectrum.

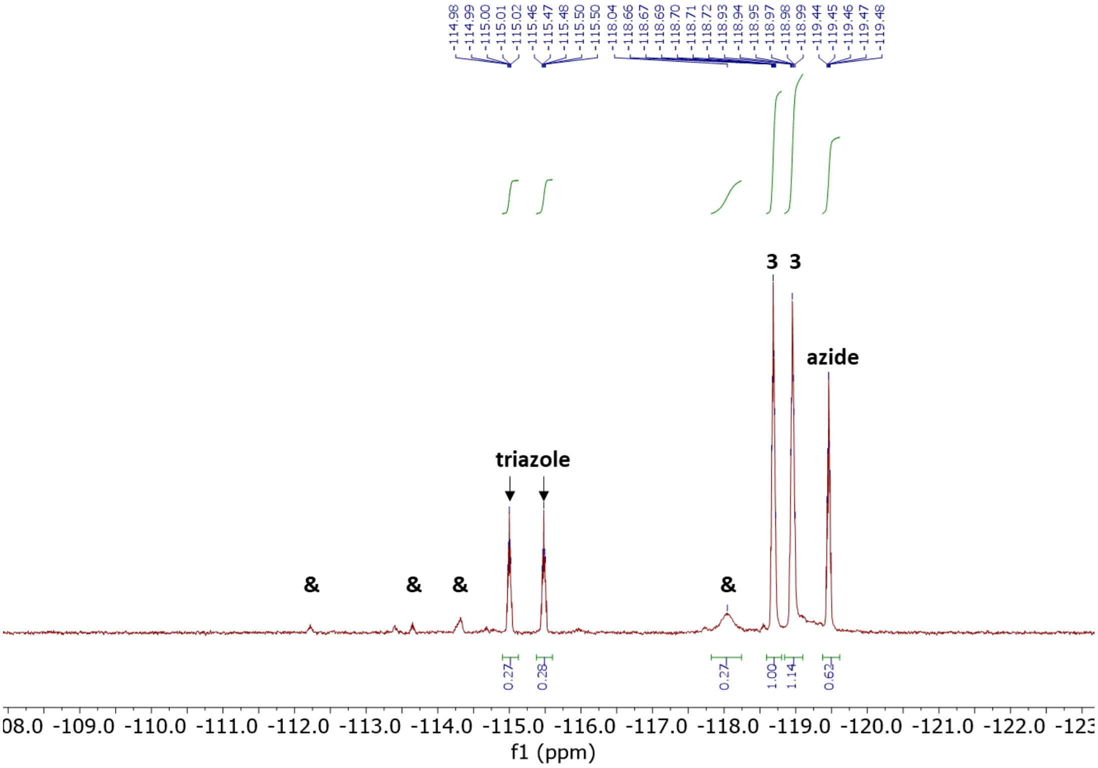

Figure S67: The ^{19}F NMR spectrum of the crude reaction mixture of the synthesis of complex **3** in THF-h_8 at 298 K. The resonances corresponding to complex **3** (**3**), the 1,4-triazole byproduct (**triazole**), azide starting material (**azide**) and unidentified byproducts (**&**) are indicated in the spectrum.

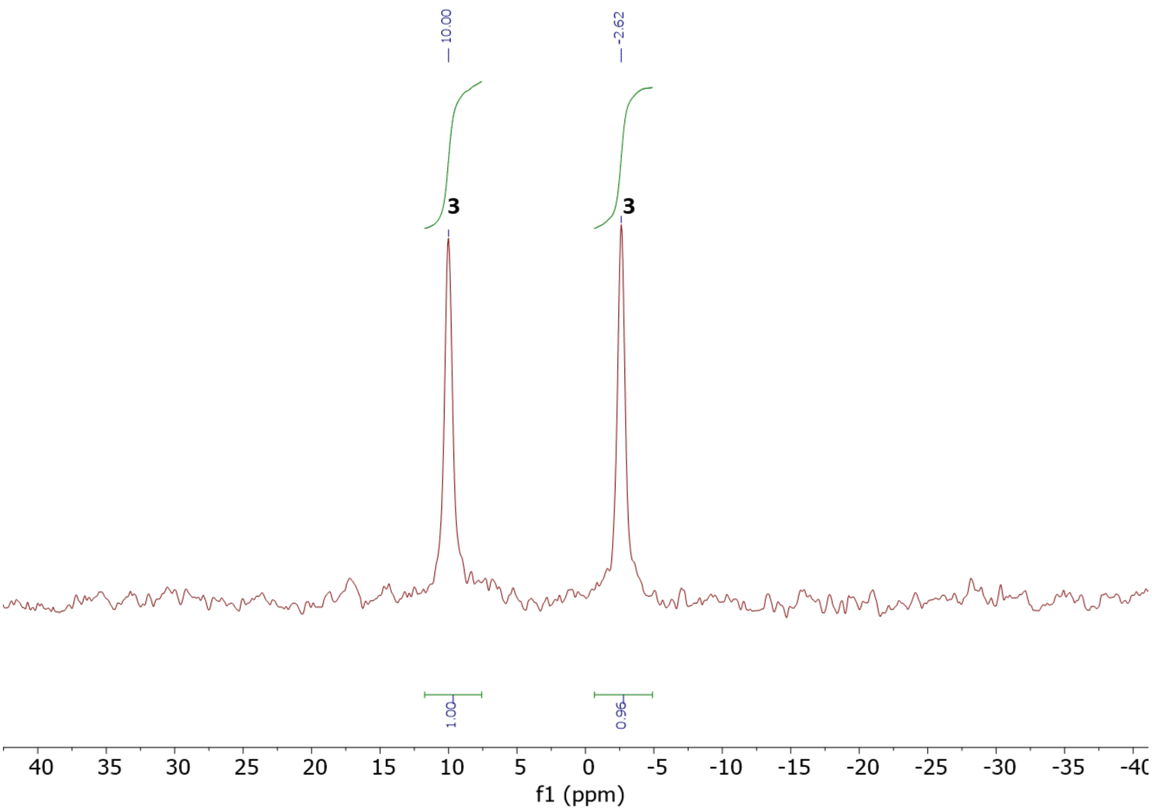

Figure S68: The $^{31}P\{^{1}H\}$ NMR spectrum of the crude reaction mixture of the synthesis of complex **3** in THF-h_8 at 298 K. The resonances corresponding to complex **3** (**3**) are indicated in the spectrum.

CuAAC reaction using 5 mol% complex 2

To an orange-red solution of complex **2** (3.2 mg, 5.0 µmol) in C_6D_6 (0.60 mL) mesitylene (3.0 µL, 2.6 mg, 22 µmol) and 1,2-difluorobenzene (3.0 µL, 3.5 mg, 31 µmol) were added as internal standards. Then, 1-ethynyl-4-fluorobenzene (11.4 µL, 12.0 mg, 100 µmol) and 1-azido-4-fluorobenzene (11.2 µL, 13.7 mg, 100 µmol) were added to the solution. The NMR tube was closed and quickly homogenized and monitored by NMR spectroscopy. After ~20 min, a precipitate started forming in the orange solution. After 20 h, considerable amount of precipitate had formed, which was identified as the 1,4-triazole. The azide was fully consumed and only a few percent of alkyne remained. The 1,4-triazole was observed as sole product.

Crystallographic details

X-ray crystal structure determination of Cu_3-acetylide

$C_{52}H_{74}Cu_3FN_4P_4$ + disordered solvent, Fw = 1088.65[*], red block, 0.26 × 0.18 × 0.13 mm^3, triclinic, $P\overline{1}$ (no. 2), a = 13.6369(9), b = 21.9011(16), c = 23.2832(19) Å, α = 112.211(4), β = 90.523(4), γ = 103.592(4) °, V = 6220.4(8) Å^3, Z = 4, D_x = 1.162 g/cm^3[*], μ = 1.16 mm^{-1}[*]. The diffraction experiment was performed on a Bruker Kappa ApexII diffractometer with sealed tube and Triumph monochromator (λ = 0.71073 Å) at a temperature of 150(2) K up to a resolution of $(\sin \theta/\lambda)_{max}$ = 0.65 Å^{-1}. The Eval15 software[9] was used for the intensity integration. A multi-scan absorption correction and scaling was performed with SADABS[10] (correction range 0.64-0.75). A total of 90397 reflections was measured, 28591 reflections were unique (R_{int} = 0.090), 14734 reflections were observed [$I>2\sigma(I)$]. The structure was solved with Patterson superposition methods using SHELXT.[11] Structure refinement was performed with SHELXL-2018[12] on F^2 of all reflections. The crystal structure contains voids (1284 Å^3 / unit cell) filled with disordered *n*-pentane molecules. Their contribution to the structure factors was secured by back-Fourier transformation using the SQUEEZE algorithm[13] resulting in 271 electrons / unit cell. Non-hydrogen atoms were refined freely with anisotropic displacement parameters. One of the acetylide ligands was refined with a disorder model. Hydrogen atoms were introduced in calculated positions and refined with a riding model. 1258 Parameters were refined with 268 restraints (geometry and displacement parameters of the disordered ligand). R1/wR2 [$I > 2\sigma(I)$]: 0.0491 / 0.0986. R1/wR2 [all refl.]: 0.1091 / 0.1126. S = 0.883. Residual electron density between –0.54 and 0.87 e/Å^3. Geometry calculations and checking for higher symmetry was performed with the PLATON program.[14]

[*] Derived values do not contain the contribution of the disordered solvent molecules.

X-ray crystal structure determination of complex 3

$C_{36}H_{43}Cu_2F_2N_5P_2 \cdot 0.24C_4H_{10}O \cdot 0.76C_5H_{12}$, Fw = 845.39, orange plate, 0.58 × 0.29 × 0.08 mm^3, monoclinic, $P2_1/c$ (no. 14), a = 15.7803(6), b = 12.9509(4), c = 20.5097(7) Å, β = 92.769(2) °, V = 4186.7(2) Å^3, Z = 4, D_x = 1.341 g/cm^3, μ = 1.14 mm^{-1}. The diffraction experiment was performed on a Bruker Kappa ApexII diffractometer with sealed tube and Triumph monochromator (λ = 0.71073 Å) at a temperature of 150(2) K up to a resolution of $(\sin \theta/\lambda)_{max}$ = 0.65 Å^{-1}. The Eval15 software[9] was used for the intensity integration. A numerical absorption correction and scaling was performed with SADABS[10] (correction range 0.65-1.00). A total of 104608 reflections was measured, 9623 reflections were unique (R_{int} = 0.059), 7177 reflections were observed [$I>2\sigma(I)$]. The structure was solved with Patterson superposition methods using SHELXT.[11] Structure refinement was performed with SHELXL-2018[12] on F^2 of all reflections. Non-hydrogen atoms were refined freely with anisotropic displacement parameters. Disorder was present in the solvent region which was partially occupied by diethyl ether (24%) and *n*-pentane (76%). Orientational disorder was found for the five-membered ring of the triazolide ligand. Hydrogen atoms of the metal complex were located in difference Fourier maps. Hydrogen atoms of the disordered solvent molecules were introduced in calculated positions. All hydrogen atoms were refined with a riding model. 526 Parameters were refined with 125 restraints (geometry and displacement parameters of the disordered solvent). R1/wR2 [$I > 2\sigma(I)$]: 0.0308 / 0.0764. R1/wR2 [all refl.]: 0.0494 / 0.0818. S = 1.083. Residual electron density between –0.28 and 0.38 e/Å^3. Geometry calculations and checking for higher symmetry was performed with the PLATON program.[14]

Computational details

All calculations were conducted using DFT[15] as implemented in the Gaussian 09 suite[16] of ab initio quantum chemistry programs with PBE0[17] including Grimme's D3 dispersion correction with Becke-Johnson damping levels of theory.[18] Geometry optimizations proceeded using Pople's 6-31G(d,p) basis set for main group elements.[19] Copper was modeled using Los Alamos effective core potential plus double-ζ basis, LANL2DZ,[20] which includes relativistic effective core potentials. Analytical vibrational frequencies within the harmonic approximation were calculated using the 6-31G(d,p) basis to confirm proper convergence to well-defined minima or saddle points on the potential energy surface. The energies of the optimized structures were reevaluated by additional single-point calculations on each optimized geometry using the same functionals and Pople's 6-311+G(d,p) basis set[21] for main group elements and Stuttgart/Dresden effective core potential, SDD,[22] for copper. Solvation energies were calculated using a solvation model based on density (SMD)[23] at the same level of single-point calculations and were performed with the dielectric constant of $\varepsilon = 7.4257$ for tetrahydrofuran. The Gibbs free energies in solution phase G(sol) were computed with the following equations.

$$G(sol) = E(sol) + G(corr) \quad (1)$$

$$G(corr) = H(corr) - TS(tot) \quad (2)$$

$$H(corr) = E(tot) + k_BT \quad (3)$$

$$E(tot) = E(t)+E(r)+E(v)+E(e) \quad (4)$$

$$S(tot) = S(t)+S(r)+S(v)+S(e) \quad (5)$$

$$\Delta E(SCF) = \Sigma E(SCF) \text{ for products} - \Sigma E(SCF) \text{ for reactants} \quad (6)$$

$$\Delta G(sol) = \Sigma G(sol) \text{ for products} - \Sigma G(sol) \text{ for reactants} \quad (7)$$

E(sol) is the electronic energy in solution phase computed from the SCF (self-consistent field) procedure with the SMD calculations; G(sol) is the free energy in solution phase; G(corr) is the thermal correction to the free energy; T is the temperature (298.15 K); S(tot) is the entropy; E(tot) is the total internal thermal energy; E(t), E(r), E(v), and E(e) are the internal thermal energies from translation, rotation, vibration, and electronic motions, respectively; S(t), S(r), S(v), and S(e) are the entropies from translation, rotation, vibration, and electronic motions, respectively; The entropy we refer to is specifically of the solute(s), and the entropy of the solvent is implicitly comprised in the continuum solvation model.

Supplementary calculations

Benchmark study

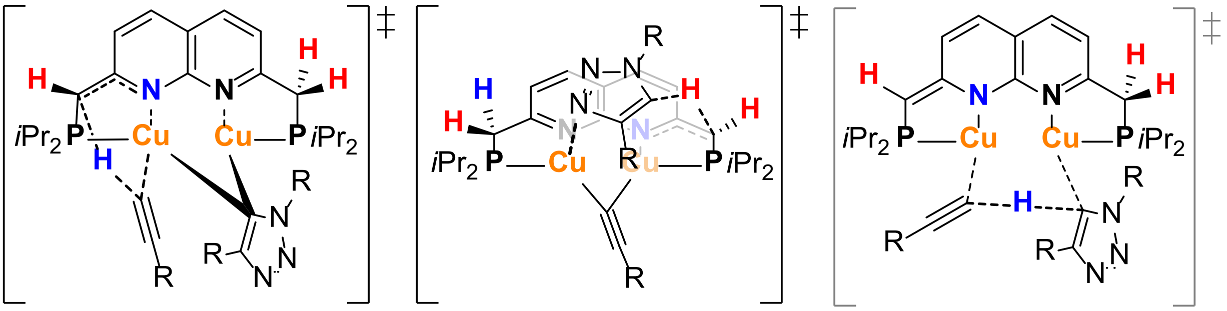

3-TS **4-TS** **4'-TS**

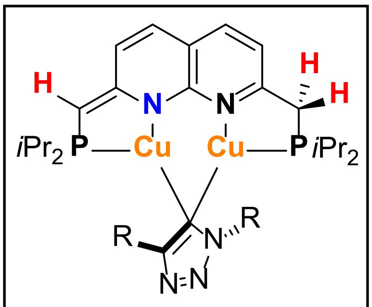

3
(0.00)

ΔG(sol) (kcal/mol)	3-TS	4-TS	4'-TS
**B3LYP-D3**	31.74	30.04	38.80
**M06**	30.49	28.57	-
**PBE0-D3BJ**	24.33	21.22	31.43

(R = *p*-F-C_6H_4)

Table S1: Comparison of DFT functionals for the MLC (**3-TS** and **4-TS**) and direct (**4′-TS**) proton transfer pathways.

To select an appropriate DFT functional for our system, we performed a benchmark study comparing B3LYP-D3, M06, and PBE0-D3(BJ) using the same basis sets [6-311+G(d,p) for main group elements, SDD for Cu, and 6-31G(d,p)/LANL2DZ for geometry optimization].

Complex **3** was used as the reference state, consistent with the main text. All functionals predicted the same qualitative trend: the MLC proton transfer pathway (**3-TS** and **4-TS**) is more favorable than the direct proton transfer pathway (**4′-TS**). However, the computed barriers varied substantially. B3LYP-D3 and M06 significantly overestimated the activation barrier for **3-TS** (>30 kcal/mol), inconsistent with the observed reactivity under the experimental conditions. In contrast, PBE0-D3(BJ) yielded barrier heights within a reasonable energetic window while preserving the correct pathway preference. Notably, similar choices have been reported for CuAAC reactions in dicopper complexes, further supporting the suitability of PBE0-D3(BJ) for the present study. PBE0-D3 was also selected in a previous study by Tilley and co-workers for its ability to reproduce both the bond lengths observed in the X-ray crystal structure and the corresponding barrier heights.[24] Therefore, PBE0-D3(BJ) was adopted for all reported calculations in the main text.

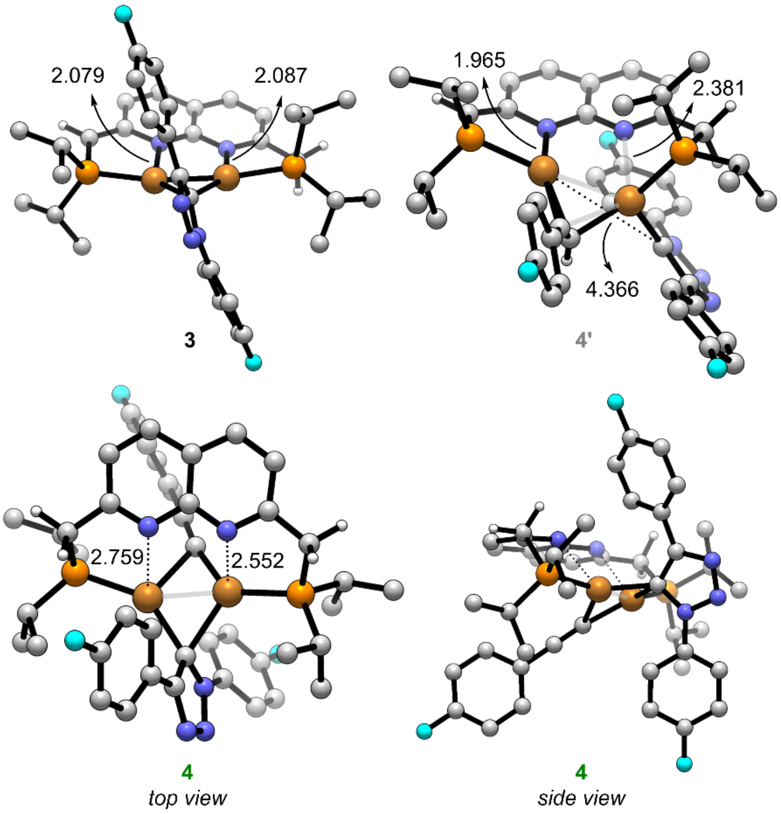

Figure S69: DFT-optimized structures of **3**, **4'**, and **4** (top and side view). All bond lengths are represented in Å. Unnecessary hydrogen atoms are omitted for clarity.

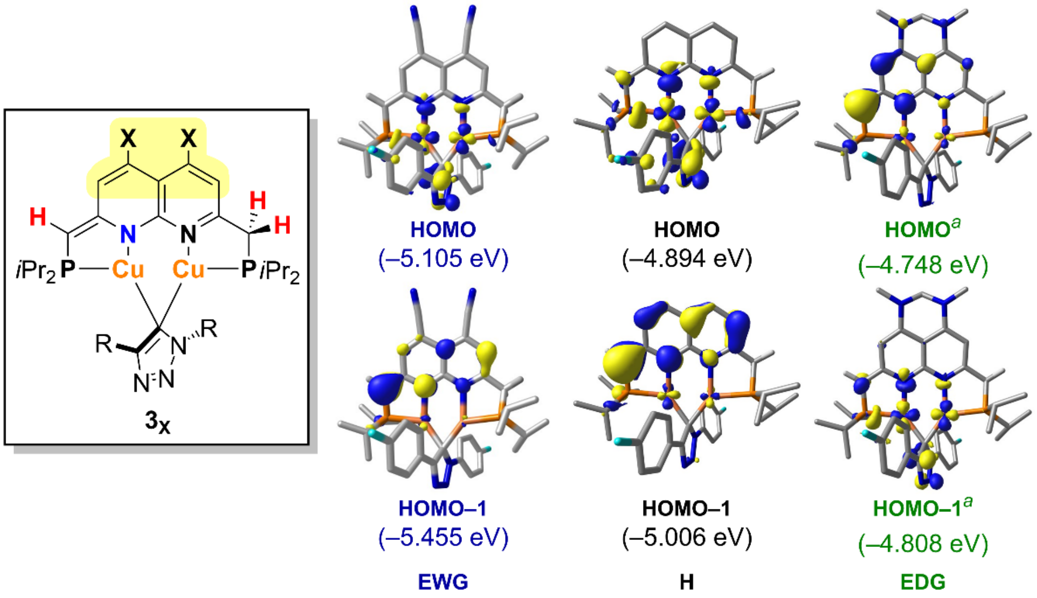

ΔG(sol) (kcal/mol)	X X	3_x-TS	4_x	4_x'-TS	ΔE(SCF) (4_x'-TS – 3_x-TS)
**EWG**	CN CN	27.95	0.88	31.75	0.81
**H**	H H	24.33	–4.17	31.43	4.84
**EDG**	N N (1,3-dimethyl-2,2-dihydroperimidine)	23.20	–6.92	34.06	5.47

Figure S70: Calculated energies of intermediates and transition states related to proton transfer steps (table) with varying substituents (**X**) on the naphthyridine backbone (top left). The relative free energies are with respect to $\mathbf{3_X}$. Top right: Molecular orbital structures (isodensity = 0.05 a.u.) and electronic energies of molecular orbitals **HOMO–1** and **HOMO** of $\mathbf{3_X}$. MOs and their energies were calculated by PBE0-D3(BJ)/6-311+G(d,p)|SSD level of theory. Unnecessary hydrogen atoms are omitted for clarity. [a]The energy levels of MOs between **HOMO–1** and **HOMO** are inverted, compared to the original complex ($\mathbf{3_H}$).

In the main text, we discussed the potential of both the methine carbon in the ^{iPr}**PNNP*** and bridged triazolyl carbon of **3** to serve as proton acceptors from the **alkyne**. This proton transfer can proceed either through a metal ligand cooperative (MLC) pathway via **3-TS** or direct alkyne-to-triazolide proton transfer via **4'-TS**.

To better understand the correlation between the rate of the proton transfer and the donating ability of the ^{iPr}**PNNP*** ligand, we performed a series of *in-silico* experiments. We introduced an electron-withdrawing cyano group ($\mathbf{3_{EWG}}$) and an electron-donating 1,3-dimethyl-2,2-dihydroperimidine group ($\mathbf{3_{EDG}}$) at the two para-positions of the naphthyridine backbone. We hypothesized that the basicity of the methine carbon in the PNNP ligand would directly influence the rate of the MLC proton transfer.

Figure S70 illustrates molecular orbital energies and corresponding orbital structures of $\mathbf{3_X}$ (**X** = **EWG**, **H**, and **EDG**), along with the relative free energies of $\mathbf{3_X}$**-TS**, $\mathbf{4_X}$ and $\mathbf{4_X}$**'-TS**. We first analyzed the

molecular orbitals of $\mathbf{3_X}$ in greater detail, to gain insight into the basicity of the methine and triazolyl carbons. In the case of $\mathbf{3_H}$ (identical to **3** in main text), the π-MO of naphthyridine in ^{iPr}**PNNP*** dominates **HOMO–1** (–5.01 eV), while the bridged triazolide carbon contributes mainly to the **HOMO** (–4.89 eV). The small energy gap of 0.11 eV suggests that both sites have comparable proton-accepting ability. Similarly, the **HOMO–1** and **HOMO** orbitals of $\mathbf{3_{EWG}}$ closely resemble those of $\mathbf{3_H}$. Notably, the electron-withdrawing cyano group stabilizes **HOMO–1** more than **HOMO**, resulting in a larger energy difference of 0.35 eV compared to $\mathbf{3_H}$. Conversely, in $\mathbf{3_{EDG}}$, the electron-donating group destabilizes the ligand's π-MO, inverting the energy levels of **HOMO–1** and **HOMO**, relative to $\mathbf{3_H}$. These results suggest that the donating ability of the ligand directly influences the energy level of the ligand's π-MO in $\mathbf{3_X}$ and, consequently, the basicity of the methine carbon.

We also compared the activation barriers of MLC proton transfer ($\mathbf{3_X}$**-TS**) and direct alkyne-to-triazolide proton transfer ($\mathbf{4_X}$**'-TS**). As shown in the table (Figure S70), the calculated barriers for $\mathbf{3_X}$**-TS**, as well as the energies of intermediates $\mathbf{4_X}$, gradually decrease with increasing electron density on the ligand backbone. This trend aligns with the anticipated increase in basicity for more electron-rich ligands. In contrast, the barrier for the direct alkyne-to-triazolide proton transfer via $\mathbf{4_X}$**'-TS** remains nearly unchanged between $\mathbf{4_{EWG}}$**'-TS** and $\mathbf{4_H}$**'-TS**, while $\mathbf{4_{EDG}}$**'-TS** exhibits a higher barrier by approximately 3 kcal/mol.

To isolate the electronic effects of the substituents, we analyzed the electronic energy gap (ΔE(SCF)) between $\mathbf{4_X}$**'-TS** and $\mathbf{3_X}$**-TS**. A larger ΔE(SCF) value indicates a greater preference for the MLC pathway over the direct transfer. The observed trend (ΔE(SCF): **EWG** < **H** < **EDG**) supports the hypothesis that electron-donating substituents promote the MLC pathway by stabilizing its transition state as a result of the enhanced basicity of the ligand.

Although this electronic effect modulates the barrier difference between the two transition states, the MLC pathway remains preferred in all cases. This result highlights that the structural distortion in $\mathbf{4_X}$**'-TS** still plays a significant role in its destabilization, as discussed in the main text.

Energy components for optimized structures

Table S2: Energy components for DFT-optimized structures

	**E(SCF)(SCF)** (eV)	**E(sol)(SCF)** (eV)	**Thermal Correction to G(corr)** (eV)
	**PBE0-D3(BJ) /6-311++G(d,p)|SDD**	**PBE0-D3(BJ) /6-311++G(d,p)|SDD**	**PBE0-D3(BJ) /6-31G(d,p)|LANL2DZ**
**Azide**	–	–13460.739	1.73
**Alkyne**	–11082.726	–11082.984	1.92
**triazole**	–	–24547.218	4.42
**2**	–	–66722.475	14.87
**2-TS**	–	–80183.264	17.25
**3**	–80185.423	–80186.708	17.53
**3-TS**	–91267.705	–91269.176	19.99
**3-TS$_{alkyne}$**	–11080.943	–	–
**3-TS$_3$**	–80184.543	–	–
**4**	–	–91270.499	20.08
**4-TS**	–	–91269.345	20.02
**4’**	–	–91269.587	20.09
**4’-TS**	–91267.495	–91268.84	19.96
**4’-TS$_{alkyne}$**	–11081.554	–	–
**4’-TS$_3$**	–80183.352	–	–
**2+triazole**	–	–91270.547	20.11
**3$_{EWG}$**	–	–85202.375	17.26
**3$_{EWG}$-TS**	–96283.002	–96284.647	19.69
**4$_{EWG}$**	–	–96286.032	19.90
**4$_{EWG}$’-TS**	–96282.967	-96284.538	19.74
**3$_{EDG}$**	–	–86370.796	19.99
**3$_{EDG}$-TS**	–97451.665	–97453.352	22.49
**4$_{EDG}$**	–	–97454.764	22.60
**4$_{EDG}$’-TS**	–97451.428	-97452.897	22.51

Vibrational frequencies of optimized structures

Table S3: Vibrational frequencies (in cm^{-1}) of optimized structures

================================
azide
================================
63.30 130.05 166.84 337.75 376.33 422.00 429.51
448.28 521.55 556.27 647.53 652.30 712.07 794.45
826.32 854.93 881.39 947.85 966.99 1033.59 1126.35
1181.65 1193.44 1308.77 1319.04 1379.85 1427.02 1476.48
1571.49 1672.61 1700.75 2330.02 3220.32 3241.23 3250.69
3255.20
================================
alkyne
================================
107.00 148.12 270.83 400.80 417.63 422.77 466.92
537.98 560.29 612.17 656.29 671.46 713.03 724.83
833.19 855.52 867.96 959.22 972.80 1037.12 1123.09
1183.68 1242.38 1314.59 1315.82 1367.48 1463.46 1568.01
1659.07 1695.32 2249.53 3236.50 3237.73 3252.66 3253.51
3513.17
================================
triazole
================================
19.38 30.98 55.15 67.92 89.75 144.70 189.95 219.89
251.82 351.91 373.52 384.06 405.52 421.79 424.63
428.85 455.99 513.90 537.21 549.26 605.71 643.98
647.74 665.36 677.62 717.31 718.79 738.29 792.67
827.44 830.89 850.29 855.27 860.42 863.62 948.30
949.65 976.43 988.46 1000.92 1025.13 1037.82 1041.25
1081.98 1124.98 1127.76 1141.47 1181.26 1185.67 1258.77
1269.93 1305.38 1314.90 1317.78 1319.01 1347.86 1373.85
1392.34 1435.13 1472.54 1476.83 1514.50 1559.96 1591.42
1639.14 1671.55 1684.05 1702.72 1703.60 3217.22 3236.18
3240.27 3249.46 3250.03 3253.14 3255.81 3265.42 3330.60
================================
2
================================
5.10 7.86 11.16 17.13 20.97 28.46 43.58 44.14 50.88
52.81 59.35 59.83 73.61 78.19 89.21 100.22 101.35
105.05 114.66 117.98 133.72 138.53 142.49 159.61
180.90 192.76 196.90 214.18 217.92 230.83 232.87
240.47 250.73 252.13 253.75 255.47 265.27 271.14
275.55 278.84 281.98 286.84 293.45 301.73 306.06
317.21 353.17 361.67 365.05 367.48 384.00 394.52
403.65 410.60 423.95 428.93 441.38 455.21 469.32
478.61 480.35 488.67 497.42 510.69 512.66 533.25
554.04 574.31 599.68 625.69 640.83 651.93 656.30

667.28 677.03 689.53 706.75 725.59 726.13 744.28
768.32 790.54 806.18 810.80 829.54 845.06 851.34
854.65 859.51 866.89 892.89 911.97 915.53 915.78
918.40 936.70 939.07 939.44 940.38 955.03 957.35
965.45 967.90 980.45 981.75 982.18 983.31 993.50
1033.20 1046.39 1048.00 1065.67 1067.80 1077.48 1120.92
1134.69 1138.21 1139.22 1145.30 1151.96 1156.78 1176.54
1181.82 1192.43 1192.92 1193.92 1197.16 1197.84 1201.63
1248.61 1264.13 1274.55 1278.47 1285.76 1286.03 1288.35
1308.89 1310.30 1324.71 1334.46 1336.59 1342.51 1348.64
1356.48 1393.30 1399.55 1403.59 1407.32 1409.45 1414.24
1419.95 1427.12 1429.19 1433.14 1438.91 1451.21 1460.58
1488.05 1490.06 1490.74 1496.85 1497.29 1498.69 1501.46
1501.56 1508.20 1508.99 1509.99 1511.97 1512.98 1517.33
1517.89 1519.93 1520.17 1522.98 1559.83 1569.01 1611.34
1649.55 1666.76 1689.54 1707.44 2169.99 3048.69 3053.04
3054.26 3055.07 3055.38 3056.98 3059.25 3059.98 3060.08
3061.94 3062.74 3066.25 3072.67 3129.63 3129.92 3133.33
3134.10 3134.15 3135.23 3137.07 3138.48 3139.16 3139.37
3143.18 3150.56 3153.97 3156.83 3157.96 3165.77 3171.64
3200.91 3202.22 3206.56 3227.82 3228.98 3230.51 3238.13
3246.04 3247.24

==============================

2-TS

==============================

-210.67 2.03 11.51 14.95 20.97 24.86 25.35 32.63
33.75 39.42 43.91 49.61 50.40 54.07 60.79 70.70 73.70
75.95 82.58 90.62 97.33 105.96 111.89 114.33 124.46
127.61 135.87 143.72 153.33 160.90 172.25 180.58
188.66 194.28 207.92 215.41 217.34 231.25 231.67
239.55 240.56 243.39 249.45 253.89 261.10 267.82
269.75 275.75 285.87 290.55 292.61 293.91 305.58
309.86 323.69 349.02 353.50 359.96 362.87 377.88
384.54 395.34 400.24 411.45 417.40 425.58 425.88
430.19 434.94 436.34 449.02 465.20 469.21 471.43
479.38 496.84 508.34 513.48 514.41 517.70 529.73
534.36 543.47 566.60 600.77 634.11 635.27 639.37
650.21 657.52 658.33 668.57 675.54 684.27 703.84
711.42 721.38 727.60 751.80 772.10 792.17 806.98
808.75 814.02 826.46 829.74 845.73 854.06 854.82
855.29 865.59 870.80 890.12 893.98 916.31 916.70
919.37 919.48 930.70 933.99 937.31 938.13 945.91
958.08 959.91 967.09 970.68 974.08 978.82 980.20
980.24 980.49 994.19 1031.70 1036.19 1038.96 1044.90
1064.29 1068.23 1077.51 1122.56 1123.81 1136.90 1137.81
1139.26 1142.03 1149.92 1159.74 1171.74 1174.99 1182.35
1184.93 1190.60 1192.94 1195.17 1196.88 1199.00 1201.78
1238.31 1266.66 1274.72 1279.06 1279.97 1287.00 1289.11

1299.83 1310.04 1312.06 1313.95 1327.06 1327.47 1334.31
1336.11 1349.43 1366.01 1376.27 1390.37 1392.21 1399.43
1404.04 1406.96 1407.64 1411.99 1423.86 1424.64 1427.38
1431.04 1440.16 1452.86 1461.80 1472.71 1489.09 1490.54
1491.40 1494.06 1495.22 1500.61 1501.27 1502.93 1507.67
1510.61 1511.26 1512.84 1513.67 1516.03 1516.35 1518.30
1519.72 1523.51 1560.52 1565.26 1572.03 1614.02 1656.20
1667.08 1667.52 1691.71 1697.21 1708.31 1957.26 2025.19
3050.71 3055.74 3056.66 3058.35 3059.25 3059.47 3060.49
3061.74 3064.75 3065.59 3066.24 3066.45 3066.97 3127.93
3132.27 3133.01 3134.28 3136.07 3136.20 3136.92 3139.79
3140.45 3150.06 3151.76 3152.57 3153.63 3153.91 3160.92
3163.94 3168.87 3204.40 3205.64 3208.45 3226.00 3230.39
3231.57 3240.62 3241.84 3242.07 3246.81 3248.13 3253.23
3266.45
================================
3
================================
9.31 15.70 21.18 26.64 28.08 36.64 42.15 43.84 45.75
49.32 51.51 59.43 63.23 69.51 75.31 79.20 86.34 90.39
94.60 105.85 110.70 114.49 123.06 125.87 135.36 137.87
143.88 154.30 164.04 180.60 190.29 198.55 204.66
211.82 218.81 223.12 235.31 236.94 242.74 244.66
250.70 256.67 261.02 265.84 270.89 274.31 280.59
281.29 287.24 300.98 307.26 313.24 350.50 358.00
363.22 372.68 375.35 380.58 381.55 388.59 403.64
411.92 413.80 420.99 423.80 428.48 429.84 440.88
449.53 466.74 468.50 472.19 498.86 510.91 512.97
522.03 530.17 541.39 561.44 600.48 605.89 625.22
639.39 646.91 649.67 654.20 668.93 677.45 681.83
684.24 692.34 704.70 714.94 716.61 726.07 753.87
764.14 787.89 806.71 811.77 820.73 824.48 843.10
846.85 851.74 852.93 853.91 858.81 861.30 892.58
902.96 912.99 917.80 917.86 924.17 930.72 934.36
943.77 946.21 951.57 963.40 966.34 970.64 971.78
979.01 980.63 984.71 990.89 998.93 1011.28 1025.64
1028.42 1033.08 1036.80 1043.33 1061.22 1069.95 1079.02
1099.08 1121.59 1122.67 1124.08 1131.32 1133.88 1138.65
1150.56 1158.93 1172.14 1173.59 1173.93 1176.58 1186.87
1187.83 1192.82 1194.47 1200.16 1203.42 1217.02 1262.67
1268.54 1270.02 1273.41 1287.96 1291.49 1295.30 1307.08
1312.01 1312.25 1317.34 1321.95 1325.75 1331.18 1341.88
1351.40 1354.09 1380.65 1392.77 1394.10 1401.24 1404.85
1409.70 1415.84 1422.39 1423.81 1428.29 1433.62 1437.90
1439.35 1449.63 1453.65 1471.47 1475.50 1487.23 1489.23
1490.85 1492.37 1494.91 1498.87 1500.11 1503.50 1507.26
1510.16 1510.46 1514.10 1514.87 1517.60 1518.56 1519.79
1521.88 1522.66 1525.68 1576.93 1580.00 1586.51 1614.63

1645.16 1669.60 1677.81 1697.73 1703.77 1707.92 3000.85
3003.20 3014.42 3037.71 3041.93 3056.24 3061.24 3066.24
3068.62 3070.16 3074.00 3081.96 3096.97 3108.90 3111.53
3118.40 3125.63 3125.66 3127.04 3135.27 3136.70 3139.63
3141.22 3143.25 3150.05 3151.46 3152.10 3161.61 3164.71
3182.70 3195.58 3197.11 3200.34 3206.23 3215.13 3221.12
3228.16 3243.28 3253.33 3253.76 3266.12 3294.73 3355.04
==============================

3-TS

==============================
-1216.08 14.30 17.18 21.35 23.80 26.66 30.39 34.69
37.28 42.16 44.95 46.89 50.37 52.99 54.07 59.08 66.78
68.98 74.73 78.70 89.34 93.14 97.56 99.03 102.31
104.19 106.31 109.62 113.19 119.44 126.08 139.32
143.33 149.76 153.92 157.79 175.46 183.17 190.31
194.63 199.71 207.69 213.05 218.11 221.76 226.66
237.51 242.10 248.11 250.16 253.82 256.15 260.52
264.60 270.36 273.93 282.75 284.96 292.53 296.01
299.60 335.15 344.83 355.54 359.50 369.91 371.74
374.64 384.17 397.38 398.22 404.91 410.65 415.09
423.54 424.96 425.65 428.33 432.24 432.40 438.03
457.89 468.69 470.33 471.13 495.15 506.41 509.38
523.06 526.00 527.14 543.34 548.47 566.48 569.01
606.17 607.42 629.89 636.44 644.97 648.41 649.81
656.08 668.89 675.98 693.12 701.08 706.96 717.17
720.36 724.46 726.04 739.10 757.05 773.64 794.70
800.39 817.49 828.46 833.12 834.41 837.68 847.62
849.91 854.18 857.64 857.78 862.29 866.05 868.44
872.25 881.70 911.65 915.36 915.44 920.33 932.27
934.85 936.19 937.92 955.96 957.47 961.90 971.75
978.35 979.21 980.40 981.06 981.32 983.24 983.90
988.85 996.92 1012.62 1029.62 1035.87 1037.98 1042.52
1043.12 1045.84 1064.26 1069.23 1074.29 1117.31 1118.70
1124.60 1128.87 1129.33 1137.29 1144.03 1145.47 1155.80
1162.67 1174.00 1175.59 1179.74 1181.71 1184.21 1186.63
1189.34 1191.04 1193.63 1195.19 1200.81 1221.12 1240.78
1267.24 1271.55 1278.14 1282.12 1286.05 1291.16 1297.78
1300.83 1302.89 1309.67 1312.41 1313.35 1315.77 1329.20
1332.28 1334.66 1336.81 1337.48 1359.96 1360.14 1369.95
1370.74 1390.78 1396.24 1403.64 1408.97 1409.83 1413.24
1424.00 1425.89 1427.42 1433.61 1435.19 1441.96 1444.72
1451.18 1457.34 1462.55 1468.66 1479.39 1480.78 1485.90
1489.04 1489.78 1491.15 1494.46 1495.06 1498.52 1499.31
1501.91 1505.65 1510.76 1511.60 1511.90 1512.91 1512.94
1513.56 1517.72 1518.30 1561.27 1565.49 1576.06 1591.93
1606.27 1651.24 1663.40 1669.25 1678.27 1689.95 1692.45
1695.64 1696.93 2154.13 3047.28 3054.99 3056.65 3060.48
3062.51 3062.94 3066.47 3066.82 3068.44 3070.31 3073.17

3074.14 3083.08 3127.38 3130.44 3136.50 3137.45 3139.69
3140.94 3147.65 3149.31 3150.84 3153.35 3153.39 3155.96
3158.77 3160.36 3166.91 3173.13 3174.20 3177.74 3206.73
3211.89 3213.64 3220.65 3226.87 3235.12 3235.48 3236.59
3239.37 3240.66 3243.10 3243.17 3249.72 3250.76 3252.26
3261.18
==============================
4
==============================
4.86 12.55 14.84 21.29 25.49 27.89 32.41 39.08 41.43
42.32 45.80 51.94 53.92 59.17 60.28 67.75 68.41 74.30
79.55 85.25 91.03 93.11 96.35 101.70 103.30 110.88
114.60 118.14 123.56 126.55 129.79 139.21 145.65
150.21 160.13 163.10 174.66 184.08 194.17 201.39
206.57 209.25 217.06 220.70 226.29 228.63 233.03
237.08 244.80 248.90 254.81 259.41 261.10 271.54
274.45 278.70 283.46 286.29 295.60 299.00 305.68
328.48 356.61 360.14 363.42 372.45 375.22 376.70
378.05 381.97 386.83 402.57 415.07 419.97 423.23
426.34 428.62 429.66 432.18 447.12 465.26 468.07
470.71 475.24 476.74 481.57 496.80 508.14 514.35
519.06 541.52 543.88 565.20 566.42 605.37 607.07
620.56 633.65 637.94 645.29 648.89 657.63 672.41
678.14 683.56 689.57 693.49 716.07 720.63 726.32
727.77 739.00 754.08 758.72 759.96 793.89 804.73
817.86 825.03 831.73 835.91 845.72 846.77 848.48
852.81 856.90 858.58 860.71 867.72 868.35 875.16
912.12 913.65 915.63 917.47 933.64 935.70 937.02
938.20 939.94 956.48 964.50 969.20 970.13 979.85
980.35 980.55 983.24 983.41 984.12 985.02 996.17
1014.12 1029.10 1035.31 1037.18 1038.19 1038.72 1044.75
1052.70 1067.15 1067.86 1106.59 1118.05 1121.10 1125.18
1126.69 1127.96 1137.32 1145.42 1147.21 1148.58 1164.27
1168.60 1173.23 1175.90 1180.43 1185.20 1188.74 1192.65
1195.95 1197.97 1198.24 1213.11 1215.19 1246.53 1262.69
1266.82 1272.52 1274.78 1289.32 1294.21 1295.86 1301.94
1309.05 1310.11 1311.20 1312.72 1314.12 1330.83 1331.55
1333.07 1334.38 1335.38 1357.32 1363.48 1371.51 1374.50
1389.70 1407.38 1408.78 1410.11 1411.52 1425.60 1426.76
1430.72 1432.57 1432.99 1444.54 1448.82 1450.79 1454.91
1460.90 1468.67 1479.04 1485.76 1486.51 1489.25 1490.12
1490.58 1494.18 1494.62 1498.38 1499.58 1500.38 1507.17
1507.85 1511.17 1511.95 1514.23 1514.94 1515.44 1518.65
1519.49 1561.49 1569.04 1577.21 1595.62 1627.45 1650.76
1666.93 1678.28 1686.26 1687.60 1691.17 1695.87 1698.94
2179.60 3049.52 3058.98 3060.35 3062.07 3063.25 3063.33
3065.02 3067.77 3069.70 3070.13 3071.73 3071.86 3092.39
3094.06 3138.00 3140.07 3141.14 3148.06 3148.72 3149.55

3149.91 3150.85 3150.90 3151.88 3153.22 3158.68 3163.11
3165.09 3166.36 3166.82 3169.61 3170.34 3209.32 3212.43
3230.02 3231.61 3232.00 3233.46 3234.50 3236.21 3239.20
3243.46 3246.03 3246.23 3247.91 3250.54 3250.98 3262.26
===============================
4-TS
===============================
-390.69 10.87 15.72 22.13 23.31 26.12 29.07 33.78
40.75 41.33 46.37 51.30 53.21 60.77 61.18 68.26 69.80
71.44 73.82 76.55 82.81 85.62 91.54 92.78 96.60 104.18
106.89 110.24 117.91 121.18 131.75 136.66 145.63
152.48 155.11 159.90 167.10 183.82 191.31 200.98
203.55 219.60 221.12 222.31 229.69 237.47 240.03
246.99 247.18 254.25 257.93 261.62 262.84 262.93
267.10 271.73 275.99 285.24 291.41 293.86 323.78
340.17 345.05 350.25 361.29 372.69 378.97 386.08
393.07 398.98 400.90 404.25 407.64 423.52 424.93
426.96 433.16 433.49 441.15 448.44 451.81 467.21
473.32 477.07 493.51 496.66 501.68 513.45 524.35
526.08 530.51 536.76 554.70 562.44 598.73 606.26
631.82 638.17 640.73 644.95 646.26 652.50 655.99
672.98 676.48 683.32 697.95 716.13 719.84 723.61
723.96 744.33 746.17 777.10 789.95 805.57 812.32
826.89 827.45 831.80 835.92 843.20 844.10 850.18
853.19 854.85 857.73 863.72 870.51 872.70 884.07
911.43 913.10 918.26 920.21 921.47 934.01 934.47
936.88 940.16 943.57 955.05 960.16 970.34 971.75
978.77 979.27 980.11 980.53 984.21 986.57 999.86
1004.99 1011.51 1030.19 1030.99 1036.73 1037.87 1043.92
1044.55 1061.39 1070.41 1072.58 1073.25 1112.11 1116.70
1124.11 1130.60 1138.29 1142.66 1146.07 1156.33 1158.59
1168.46 1173.52 1176.82 1178.67 1186.37 1187.36 1192.53
1193.83 1194.10 1197.86 1200.88 1207.95 1242.62 1269.74
1271.99 1277.61 1278.38 1281.62 1293.18 1293.58 1294.33
1299.25 1304.10 1309.35 1324.29 1327.65 1329.75 1334.03
1335.65 1338.18 1351.29 1364.22 1365.83 1371.40 1378.48
1388.31 1394.77 1404.58 1409.06 1410.04 1410.66 1424.46
1425.93 1426.46 1430.63 1432.60 1436.44 1437.05 1452.32
1461.16 1463.70 1472.36 1475.15 1485.97 1487.35 1488.70
1489.67 1490.85 1496.54 1496.99 1498.67 1501.95 1506.59
1507.91 1509.72 1512.11 1512.37 1514.11 1516.18 1516.34
1520.72 1562.55 1564.66 1571.85 1582.32 1599.86 1610.89
1655.87 1663.89 1671.89 1681.72 1685.92 1689.54 1697.06
1698.88 2144.70 3051.34 3055.72 3058.31 3058.44 3061.54
3061.64 3061.91 3062.58 3062.72 3065.11 3066.35 3069.63
3118.61 3123.73 3135.17 3135.89 3138.26 3140.42 3141.21
3141.44 3143.08 3146.23 3150.47 3151.31 3151.64 3152.28
3154.04 3157.49 3164.88 3167.33 3176.99 3212.24 3213.67

3218.60 3225.00 3228.77 3234.48 3239.89 3240.63 3242.05
3242.25 3242.49 3244.15 3247.98 3249.47 3253.84 3264.14
===============================
4'
===============================
2.92 11.12 18.32 24.66 26.93 30.35 37.52 40.59 42.22
47.39 48.51 55.19 56.77 65.94 71.58 74.93 78.73 82.68
85.88 88.05 95.22 98.49 101.92 103.94 105.81 112.07
120.72 125.59 134.64 137.25 144.13 147.84 152.70
163.76 169.65 178.19 197.76 198.93 208.91 212.53
216.35 218.80 221.42 223.15 228.28 238.53 245.72
248.62 248.76 253.22 258.73 262.89 264.49 268.31
273.00 284.66 287.65 294.03 301.35 312.60 324.98
342.05 352.78 355.95 365.37 366.78 372.58 375.03
381.16 395.06 396.09 398.76 406.14 424.23 424.68
425.71 429.17 433.31 437.54 441.14 446.20 455.35
461.45 464.00 472.89 496.03 506.90 516.69 520.75
521.73 524.21 540.13 544.43 568.18 605.77 606.39
626.25 634.25 644.56 648.93 649.76 654.72 659.39
667.87 677.93 679.76 686.76 697.15 699.51 716.36
717.31 721.91 728.06 728.71 756.84 772.26 786.52
793.31 804.89 818.27 828.33 829.86 837.67 842.33
849.65 854.09 855.36 857.46 858.62 863.49 868.19
869.66 892.99 912.08 915.22 915.30 917.53 934.79
935.56 937.62 939.07 949.85 950.02 959.58 960.80
968.74 975.87 977.61 978.53 979.92 981.74 986.09
990.66 991.28 1014.76 1029.92 1035.28 1036.40 1041.67
1043.06 1050.52 1066.15 1068.66 1080.44 1091.19 1121.16
1121.94 1124.99 1135.44 1136.41 1146.47 1147.69 1148.51
1157.90 1168.81 1173.14 1174.92 1180.62 1183.73 1185.35
1193.05 1194.64 1194.84 1197.64 1198.65 1215.82 1223.87
1269.72 1274.18 1278.32 1280.04 1286.78 1290.68 1295.62
1300.19 1303.53 1309.73 1313.89 1318.43 1337.38 1338.30
1338.73 1342.00 1342.51 1362.67 1371.97 1374.58 1388.41
1388.97 1402.39 1404.80 1405.69 1408.74 1411.22 1419.92
1424.03 1428.10 1432.18 1442.60 1442.85 1448.43 1459.99
1464.00 1467.22 1478.38 1482.96 1489.94 1491.42 1493.82
1494.79 1496.34 1501.51 1503.06 1506.05 1506.39 1511.00
1511.39 1512.90 1515.55 1516.85 1517.44 1519.18 1521.68
1533.21 1558.87 1578.26 1580.64 1595.97 1612.69 1653.38
1664.45 1671.43 1682.06 1689.22 1696.77 1700.05 1701.66
1912.91 3050.19 3057.58 3060.48 3061.03 3062.76 3065.10
3066.85 3067.42 3068.21 3070.70 3073.24 3074.61 3095.00
3127.79 3130.03 3137.77 3137.88 3137.93 3139.89 3142.87
3143.08 3147.72 3150.91 3151.90 3153.42 3153.91 3155.55
3161.00 3165.19 3182.97 3205.61 3209.17 3210.24 3218.35
3233.72 3235.23 3238.42 3240.94 3241.58 3242.54 3243.01
3244.96 3249.24 3254.74 3258.81 3259.29 3261.11 3355.57

================================

4'-TS

================================

-1004.89 5.50 13.21 17.32 24.73 26.87 31.04 32.18
36.09 43.36 48.05 49.26 54.31 57.44 62.12 66.64 70.82
71.20 75.34 87.02 87.94 90.14 94.45 97.79 100.85
105.89 110.20 116.58 122.34 127.67 128.49 135.21
140.71 147.91 152.90 168.48 170.64 181.37 193.25
207.05 210.98 218.53 220.49 223.98 227.47 229.49
240.18 248.55 248.79 251.77 259.27 260.26 265.15
267.16 272.89 273.87 285.14 286.70 294.08 305.57
319.20 334.52 338.94 353.70 358.69 364.85 367.35
373.86 378.61 380.73 393.46 396.00 404.53 408.82
423.32 424.86 425.60 427.06 429.38 430.56 440.91
452.63 459.33 461.34 471.61 493.55 502.14 506.18
518.92 519.43 525.44 527.00 544.22 553.30 562.62
606.48 608.47 628.24 634.19 644.45 648.82 654.46
657.52 673.18 674.87 681.26 690.45 696.95 700.26
715.86 719.50 720.61 727.66 734.51 758.50 771.44
789.99 803.44 817.58 822.24 832.43 838.80 840.36
850.04 852.64 853.62 856.63 857.46 863.95 866.13
870.77 893.67 912.08 914.30 914.46 916.74 934.46
934.96 938.13 938.72 944.74 958.89 959.87 960.62
965.37 973.00 977.49 978.66 979.91 982.42 984.46
986.19 988.13 1020.92 1031.66 1036.26 1037.76 1041.18
1048.43 1049.44 1065.81 1068.63 1077.49 1116.85 1122.26
1122.81 1126.91 1134.23 1135.86 1146.81 1147.42 1148.89
1157.14 1159.70 1170.86 1179.00 1183.00 1183.98 1188.47
1192.84 1194.52 1194.83 1196.86 1201.80 1211.23 1226.53
1257.32 1269.48 1273.93 1276.33 1279.10 1283.96 1291.54
1296.71 1303.12 1309.86 1312.72 1315.81 1316.68 1328.13
1337.18 1337.87 1340.38 1343.15 1358.19 1366.56 1370.21
1390.41 1394.03 1400.75 1403.84 1406.44 1406.93 1407.43
1410.98 1420.46 1424.73 1427.74 1431.12 1433.40 1440.80
1451.72 1457.54 1463.27 1470.49 1484.53 1485.68 1490.70
1490.82 1493.80 1495.68 1497.32 1501.59 1503.05 1506.62
1507.35 1510.96 1511.29 1513.02 1516.06 1516.88 1517.66
1518.96 1525.68 1530.47 1562.66 1577.55 1582.64 1600.80
1615.89 1655.59 1665.69 1669.76 1683.35 1691.34 1697.00
1700.24 1707.26 2047.70 3052.97 3056.15 3059.28 3060.68
3061.38 3062.86 3065.47 3067.78 3068.12 3070.34 3073.39
3074.02 3086.27 3122.21 3133.47 3136.46 3136.82 3138.27
3138.37 3140.76 3143.70 3147.45 3150.10 3150.61 3152.07
3153.09 3153.63 3158.13 3159.69 3175.72 3197.46 3207.76
3212.93 3227.53 3234.67 3235.42 3236.13 3240.48 3240.62
3241.77 3242.39 3243.83 3249.18 3251.84 3255.13 3256.38
3260.85

================================

2+triazole

==============================

8.35 16.32 23.17 24.80 26.40 32.56 37.24 39.34 41.85
45.99 48.31 53.09 56.27 58.40 61.77 66.15 68.08 75.50
78.71 84.39 89.07 89.92 91.98 98.33 98.51 100.01
109.45 115.27 119.09 131.06 133.31 135.14 143.87
145.22 155.17 165.09 168.67 182.24 196.18 199.37
207.08 213.26 215.51 218.85 223.36 230.07 234.47
244.05 249.20 253.98 255.91 257.96 263.00 269.06
274.83 281.23 284.58 289.50 294.86 306.57 328.66
347.44 349.80 351.05 360.77 373.50 377.29 380.95
388.48 389.95 393.51 401.75 403.37 419.21 422.95
427.15 431.36 432.36 435.26 438.79 453.95 455.05
475.06 479.61 492.61 493.00 502.50 509.88 511.73
525.46 526.68 532.71 548.89 561.88 606.76 610.12
635.19 639.77 642.43 647.98 651.04 655.01 665.21
666.68 680.57 681.54 684.92 701.11 713.77 722.11
723.60 729.46 732.07 743.53 764.78 790.48 792.42
805.77 807.58 824.32 832.59 834.86 845.68 850.28
851.05 852.82 855.54 857.02 858.51 864.25 868.12
889.69 907.87 911.59 915.30 919.04 925.06 930.18
934.65 937.36 941.43 947.31 956.59 959.99 963.44
967.69 974.99 977.32 980.01 981.37 987.35 996.96
1000.35 1006.10 1026.63 1033.35 1035.96 1039.94 1044.78
1048.05 1060.58 1065.91 1070.02 1096.88 1118.65 1128.19
1128.23 1129.81 1135.18 1140.88 1143.47 1148.65 1148.92
1160.03 1174.74 1182.30 1183.70 1184.97 1186.61 1188.46
1190.47 1196.03 1197.89 1208.14 1243.19 1260.41 1266.84
1267.69 1272.61 1274.72 1278.06 1281.19 1290.33 1306.15
1308.34 1315.89 1318.28 1319.20 1322.31 1323.92 1331.85
1336.18 1343.29 1344.91 1345.71 1353.56 1369.46 1390.62
1396.15 1397.09 1404.20 1404.98 1406.40 1413.73 1422.21
1424.08 1427.22 1427.89 1437.16 1439.26 1448.68 1457.21
1473.35 1478.53 1485.26 1487.41 1491.03 1494.45 1494.79
1497.12 1498.72 1499.78 1503.72 1504.63 1507.90 1508.06
1511.84 1512.20 1515.35 1517.23 1519.02 1523.42 1526.19
1556.82 1560.81 1564.99 1589.64 1619.63 1635.66 1650.80
1663.78 1671.16 1685.87 1686.17 1699.75 1707.55 1724.12
2151.38 3030.86 3037.52 3047.95 3051.97 3055.37 3057.17
3070.01 3070.50 3076.56 3077.01 3078.91 3086.80 3088.24
3105.05 3122.87 3127.06 3132.04 3140.50 3142.70 3146.02
3148.21 3148.79 3151.91 3153.99 3155.16 3157.65 3168.47
3179.97 3187.85 3200.72 3201.45 3207.23 3207.45 3215.57
3217.47 3220.29 3232.63 3233.56 3235.75 3236.57 3237.73
3255.30 3257.24 3258.17 3259.60 3264.00 3269.32 3321.52

==============================

3_{EWG}

==============================

4.95 12.48 17.91 22.31 26.65 30.97 34.38 36.47 38.55
44.95 48.19 52.21 60.98 62.51 67.33 72.77 77.52 83.57
85.99 94.08 104.67 107.39 113.15 118.11 119.58 120.50
128.90 140.24 142.15 148.18 160.97 165.54 175.09
187.64 189.54 197.28 200.21 213.21 214.29 221.68
226.48 230.95 237.84 239.88 253.61 258.23 263.26
266.90 272.09 275.91 278.87 284.30 286.06 297.25
306.22 311.52 317.12 343.49 351.34 360.84 371.01
377.41 380.12 383.27 390.59 397.11 400.54 414.72
417.30 421.78 428.33 429.76 435.37 438.64 449.41
458.54 466.45 468.98 473.94 490.45 529.58 545.42
555.22 562.54 575.37 592.36 595.75 605.14 621.32
629.84 645.98 649.27 655.89 669.74 674.71 676.61
677.83 680.24 691.73 696.56 714.99 717.92 730.58
753.66 754.76 761.94 784.60 801.00 826.46 832.73
840.55 845.90 848.80 855.89 862.76 864.35 868.14
909.68 910.06 919.99 920.29 930.22 931.81 937.62
939.17 950.99 958.44 964.39 971.57 975.52 979.51
980.36 981.75 986.38 987.81 1011.49 1014.63 1029.84
1032.16 1035.13 1042.21 1047.59 1067.05 1068.84 1075.03
1110.12 1121.54 1125.47 1127.39 1129.66 1136.46 1139.38
1143.09 1158.75 1173.65 1178.75 1182.14 1187.98 1189.94
1192.01 1196.38 1196.95 1200.47 1241.68 1243.13 1267.12
1273.25 1279.68 1281.10 1290.90 1294.58 1302.03 1306.04
1313.41 1321.91 1324.42 1327.79 1333.12 1338.11 1347.97
1361.85 1377.86 1380.45 1398.12 1402.63 1404.19 1408.45
1418.02 1419.84 1424.36 1428.86 1430.67 1433.69 1438.85
1451.01 1452.81 1465.65 1481.68 1484.39 1484.93 1486.38
1490.39 1490.89 1493.70 1495.64 1499.33 1505.68 1507.09
1508.27 1511.61 1512.93 1515.98 1516.33 1517.34 1520.56
1521.30 1529.66 1563.91 1575.19 1587.78 1606.46 1628.36
1658.71 1666.92 1671.33 1692.03 1702.83 2333.94 2391.26
3005.66 3010.99 3012.63 3018.10 3042.06 3046.24 3050.03
3054.63 3056.91 3059.17 3078.97 3089.12 3097.54 3098.31
3109.19 3125.01 3128.11 3133.82 3134.53 3137.37 3138.26
3140.44 3144.83 3155.07 3155.43 3156.17 3170.91 3172.41
3176.67 3198.87 3203.11 3208.07 3218.43 3219.55 3227.43
3232.35 3242.66 3249.39 3265.03 3282.14 3305.52
===============================

3_{EWG}-TS

===============================

-1142.03 1.70 11.29 18.68 20.44 23.60 27.55 28.21
35.23 36.08 39.89 42.90 45.79 47.41 51.88 52.77 63.54
67.38 68.88 74.18 76.77 84.20 89.37 94.67 98.05 100.99
102.89 106.77 108.81 110.29 112.95 117.34 123.78
132.79 141.01 148.00 149.24 152.45 152.96 171.23
177.94 188.78 191.19 198.70 201.02 209.23 211.37
218.47 225.90 229.88 235.33 241.58 248.29 253.53

255.53 257.87 260.42 264.96 272.27 275.07 281.73
284.29 286.94 295.50 304.46 327.49 350.16 354.39
355.72 362.02 371.06 375.51 380.50 384.14 386.48
395.35 398.10 399.49 406.83 415.42 424.08 425.02
426.73 428.33 431.73 433.71 437.68 447.87 460.47
469.35 470.82 471.60 483.99 522.27 525.41 537.08
543.69 552.67 558.50 568.84 575.16 587.78 593.54
606.26 625.58 632.86 645.12 649.74 655.90 656.95
669.42 676.10 679.90 685.05 699.44 702.51 707.35
717.22 720.47 724.47 740.31 754.01 757.38 763.07
796.13 804.54 822.99 827.45 833.32 838.38 841.63
850.38 854.89 858.14 863.08 866.08 869.06 873.75
892.25 911.38 915.03 915.14 918.67 931.07 934.65
934.72 937.54 937.96 953.24 953.44 962.70 971.92
979.15 979.83 981.47 982.25 984.43 986.62 989.04
1012.97 1014.52 1029.38 1036.20 1037.86 1042.57 1044.62
1044.82 1067.09 1075.06 1076.35 1118.33 1118.56 1124.36
1125.60 1130.05 1134.29 1137.96 1142.98 1145.64 1166.12
1176.35 1179.42 1180.57 1182.08 1183.88 1189.39 1190.91
1194.91 1195.43 1199.26 1221.07 1241.42 1249.21 1255.57
1273.31 1281.16 1285.48 1290.98 1299.50 1300.52 1304.71
1309.81 1311.88 1314.84 1316.35 1330.67 1331.50 1334.56
1337.31 1338.84 1343.73 1360.81 1369.66 1382.79 1390.69
1406.26 1407.40 1410.17 1411.14 1414.05 1426.93 1427.37
1427.96 1428.38 1433.72 1436.04 1443.54 1443.86 1461.60
1467.71 1468.84 1469.29 1479.57 1480.39 1485.45 1488.64
1490.46 1492.55 1494.71 1498.14 1499.15 1499.62 1505.91
1511.69 1511.75 1512.29 1512.83 1513.94 1514.47 1517.79
1521.25 1561.75 1565.48 1576.12 1592.86 1607.22 1628.99
1650.95 1663.07 1664.43 1678.00 1690.57 1696.02 1696.86
2150.44 2382.02 2384.45 3045.52 3057.44 3059.57 3063.92
3064.42 3064.65 3067.69 3069.41 3069.91 3072.15 3074.94
3076.03 3091.16 3130.45 3134.27 3140.40 3141.51 3143.01
3144.27 3149.47 3151.00 3151.11 3152.81 3155.37 3155.42
3159.20 3165.47 3167.82 3168.28 3175.18 3177.76 3204.75
3218.80 3227.78 3235.04 3236.86 3237.53 3244.00 3244.07
3248.41 3250.39 3251.74 3252.40 3253.16 3261.40
==============================
4_{EWG}
==============================
10.84 12.99 18.02 18.20 25.18 28.51 29.87 37.97
38.38 41.82 43.08 47.63 49.78 53.69 60.94 67.66 69.05
72.07 75.95 80.10 81.32 89.58 92.79 96.18 99.82 102.65
107.63 111.58 114.82 119.68 124.92 127.52 135.68
139.01 145.56 147.92 151.14 155.26 161.95 165.15
171.56 177.63 196.79 198.39 206.89 213.23 216.89
222.00 224.22 228.27 233.55 236.76 243.10 247.40
253.59 261.30 262.36 270.91 276.48 279.46 283.19

290.44 296.38 299.45 309.39 332.63 354.54 360.00
364.05 366.98 373.32 375.78 377.06 380.76 385.60
387.29 390.77 403.08 413.58 415.23 423.45 427.20
428.99 430.74 432.08 438.39 441.12 467.09 468.81
472.58 473.16 475.61 478.88 516.11 530.64 544.74
545.64 546.16 566.22 571.62 590.08 605.89 611.24
617.06 635.28 637.79 645.52 648.91 652.09 658.78
671.93 679.99 681.92 688.08 692.50 693.66 715.78
719.79 727.14 736.61 737.86 754.31 755.11 767.64
773.12 813.57 819.59 831.48 838.99 849.39 851.54
854.60 856.23 859.13 862.11 865.70 870.66 911.15
912.01 913.38 913.92 915.36 917.38 934.70 935.88
937.69 938.85 940.58 953.59 965.10 968.92 972.94
973.55 980.47 982.45 983.23 984.71 985.35 1008.15
1015.22 1028.96 1031.88 1034.80 1039.13 1043.99 1046.18
1065.77 1068.76 1076.16 1094.57 1108.71 1119.15 1120.17
1125.30 1126.52 1129.80 1135.52 1146.15 1148.75 1156.52
1173.71 1176.72 1178.33 1182.19 1188.76 1191.98 1194.51
1197.89 1202.68 1215.60 1219.59 1228.02 1244.21 1244.25
1272.48 1273.71 1290.36 1296.61 1297.34 1304.22 1306.11
1307.47 1311.28 1314.31 1322.49 1330.16 1330.66 1332.00
1333.32 1334.68 1360.15 1370.24 1371.75 1389.54 1399.24
1408.64 1409.34 1410.98 1412.37 1415.49 1426.63 1428.22
1431.19 1432.89 1433.66 1439.12 1443.41 1458.22 1461.08
1462.24 1467.61 1479.65 1486.22 1486.54 1490.07 1490.37
1494.69 1495.06 1497.58 1499.92 1500.14 1508.06 1508.18
1510.90 1511.88 1513.63 1515.24 1516.82 1518.41 1520.56
1560.92 1576.02 1577.29 1596.01 1627.34 1650.00 1651.16
1665.79 1671.70 1677.89 1691.75 1695.29 1698.98 2173.70
2380.89 2383.11 3045.36 3052.99 3062.61 3063.42 3063.96
3067.54 3067.85 3069.27 3070.81 3071.06 3072.41 3072.74
3102.40 3104.90 3140.50 3141.92 3142.84 3149.43 3149.70
3150.36 3151.78 3153.12 3153.35 3153.51 3161.53 3162.46
3163.70 3168.64 3169.58 3170.67 3171.48 3173.88 3231.23
3231.60 3233.62 3238.32 3241.06 3244.19 3245.26 3246.31
3247.00 3247.38 3248.18 3249.84 3262.21 3269.23
===============================

4_{EWG}'-TS

===============================

-964.23 5.09 13.22 17.48 21.58 25.00 30.86 32.03
34.24 36.45 43.27 47.59 50.64 53.44 60.30 62.63 66.86
68.32 71.95 79.53 83.54 84.87 89.20 92.74 95.45 99.46
103.79 106.16 113.74 116.64 124.18 126.92 133.05
135.18 137.61 146.13 147.64 149.85 151.34 167.93
180.03 181.81 196.09 206.14 213.55 217.85 219.55
220.59 229.24 235.33 241.17 242.19 248.69 250.63
254.28 259.73 260.78 261.68 272.64 277.32 281.62
286.90 289.97 305.73 317.98 337.35 341.31 346.20

356.86 361.84 365.05 366.43 378.47 380.39 383.34
384.45 397.77 400.19 405.74 409.47 411.46 424.12
425.04 428.32 430.99 435.69 438.31 451.67 452.50
459.74 462.91 471.28 491.02 507.07 520.16 527.43
544.11 551.03 554.40 563.27 576.63 591.97 595.79
608.11 619.99 629.68 644.68 648.70 654.64 655.05
666.42 674.33 676.00 677.06 687.34 697.18 699.46
714.79 715.90 720.47 720.81 734.19 751.55 757.71
770.69 789.59 807.81 824.43 832.62 836.20 838.43
847.13 850.94 856.13 857.15 858.54 864.29 870.79
872.40 911.81 914.43 914.60 916.61 916.99 934.35
935.52 938.76 939.05 946.05 958.21 961.00 968.70
977.89 978.69 979.85 981.85 982.50 986.34 989.07
1014.81 1019.69 1031.11 1035.95 1037.41 1042.25 1048.09
1049.68 1067.56 1069.19 1075.18 1115.67 1121.88 1123.53
1126.47 1131.76 1134.90 1144.91 1147.44 1148.61 1155.07
1166.01 1178.68 1183.01 1183.89 1187.18 1192.85 1194.48
1194.70 1197.21 1200.60 1203.58 1225.47 1247.73 1251.21
1262.10 1274.10 1279.13 1280.64 1291.50 1297.41 1306.87
1310.04 1313.64 1315.84 1317.72 1328.42 1336.19 1337.68
1339.16 1342.09 1357.39 1366.16 1370.59 1382.81 1389.14
1399.70 1405.33 1406.16 1407.75 1410.95 1422.04 1424.60
1426.30 1429.17 1431.34 1432.08 1434.80 1453.58 1457.36
1463.80 1470.50 1483.12 1484.14 1490.25 1490.61 1493.08
1495.97 1496.62 1500.11 1501.03 1502.50 1507.16 1507.29
1511.02 1512.19 1515.65 1516.82 1517.48 1518.51 1530.54
1535.98 1563.38 1573.20 1582.51 1600.78 1617.91 1636.19
1655.22 1665.19 1675.50 1681.08 1691.48 1697.15 1699.47
2048.52 2380.27 2383.36 3050.31 3057.78 3061.12 3062.54
3063.56 3064.75 3066.21 3070.14 3071.84 3073.56 3073.69
3075.84 3086.66 3125.38 3129.83 3139.35 3139.71 3140.72
3141.76 3143.53 3144.12 3148.86 3151.70 3152.04 3152.39
3153.37 3154.82 3158.97 3159.41 3177.68 3204.18 3225.59
3235.61 3236.95 3239.39 3240.98 3242.92 3244.26 3245.52
3249.86 3252.59 3254.07 3255.52 3257.60 3260.55
==============================

3_{EDG}

==============================
3.84 12.83 20.30 21.60 25.31 30.07 33.22 40.72 44.03
47.16 49.51 53.56 57.58 62.27 62.71 69.82 73.72 80.41
85.55 96.04 103.95 109.69 110.92 119.23 124.85 127.46
128.49 132.63 139.12 147.13 159.43 165.69 173.17
180.83 187.21 193.62 200.88 205.72 210.25 214.91
217.34 221.30 226.09 228.50 232.49 246.41 248.14
252.11 256.28 260.78 263.57 270.86 274.02 275.99
280.17 291.60 293.66 295.77 300.59 324.78 338.67
343.05 350.08 357.00 362.46 369.37 376.46 383.54
390.54 400.95 405.76 414.24 422.29 429.15 429.74

433.57 450.61 461.66 467.87 469.88 481.40 496.27
512.75 528.81 544.61 556.94 562.24 575.02 591.67
600.88 605.63 620.14 633.04 646.49 649.83 655.11
665.61 673.00 676.02 677.53 681.46 691.25 702.43
711.42 714.52 717.84 754.61 764.82 776.61 786.26
790.88 813.72 829.96 830.59 843.75 847.92 854.80
854.95 856.42 859.26 866.76 910.05 913.38 913.80
917.65 929.25 930.52 932.24 932.68 955.59 958.23
968.80 973.03 975.20 975.73 976.49 977.09 978.08
986.92 1011.19 1013.54 1027.67 1033.30 1035.58 1039.02
1041.02 1065.33 1066.68 1074.31 1079.10 1095.46 1107.32
1121.51 1127.52 1128.69 1133.09 1135.30 1136.78 1140.22
1143.97 1157.33 1159.67 1172.21 1177.49 1184.65 1189.04
1189.80 1192.44 1193.27 1195.74 1199.55 1206.14 1231.83
1239.60 1261.71 1273.62 1277.57 1279.61 1283.71 1293.59
1294.81 1303.79 1307.64 1312.29 1316.49 1323.10 1324.33
1327.85 1328.46 1329.26 1334.38 1353.86 1362.10 1382.01
1395.36 1399.79 1403.72 1404.47 1407.97 1408.58 1410.58
1418.66 1422.59 1426.96 1428.06 1430.77 1443.99 1446.27
1448.66 1464.68 1465.82 1474.01 1484.08 1487.15 1488.33
1488.74 1491.39 1493.31 1494.42 1497.47 1497.60 1499.70
1499.90 1504.76 1505.64 1508.61 1509.81 1511.65 1515.05
1515.74 1516.58 1516.82 1517.78 1519.31 1521.62 1522.80
1546.31 1553.67 1576.07 1583.79 1593.47 1636.46 1643.91
1661.25 1671.20 1691.39 1699.89 1708.03 2921.72 3004.01
3012.44 3042.42 3044.47 3046.91 3051.16 3056.18 3063.03
3065.91 3066.26 3069.25 3074.38 3079.09 3094.46 3094.64
3103.54 3108.32 3111.31 3113.83 3123.33 3129.24 3136.08
3141.36 3143.08 3144.46 3146.50 3152.72 3152.90 3154.37
3156.43 3157.99 3160.13 3160.55 3165.54 3165.87 3167.69
3180.64 3192.81 3201.21 3221.86 3229.57 3230.18 3237.15
3248.42 3251.60 3252.01 3277.18 3288.23
==============================

3_{EDG}-TS

==============================

-1295.92 9.27 17.82 20.40 23.36 25.96 29.15 30.45
35.77 37.31 44.21 44.35 49.67 51.34 54.07 54.49 58.40
66.97 67.79 74.16 77.87 90.77 92.56 94.52 97.38 100.49
102.89 107.18 107.40 113.26 117.99 123.05 130.47
132.94 136.92 142.97 149.21 156.79 166.30 175.30
177.18 182.50 189.92 190.50 192.49 195.14 205.71
208.23 211.43 217.47 221.31 222.82 226.09 240.66
243.89 249.31 253.74 255.37 259.89 261.99 266.32
268.08 277.60 280.73 287.74 295.25 302.07 317.42
335.81 337.45 345.93 347.10 352.68 362.68 368.05
370.17 374.00 384.04 397.05 397.57 402.31 406.79
414.32 424.89 425.33 425.62 427.87 428.90 432.56
456.81 461.85 468.76 470.81 479.38 491.26 506.51

522.23 526.12 541.81 542.99 561.06 563.49 568.87
579.90 590.69 593.44 606.23 623.30 628.05 644.96
649.75 655.88 656.68 667.42 675.73 687.00 693.11
700.39 703.90 713.31 717.06 720.22 724.46 741.14
756.22 762.67 779.68 791.12 801.61 804.87 821.06
829.00 834.00 837.66 845.34 849.24 853.51 856.60
857.34 861.69 865.32 866.88 871.98 912.36 915.42
915.59 920.37 931.87 934.45 935.91 938.05 956.76
957.33 958.86 961.77 965.56 972.30 978.12 979.09
981.53 983.44 983.63 988.53 1012.55 1020.49 1029.68
1035.63 1038.06 1042.37 1043.86 1045.41 1066.87 1072.43
1075.25 1079.33 1098.51 1116.55 1118.38 1124.31 1125.82
1128.77 1135.28 1138.01 1143.31 1144.07 1146.55 1152.44
1162.27 1175.01 1179.89 1181.31 1183.04 1184.23 1190.00
1191.19 1192.65 1195.17 1200.93 1207.70 1221.20 1235.43
1241.37 1255.98 1271.69 1281.69 1285.85 1290.33 1292.02
1296.64 1301.70 1302.73 1309.29 1310.41 1314.12 1315.67
1319.39 1325.83 1329.44 1332.24 1334.55 1336.71 1337.30
1352.84 1359.47 1369.78 1384.27 1390.60 1397.06 1402.79
1407.34 1409.77 1412.72 1422.75 1424.83 1427.61 1431.31
1433.44 1434.85 1440.97 1442.13 1444.81 1448.21 1460.06
1461.97 1468.46 1470.18 1479.50 1486.02 1488.63 1489.47
1489.74 1491.11 1493.10 1494.08 1494.43 1498.39 1499.46
1500.46 1504.97 1505.73 1510.01 1511.03 1512.00 1512.42
1512.80 1513.64 1514.43 1517.50 1518.06 1520.22 1532.77
1561.46 1563.19 1575.76 1586.65 1591.27 1624.90 1650.83
1651.92 1663.52 1678.26 1689.85 1694.72 1696.44 1696.79
2159.68 2927.90 3025.66 3031.64 3047.68 3053.62 3054.40
3059.16 3061.29 3061.51 3065.53 3066.02 3067.75 3068.80
3072.13 3075.37 3083.35 3126.26 3127.57 3127.86 3128.06
3129.49 3134.58 3136.07 3138.47 3139.20 3146.60 3148.30
3149.34 3152.00 3152.09 3156.20 3159.20 3160.16 3166.51
3170.88 3172.62 3173.04 3173.42 3173.56 3208.08 3220.96
3225.58 3234.62 3234.65 3235.85 3241.45 3242.52 3249.19
3249.89 3252.46 3254.32 3255.33 3261.19

==============================

4$_{EDG}$

==============================

5.03 12.38 14.80 17.54 22.05 26.89 29.83 36.46 38.70
41.77 46.13 48.79 50.54 56.17 61.70 66.34 68.89 71.07
75.28 79.14 81.23 88.23 91.29 95.21 99.79 104.77
106.10 109.28 115.03 117.75 122.34 130.35 132.03
134.81 145.36 146.27 150.70 153.55 159.17 169.43
171.89 175.00 180.32 186.53 197.97 201.77 209.35
217.53 220.89 223.88 229.56 230.54 237.39 241.56
245.06 247.30 257.40 260.62 263.99 271.10 275.51
279.00 280.30 287.51 288.77 298.35 307.45 309.80
332.40 341.99 352.32 358.96 364.47 367.93 369.24

372.60 375.79 377.75 381.09 386.07 402.41 414.61
423.03 425.42 428.50 429.71 430.78 432.50 439.80
466.29 469.17 470.47 472.84 474.99 482.68 491.82
515.28 544.01 545.59 561.29 565.88 568.82 570.45
576.94 605.56 613.44 615.02 636.42 638.27 645.52
649.02 658.19 664.44 672.30 684.66 692.03 692.47
693.72 698.93 715.34 719.73 728.38 737.03 743.64
753.79 767.05 777.06 792.04 811.16 816.62 830.64
832.01 835.51 837.01 848.37 852.73 856.44 858.39
860.59 861.07 868.83 870.82 911.98 912.22 914.32
917.38 933.05 935.09 935.62 938.06 940.07 947.55
949.06 958.78 964.84 970.96 975.78 980.33 982.50
983.47 984.90 985.11 1014.69 1018.06 1029.09 1034.57
1035.02 1038.49 1043.67 1045.48 1066.16 1068.43 1070.28
1074.29 1097.13 1106.15 1108.09 1119.39 1120.88 1125.55
1125.76 1129.82 1134.55 1144.39 1146.63 1148.09 1149.10
1150.65 1173.00 1176.72 1180.55 1183.75 1188.71 1192.58
1194.66 1197.92 1199.21 1204.43 1210.62 1215.35 1226.65
1246.19 1246.35 1272.10 1272.96 1284.68 1289.84 1294.55
1298.10 1301.41 1308.57 1309.03 1310.57 1311.61 1312.81
1330.77 1331.17 1331.76 1334.27 1335.03 1336.05 1337.32
1357.66 1365.85 1371.01 1388.19 1389.72 1406.85 1408.23
1409.39 1411.84 1424.62 1427.30 1430.00 1431.83 1433.00
1441.52 1443.78 1445.98 1450.86 1455.26 1458.08 1459.72
1467.59 1469.15 1479.24 1486.58 1486.89 1489.14 1490.89
1491.23 1491.84 1494.18 1494.54 1495.24 1499.78 1500.13
1507.68 1508.21 1509.91 1511.21 1511.83 1512.37 1514.06
1515.74 1516.14 1517.73 1518.22 1520.21 1531.65 1555.81
1562.14 1577.12 1595.46 1600.74 1639.83 1651.57 1666.95
1670.25 1678.21 1680.90 1690.89 1695.20 1699.07 2177.24
2941.78 3030.13 3033.01 3045.78 3054.50 3059.57 3060.58
3061.52 3064.30 3065.30 3065.65 3067.53 3067.87 3070.00
3070.36 3097.55 3098.77 3121.82 3128.33 3134.68 3136.34
3137.91 3139.30 3146.39 3147.37 3148.37 3148.91 3149.20
3150.49 3150.97 3160.52 3160.72 3163.55 3164.15 3165.06
3167.47 3168.49 3169.36 3169.54 3171.60 3230.77 3230.88
3232.58 3235.72 3239.63 3242.96 3244.25 3245.35 3247.56
3248.21 3249.69 3250.23 3262.51 3262.65
===============================
$4_{EDG'}$-TS
===============================
-1010.30 4.51 16.08 17.87 23.95 25.76 30.67 30.87
34.98 44.77 46.70 50.09 53.23 55.57 58.88 62.63 66.48
68.77 71.78 81.98 85.34 87.45 91.22 95.30 97.85 103.23
106.22 108.57 116.30 117.11 124.51 127.46 133.21
137.20 140.36 148.38 150.72 157.73 168.96 174.45
180.75 187.93 190.68 199.37 209.45 214.76 216.78
219.68 220.94 224.09 229.60 234.40 241.45 245.41

248.87 251.01 253.67 259.39 260.28 264.52 266.96
269.78 271.14 286.26 288.53 305.86 308.75 330.47
334.05 336.63 340.00 347.81 357.64 361.51 362.15
367.98 372.71 379.31 381.32 392.18 396.22 405.72
408.20 421.65 423.98 424.59 425.99 426.31 430.75
453.01 455.61 458.95 464.90 490.07 491.76 506.88
518.26 521.91 529.11 543.76 553.57 558.69 559.72
570.74 590.53 604.91 608.11 618.50 626.64 643.82
648.89 652.42 654.92 664.54 675.41 678.58 683.24
688.29 694.60 705.94 710.55 715.28 718.56 720.88
735.46 757.52 764.88 779.31 787.03 790.83 815.86
820.60 826.36 832.52 838.49 848.87 852.89 855.30
856.25 859.32 863.46 867.80 870.81 911.60 914.37
914.83 916.94 933.36 935.44 936.94 938.27 945.84
959.60 960.96 961.65 966.58 975.02 977.89 978.53
980.08 982.29 986.40 987.31 1016.17 1020.77 1031.46
1036.19 1037.56 1040.64 1047.92 1049.48 1065.40 1069.78
1074.70 1082.43 1100.02 1114.05 1122.24 1122.71 1127.11
1131.40 1135.26 1141.24 1143.11 1147.21 1148.22 1156.40
1156.73 1162.80 1179.47 1182.82 1184.12 1187.18 1192.80
1194.22 1194.73 1196.81 1197.29 1205.87 1211.51 1226.34
1241.06 1256.90 1263.07 1272.83 1277.43 1277.76 1286.42
1291.83 1294.06 1297.21 1309.86 1312.08 1313.81 1315.48
1318.44 1321.10 1328.69 1337.67 1337.85 1341.43 1343.92
1359.28 1367.50 1370.39 1391.09 1395.66 1401.98 1405.28
1406.28 1407.71 1408.71 1410.58 1415.22 1418.85 1424.48
1426.58 1430.97 1434.73 1445.41 1448.78 1454.87 1461.59
1462.91 1470.22 1476.70 1484.75 1485.25 1490.71 1490.92
1493.46 1493.93 1495.30 1495.71 1496.79 1501.23 1502.98
1505.86 1506.67 1508.07 1511.08 1512.29 1515.95 1517.23
1517.61 1519.11 1519.80 1521.61 1525.46 1531.06 1547.22
1559.85 1562.54 1579.55 1586.67 1600.61 1637.95 1645.85
1655.88 1665.52 1684.11 1691.12 1696.71 1697.55 1707.33
2041.35 2976.83 3025.04 3036.87 3051.76 3054.54 3057.03
3059.35 3059.89 3061.50 3064.64 3065.02 3069.53 3070.67
3072.95 3073.95 3088.88 3108.46 3124.37 3126.75 3127.51
3132.33 3133.51 3134.39 3135.56 3137.40 3138.68 3142.61
3146.56 3149.08 3149.62 3150.81 3152.16 3153.50 3157.38
3159.45 3164.96 3168.99 3174.96 3192.95 3226.42 3235.01
3235.69 3237.22 3239.76 3242.43 3243.06 3248.83 3250.67
3251.40 3255.24 3258.48 3260.12 3262.88

References

[1] van Beek, C. B.; van Leest, N. P.; Lutz, M.; de Vos, S. D.; Klein Gebbink, R. J. M.; de Bruin, B.; Broere, D. L. J. Combining metal–metal cooperativity, metal–ligand cooperativity and chemical non-innocence in diiron carbonyl complexes. *Chem. Sci.* **2022**, *13* (7), 2094–2104.

[2] Zhao, J.-W.; Guo, J.-W.; Huang, M.-J.; You, Y.-Z.; Wu, Z.-H.; Liu, H.-M.; Huang, L.-H. Design, synthesis and biological evaluation of new steroidal β-triazoly enones as potent antiproliferative agents. *Steroids* **2019**, *150*, 108431. **Note:** The extraction was performed with the more volatile Et_2O as a solvent instead of ethyl acetate, due to the volatility of the azide product.

[3] Brookhart, M.; Grant, B.; Volpe, A. F. $[(3,5\text{-}(CF_3)_2C_6H_3)_4B]^-[H(OEt_2)_2]^+$: a convenient reagent for generation and stabilization of cationic, highly electrophilic organometallic complexes. *Organometallics* **1992**, *11* (11), 3920–3922.

[4] Bew, S. P.; Hiatt-Gipson, G. D.; Lovell, J. A.; Poullain, C. Mild reaction conditions for the terminal deuteration of alkynes. *Org. Lett.* **2012**, *14* (2), 456–459.

[5] Fulmer, G. R.; Miller, A. J. M.; Sherden, N. H.; Gottlieb, H. E.; Nudelman, A.; Stoltz, B. M.; Bercaw, J. E.; Goldberg, K. I. NMR chemical shifts of trace impurities: Common laboratory solvents, organics, and gases in deuterated solvents relevant to the organometallic chemist. *Organometallics* **2010**, *29* (9), 2176–2179.

[6] Kounalis, E.; Lutz, M.; Broere, D. L. J. Tuning the bonding of a μ-mesityl ligand on dicopper(I) through a proton-responsive expanded PNNP pincer ligand. *Organometallics* **2020**, *39* (4), 585–592.

[7] Kirmse, W.; Horner, L. Umsetzung von phenylacetylen mit aziden und diazoverbindungen. *Liebigs Ann. Chem.* **1958**, *614* (1), 1–3.

[8] Creary, X.; Anderson, A.; Brophy, C.; Crowell, F.; Funk, Z. Method for assigning structure of 1,2,3-triazoles. *J. Org. Chem.* **2012**, *77* (19), 8756–8761.

[9] Schreurs, A. M. M.; Xian, X. ; Kroon-Batenburg, L. M. J. *EVAL15*: a diffraction data integration method based on *ab initio* predicted profiles. *J. Appl. Cryst.* **2010**, *43* (1), 70–82.

[10] Sheldrick, G. M. SADABS and TWINABS. **2014**, Universität Göttingen, Germany.

[11] Sheldrick, G. M. *SHELXT* - Integrated space-group and crystal-structure determination. *Acta Cryst.* **2015**, *A71* (1), 3–8.

[12] Sheldrick, G. M. Crystal structure refinement with *SHELXL*. *Acta Cryst.* **2015**, *C71* (1), 3–8.

[13] Spek, A. L. *PLATON* SQUEEZE: a tool for the calculation of the disordered solvent contribution to the calculated structure factors. *Acta Cryst.* **2015**, *C71* (1), 9–18.

[14] Spek, A. L. Structure validation in chemical crystallography. *Acta Cryst.* **2009**, *D65* (2), 148–155.

[15] Parr, R. G.; Yang, W. *Density-functional theory of atoms and molecules*; Oxford University Press: New York, 1989.

[16] Frisch, M. J.; Trucks, G. W.; Schlegel, H. B.; Scuseria, G. E.; Robb, M. A.; Cheeseman, J. R.; Scalmani, G.; Barone, V.; Mennucci, B.; Petersson, G. A.; Nakatsuji, H.; Caricato, M.; Li, X.; Hratchian, H. P.; Izmaylov, A. F.; Bloino, J.; Zheng, G.; Sonnenberg, J. L.; Hada, M.; Ehara, M.; Toyota, K.; Fukuda, R.; Hasegawa, J.; Ishida, M.; Nakajima, T.; Honda, Y.; Kitao, O.; Nakai, H.; Vreven, T.; Montgomery, J. A., Jr; Peralta, J. E.; Ogliaro, F.; Bearpark, M. J.; Heyd, J. J.; Brothers, E. N.; Kudin, K. N.; Staroverov, V. N.; Keith, T. A.; Kobayashi, R.; Normand, J.; Raghavachari, K.; Rendell, A. P.; Burant, J. C.; Iyengar, S. S.; Tomasi, J.; Cossi, M.; Rega, N.; Millam, J. M.; Klene, M.; Knox, J. E.; Cross, J. B.; Bakken, V.; Adamo, C.; Jaramillo, J.; Gomperts, R.; Stratmann, R. E.; Yazyev, O.; Austinm, A. J.; Cammi, R.; Pomelli, C.; Ochterski, J. W.; Martin, R. L.; Morokuma, K.; Zakrzewski, V. G.; Voth, G. A.; Salvador, P.; Dannesberg, J. J.; Dapprich, S.; Daniels, A. D.; Farkas, O.; Foresman, J. B.; Ortiz, J. V.; Cioslowski, J.; Fox, D. J. *Gaussian 09, Revision A.02*; Gaussian, Inc., Wallingford CT, 2016.

[17] Adamo, C.; Barone, V. Toward reliable density functional methods without adjustable parameters: The PBE0 model. *J. Chem. Phys.* **1999**, *110* (13), 6158–6170.

[18] (a) Slater, J. C.; Philipd J. C. Quantum theory of molecules and solids. vol. 4: The self-consistent field for molecules and solids. *Phys. Today*, **1974**, *27* (12), 49–50. (b) Vosko, S. H.; Wilk, L.; Nusair, M. Accurate spin-dependent electron liquid correlation energies for local spin density calculations: A critical analysis. *Can. J. Phys.* **1980**, *58* (8), 1200–1211; (c) Grimme, S.; Antony, J.; Ehrlich, S.; Krieg, H. A Consistent and accurate ab initio parametrization of density functional dispersion correction (DFT-D) for the 94 elements H-Pu. *J. Chem. Phys.* **2010**, *132* (15), 154104–154119; (d) Grimme, S.; Ehrlich, S.; Goerigk, L. Effect of the damping function in dispersion corrected density functional theory. *J. Comput. Chem.* **2011**, *32* (7), 1456–1465.

[19] (a) Ditchfield, R.; Hehre, W. J.; Pople, J. A. Self-consistent molecular-orbital methods. IX. An extended gaussian-type basis for molecular-orbital studies of organic molecules. *J. Chem. Phys.* **1971**, *54* (2), 724–728; (b) Hehre, W. J.; Pople, J. A. Self-consistent molecular orbital methods. XIII. An extended gaussian-type basis for boron. *J. Chem.*

Phys. **1972**, *56* (8), 4233–4234; (c) Binkley, J. S.; Pople, J. A. Self-consistent molecular orbital methods. XIX. Split-valence gaussian-type basis sets for beryllium. *J. Chem. Phys.* **1977**, *66* (2), 879–880; (d) Hariharan, P. C.; Pople, J. A. The influence of polarization functions on molecular orbital hydrogenation energies. *Theor. Chim. Acta* **1973**, *28* (3), 213–222; (e) Hehre, W. J.; Ditchfield, R.; Pople, J. A. Self—consistent molecular orbital methods. XII. Further extensions of gaussian—type basis sets for use in molecular orbital studies of organic molecules. *J. Chem. Phys.* **1972**, *56* (5), 2257–2261; (f) Francl, M. M.; Pietro, W. J.; Hehre, W. J.; Binkley, J. S. Self-consistent molecular orbital methods. XXIII. A polarization-type basis set for second-row elements. *J. Chem. Phys.* **1982**, *77* (7), 3654–3665.

[20] Hay, P. J.; Wadt, W. R. Ab initio effective core potentials for molecular calculations. Potentials for the transition metal atoms Sc to Hg. *J. Chem. Phys.* **1985**, *82* (1), 270–283.

[21] (a) Clark, T.; Chandrasekhar, J.; Spitznagel, G. W.; Schleyer, P. V. R. Efficient diffuse function-augmented basis sets for anion calculations. III. The 3-21+G basis set for first-row elements, Li–F. *J. Comput. Chem.* **1983**, *4* (3), 294–301; (b) Frisch, M. J.; Pople, J. A.; Binkley, J. S. Self-consistent molecular orbital methods 25. Supplementary functions for gaussian basis sets. *J. Chem. Phys.* **1984**, *80* (7), 3265–3269; (c) McLean, A. D.; Chandler, G. S. Contracted gaussian basis sets for molecular calculations. I. Second row atoms, Z= 11–18. *J. Chem. Phys.* **1980**, *72* (10), 5639–5648; (d) Krishnan, R.; Binkley, J. S.; Seeger, R.; Pople, J. A. Self-consistent molecular orbital methods. XX. A basis set for correlated wave functions. *J. Chem. Phys.* **1980**, *72* (1), 650–654.

[22] Küchle, W.; Dolg, M.; Stoll, H.; Preuss, H. Energy-adjusted pseudopotentials for the actinides. Parameter sets and test calculations for thorium and thorium monoxide. *J. Chem. Phys.* **1994**, *100* (10), 7535–7542.

[23] Marenich, A. V.; Cramer, C. J.; Truhlar, D. G. Universal solvation model based on solute electron density and on a continuum model of the solvent defined by the bulk dielectric constant and atomic surface tensions. *J. Phys. Chem. B* **2009**, *113* (18), 6378–6396.

[24] Nicolay, A.; Héron, J.; Shin, C.; Kuramarohit, S.; Ziegler, M. S.; Balcells, D.; Tilley, T. D. Unsymmetrical Naphthyridine‐Based Dicopper(I) Complexes: Synthesis, Stability, and Carbon–Hydrogen Bond Activations. *Organometallics* **2021**, *40* (12), 1866–1873.
